# Developing C2-Aroyl Indoles as Novel Inhibitors of IDO1 and Understanding Their Mechanism of Inhibition *via* Mass Spectroscopy, QM/MM Calculations and Molecular Dynamics Simulation

**DOI:** 10.3389/fchem.2021.691319

**Published:** 2021-07-15

**Authors:** Jyoti Chauhan, Srinivas R. Maddi, Kshatresh Dutta Dubey, Subhabrata Sen

**Affiliations:** ^1^ Department of Chemistry, School of Natural Sciences, Shiv Nadar University, Greater Noida, India; ^2^ Acubiosys PVT LTD, TBI, BITS-Pilani Campus, Hyderabad, India

**Keywords:** molecular dynamic simulation, QM/ MM calculation, mass spectroscopic analysis, indoleamine (2, 3)-dioxygenase, C2 substituted indoles

## Abstract

Indoleamine-2,3-dioxygenase (IDO1) and tryptophan dioxygenases are two heme based metalloenzymes that catalyze the tryptophan oxidation reaction by inserting molecular dioxygen to cleave the pyrrole ring. The mechanism of such ring cleavage reaction is of carcinogenic importance as the malignant tumors recruit this mechanism for immune invasion. In the presence study, we have synthesized a Novel C2 aroyl indoles inhibitor, **8d,** which shows significant inhibition of 180 nM at IC_50_ scale. The binding and conformational changes that transpire after inhibitor binding were thoroughly studied by molecular docking and MD simulations. The subsequent QM/MM (Quantum Mechanical/Molecular Mechanical) calculations were used to proposed the mechanism of inhibition. The QM/MM calculations show that the reaction proceeds *via* multistep processes where the dioxygen insertion to the substrate **8a** is the rate determining process. Theoretical mechanism is further supported by mass spectroscopy, and drug metabolism/pharmacokinetics study (DMPK) and metabolic stability of compound **8d** was investigated in rat and human liver microsomes.

## Introduction

The transformation of l-tryptophan (L-tryp) to kynurenine is an essential chemical reaction which is im-plicated in various biological process and in turn can be exploited as a therapeutic options in cancer, neurodegeneration, aging and immunity (Dunn et al., 2004; Munn and Mellor, 2007; Kershaw et al., 2014; Sas et al., 2018). Indoleamine-2, 3-dioxygenase 1 (IDO1) catalyzes the initial and rate limiting step of this reaction (Pradhan et al., 2017). Despite exhibiting potential as a therapeutic target in myriad diseases, IDO1 is mainly implicated in cancer (Okamoto et al., 2005; Zou, 2005; Sugimoto et al., 2006; Mazarei and Leavitt, 2015; Song et al., 2018).

**FIGURE 1:**
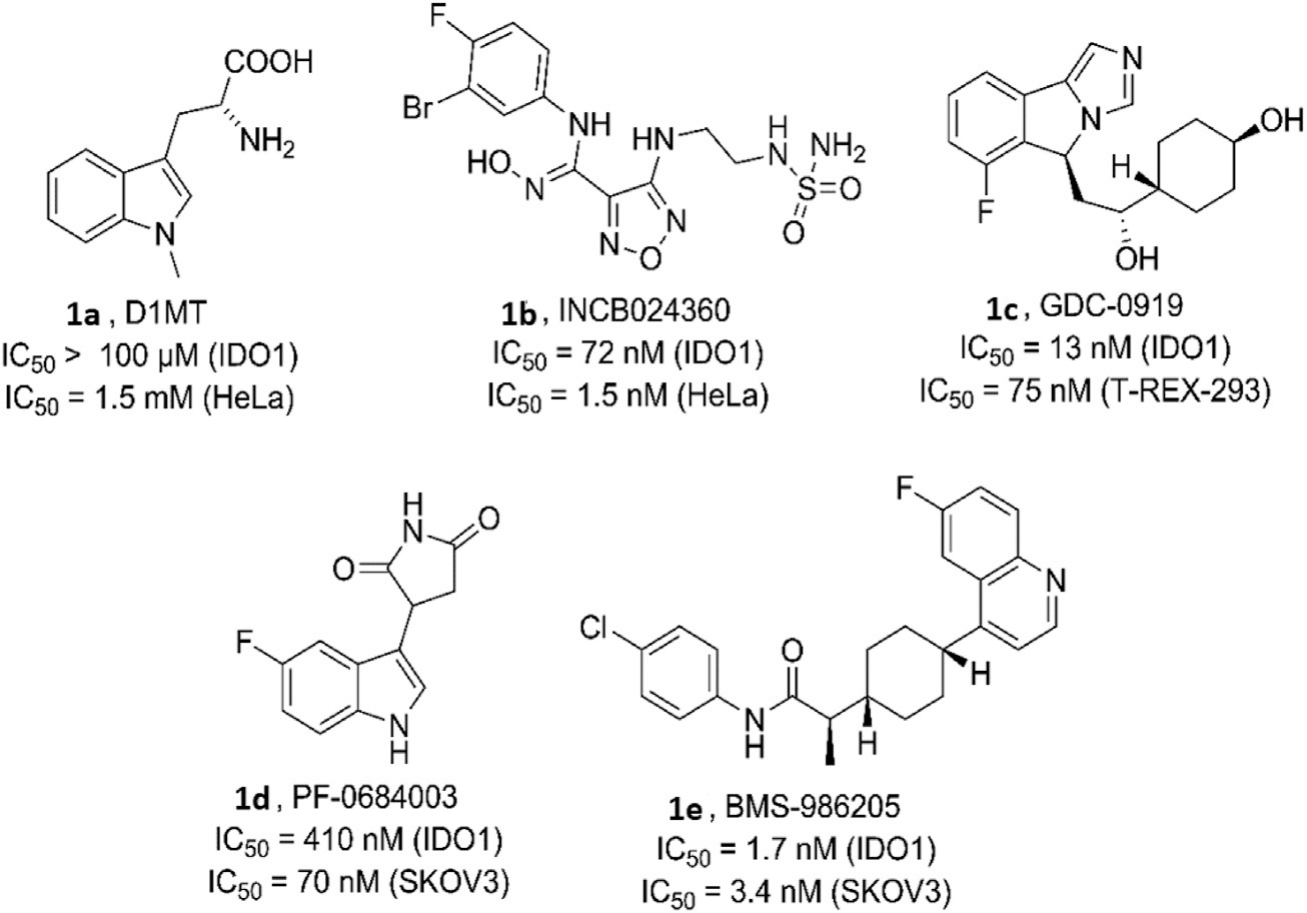
Human indoleamine-2, 3-dioxgenase (hIDO) inhibitors as clinical candidates.

Various bioactive small molecules belonging to different classes of heterocycles have been reported as IDO1 inhibitors (Dolušić and Frédérick, 2013; Muller et al., 2005). Interestingly among these plethora of reported IDO1 inhibitors only few have hitherto entered clinical trials. Representatively, 1-methyl-d-tryptophan **1a** (DIMT) (Indoximod) (Figure 1) is the first clinical candidate, with an IC_50_ of ∼100 μM against hIDO1 and IC_50_ of 1.5 mM against HeLa and HT29 cells respectively (Vacchelli et al., 2014). The next to enter clinical trial was Incyte Corporation’s Epocodostat **1b** belonging to hydroxiamidine class of compounds which inhibited IDO1 with an IC_50_ of 72 nM (Figure 1). (Prendergast et al., 2017) Another clinical candidate (phase I), the imidazole Novoximid, **1c** evolved from Newlink-Genentech’s combined effort (Figure 1). It inhibits IDO1 and T-REX-293 IDO1 with IC_50_ of 13 and 75 nM respectively (Li et al., 2017). In 2018, two more molecules, Pfizer-iTeos′ indole derivative, **1d** and Bristol Myers Squibb’s quinoline compound **1e** entered the clinical trials (Figure 1) (Siu et al., 2017; Wen et al., 2019).

Despite recent failures of clinical candidates targeting IDO1, the new emerging up regulation of TDO and the advent of the combination therapy, the interest in IDO1 inhibitors as a potential anticancer drug has not deterred (Van den Eynde et al., 2020). There are still several articles reporting the design, synthesis and *in vitro*/*in vivo* evaluation of IDO1 inhibitors. However such “*run-of-the-mill*” reports does not help in rectifying the shortcomings that led to the road block for the development of IDO1 inhibitors as successful clinical candidates, as it is important to conduct studies that investigate the underlying mechanism of action of these small molecules with the heme protein (IDO1). Critical analysis of the metabolites generated during the reaction could suggest novel molecules potentially better than the present IDO1 inhibitors. There has been reports of thorough investigation on the mechanism by which the two heme enzymes IDO1 and TDO2 catalyzes the degradation of l-tryptophan (Chauhan et al., 2009; Basran et al., 2011). There is also a study published by Röhrig *et al.* that included investigation of mechanism of action of four known IDO1 lead molecules through computational and experimental approaches (Matin et al., 2006; Paul et al., 2016; Röhrig et al., 2019). Surprisingly, similar studies to understand the interaction of IDO1 or TDO2 with novel inhibitors are missing from the literature. Herein we report our investigation of IDO1 catalyzed degradation of compound **8d**, a C2-aroyl indole based novel inhibitor of IDO1, by UV absorbance spectroscopy, Quantum mechanics/molecular mechanics (QM/MM) calculations and molecular dynamic (MD) simulations. The outcome of the computational experimentation was corroborated by the mass spectroscopy results and afforded further substantiation of the mechanism. Additionally, DMPK studies of **8d** and two other potent C2-aroyl indoles revealed the druggable attributes of these compounds.

**FIGURE 2:**
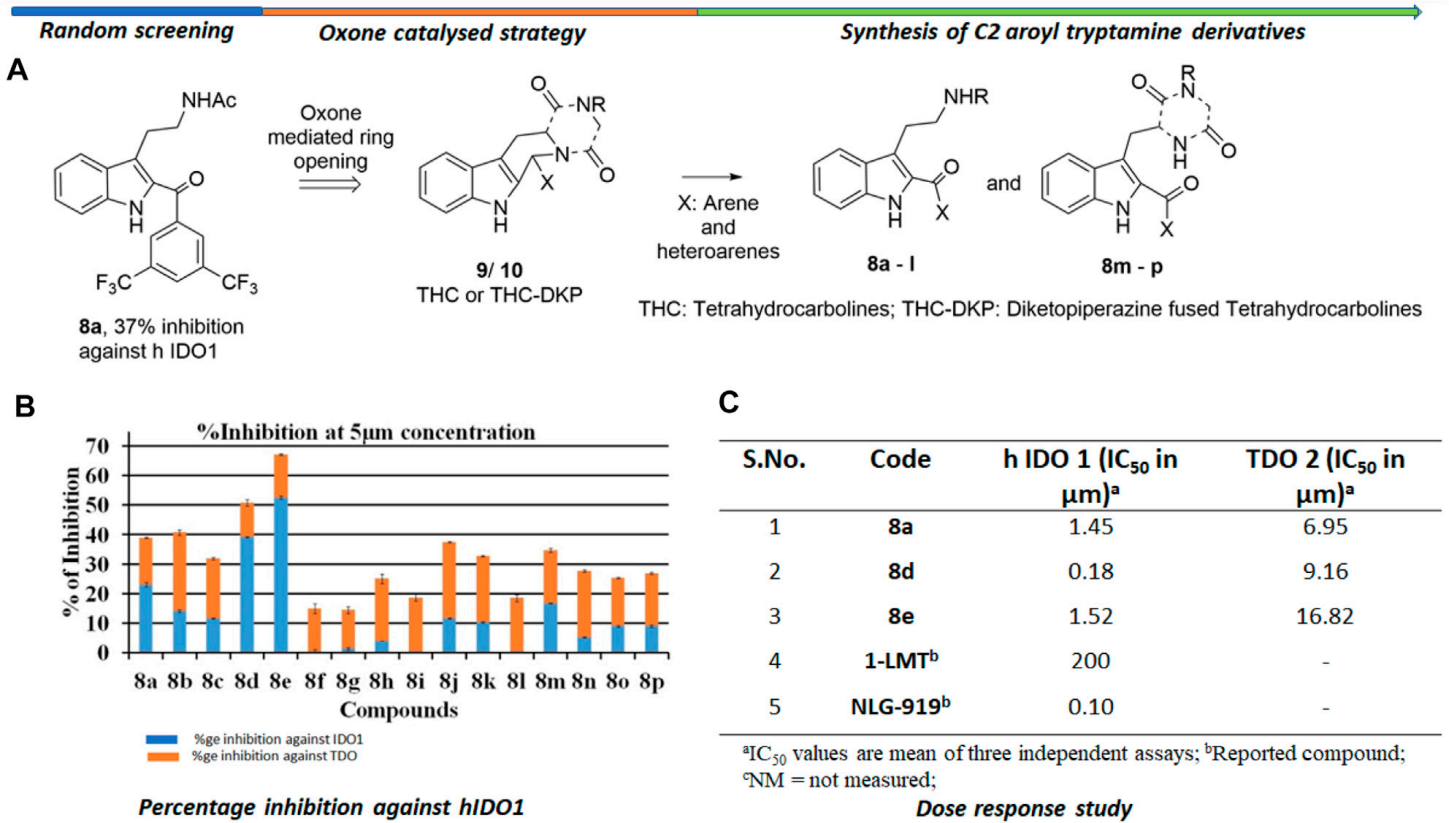
Background study of the evolution of compound **8d** as a IDO one inhibitor: **(A)** Design and synthesis of C2 aroyl tryptamine derivatives **8b** → **p**; **(B)** Percentage inhibition induced by compounds **8a** → **p** on IDO1 at 5 μM concentration; **(C)** Dose response study of compounds **8a**, **d**, and **e**. For X and R, please refer to the supplementary information, Supplementary Figure S2.

The background of the discovery of compound **8d** (Figure 2) as a novel selective IDO1 inhibitor (over TDO2) involved random screening of various inhouse compounds against recombinant protein of human IDO1 enzyme (percentage inhibition at 5 μM concentration (Supplementary Figure S1 [refer *SI*]). The molecule that provided the best inhibition from this preliminary screening was the C2-aroyl tryptamine derivative **8a** that inhibited IDO1 to ∼40% (Figure 2) (Supplementary Figure S1 [refer *SI*]. A library based on **8a** was generated through a novel oxone mediated green oxidative ring opening of tetrahydrocarbolines **9** and **10** (Figure 2) (Supplementary Table S1 and Supplementary Figure S2 [Refer *SI*]). Percentage inhibition screening of this library at 5 μM concentration of the compounds and subsequent dose response study provided compounds **8a**, **d** and **e** that induced substantial inhibition to IDO1 with IC_50_ of 1.45, 0.18 and 1.52 μM respectively compared to 200 μM by 1-LMT and 0.1 μM by NLG-919, which were used as the positive standards in the screening (Figure 2). The IC_50_ of compounds **8a**, **d** and **e** against recombinant protein of human TDO2 enzyme were 6.95, 9.16, and 16.82 μM respectively (Figure 2). Based upon the biological studies, compound **8d** emerged as the most potent molecule of our molecular library against IDO with reasonable selectivity over TDO. To understand the mechanism of action of our molecules compound 8d was selected for UV-Vis absorption, reaction kinetics and mass spectroscopic investigation. Additionally, compound 8d was used in the QM/MM calculations and molecular dynamic (MD) simulations.

## Materials and Methods

### Experimental Section

#### Materials and Instruments

All reactions were carried out under N_2_ atmosphere as specified. Column chromatography was performed on Silica gel (60–120 mesh), and reactions was monitored by thin layer chromatography (TLC, Silica gel 60 F_254_), using UV light to visualize the course of the reaction. ^1^H NMR and ^13^C NMR spectra were recorded with tetramethylsilane as an internal standard at ambient temperature unless otherwise indicated with Bruker instruments at 400 MHz for ^1^H NMR and 100 MHz for ^13^C NMR spectroscopy. Splitting patterns are designated as singlet (s), broad singlet (br, s), doublet (d), triplet (t). Splitting patterns that could not be interpreted or easily visualized are designated as multiplet (m). Mass spectrometry analysis was done with a 6,540 Accurate-Mass QTOF LC-MS system (Agilent Technologies) equipped with an Agilent 6540 LC system. Accurate mass analysis calibration was carried out by ESI-low concentration tuning mix solution provided by Agilent technologies, United States. High performance liquid chromatography (HPLC) experiments were carried out on Agilent 1,290 infinity series of LC system (Agilent Technologies, United States) with photodiode-array/ELSD detector to monitor the purity of compounds. High performance liquid chromatography (HPLC) experiments were carried out on a Waters Alliance System (Milford, MA) consisting of e2695 separation module and a 2,998 photodiode-array detector. The HPLC system was controlled with EMPOWER software (Waters Corporation, Milford, MA) obtained by the Department of Chemistry, School of Natural Sciences, Shiv Nadar University, Uttar Pradesh 201,314, India.

General procedure for preparation of the target compounds **8a** - **p**.

The oxone (1.2 equiv.) was added to a solution of **9a** - **n** or **10a** - **d** (1.0 equiv.) and KBr (0.05 equiv.) in MeCN/H_2_O (10/1) at 0°C (see SI for the preparation of (**9a** - **n** or **10a**—**d**). The reaction mixture was allowed to warm to r. t. and was stirred for another 2–12 h. Once TLC confirmed the complete consumption of the starting material, the reaction was quenched with aqueous saturated sodium bicarbonate (aq. sat. NaHCO_3_) and aqueous saturated sodium sulfite (aq. sat. Na_2_SO_3_) followed by diluting it with ethyl acetate (EtOAc). The organic fractions were collected, and the aqueous phase was extracted twice with ethyl acetate. The combined organic fractions were washed with brine, dried over anhydrous magnesium sulfate (anh. MgSO_4_), filtered, and concentrated under reduced pressure. The resulting residue was purified by flash column chromatography (EtOAc/hexane = 1:10 → 1:1) to provide the desired C2-aroyl compounds. A comprehensive protocol for the synthesis of compounds **8a**—**p is discussed in SI.**

#### Enzymatic Assay *In-Vitro*

The enzyme culture materials Potassium phosphate, dipotassium hydrogen phosphate, 1-methyl-l-tryptophan, l-kynurenine, l-tryptophan, catalase, trichloroacetic acid (TCA), *p*-Dimethylaminobenzaldehyde (Ehrlich’s reagent) and sodium dithionate were purchased from sigma Aldrich and methylene blue, dimethyl sulfoxide (DMSO), glacial acetic acid, ascorbic acid, glycerol and tris-HCL buffer were purchased from Sisco Research Laboratories, India. The recombinant human IDO/TDO protein were purchased from Biovision (Catalog no.: P1096 human recombinant IDO1 and CSB-YP023351HU human recombinant TDO2). All the other HPLC grade solvent were purchased from Merck Pvt. Ltd. The IDO/TDO assays were performed according to the reported procedures.1 and 2, the details of which is mentioned in the SI.

#### UV-Vis Absorption

All the measurements of binding of compound and enzyme were recorded at room temperature using an Agilent Cary 8454 UV-Vis diode array spectrophotometer with 1 cm light path. The experiments were performed in 50 mM potassium phosphate buffer pH- 6.5 with IDO1 protein concentration of 1 µM and compound concentration of 25 µM was incubate for 3 h at 37°C. The deoxy-form of protein was prepared by pre-purged with nitrogen gas and adding sodium dithionate (∼5 fold excess) in protein with potassium phosphate system.

#### Inhibition Kinetics

The kinetic mode of inhibition of highest active compound against indolamine-2, 3-dioxygenase one was determined by series of test solution in which the concentrations of respective substrate l-tryptophan (1–4 mM) was varied in presence of different concentrations of the inhibitors (0–1.5 µM). The IDO1 activity assay was performed to measure the formation of N-formylkynurenine by absorbance plate reader at 492 nm. The mode of inhibition of the screened compounds was determined from the Lineweaver-Burk plots, which are the double reciprocal plots of enzyme reaction velocities (1/V) vs. substrate concentrations (1/[S]), where V and [S] represent the initial rate of enzymatic reaction and l-tryptophan concentration respectively.

#### Mass Spectroscopy Analysis

Metabolic analysis assays were done using an Ultra-performance liquid chromatography (UPLC), an Agilent 6,540 accurate–mass Q-TOF LC/MS (Agilent Technologies, United States). Liquid chromatographic separation was performed at room temperature of 18°C, using a UPLC C18 analytical column (kinetex C18 100 A Column, 4.6 × 50 mm, 2.6 micron). MS analyses were performed under the following operation parameters: dry gas temperature 350°C, dry gas (N2) flow rate 10 L/min, nebulizer pressure 30 psi, Vcap 4,000 and fragmentor voltage 120 V. Mass spectra were acquired in the positive ion mode as well as negative ion mode by scanning from 50 to 1,500 in the mass to charge ratio (m/z).

The assay was performed in 50 mM Tris-HCl buffer (pH- 8.0), 1.0 mM sodium ascorbate (pH- 7.0), 1.0 μg/ml of IDO enzyme protein and 1 mM of inhibitor 8d was added in to the deep well plate and incubate for 3 h at 37°C. After incubation, 100 µL of acetonitrile (HPLC grade) was added and the samples were centrifuged for 15 min at 13,000 rpm at 4°C in Amicon^®^ Ultra-15 centrifugal filter devices. The 200 µL of supernatant was then analyzed by Q-TOF LC/MS. For standardization of protocol same conditions was performed with 1.0 mM l-tryptophan to analyze the formation of N-formyl-kynurenine and N-kynurenine. The preparation of stock solution of inhibitor 8d was prepared in DMSO.

#### Drug Metabolism and Pharmacokinetics (DMPK Studies)

For the DMPK studies of the compound 8a-d we used rat as well as human plasmas. Rat plasma was purchased from Hylasco Biotechnology Pvt. Ltd.™ Hyderabad, India. We have not performed any *in vivo* experiments for this manuscript neither for animals nor for humans. Hence, we did not require any approval from the ethics committee. Also please note the blood plasma was obtained from the blood of Dr Srinivas R Maddi, who donated it and is the co-author of this article. The DMPK studies were supplemented by kinetic solubility, Log *p* and Log D determinations, PAMPA permeability, Metabolic and Plasma stability in rat and human, and finally the plasma folding in rat and human. A thorough discussion of each step is mentioned in the supplementary information.

## Computational Section

### Computational Setup

Since the crystal structure of the hIDO in presence of the inhibitors (8a–8e) were not available, we started with the Molecular Docking of the inhibitors 8a to 8e. The initial coordinates of the -IDO were imported from the protein data bank (PDB: 2DOT). The crystal structure contains 4-phenylimidazole as a bound ligand, therefore, we used the same binding site during docking of drugs 8a-e. During docking, we found the docking score of 8d as the best among all inhibitors. Therefore we chose the best pose of 8d for the further Molecular Dynamics simulations. Prior to MD simulations, all missing loops were modeled by MODELLER program, while we used Autodock Vina for all molecular docking calculations using UCSF Chimera (Pettersen et al., 2004).

During the MD simulations setup, the missing hydrogens and other heavy atoms were added by the leap module of the Amber20 program. For the protein residues we used Amber ff14SB forcefield while the parameters for the substrate was prepared using the antechamber module of the amber using Amber generalized forcefields for organic molecules (GAFF2). The partial charged of the substrate was calculated by the RESP charge fitting of a Quantum Mechanical (QM) optimized geometry at HF/6–31 g* level of theory (Bayly et al., 1993; Cornell et al., 1993). Parameters for the heme-bound histidine were prepared using the MCPB (Metal Center Parameter Builder Program) implemented in the Amber MD package. The pKa value of histidine, Aspartate and Glutamate were checked by propKa 3.0, and we found that the pKa of all these residues were well under 6.5. Therefore, these residues were kept at their default protonation state. However, Histidine residues were further checked for either as HIE (protonation at *e* position) or HID (protonation at δ position), depending upon the possible interactions with nearby polar group. A Na + ions was added into the protein surface to neutralize the total charge of the system. Finally, the resulting system was solvated in a rectangular box of TIP3P waters extending up to minimum cutoff of 10 Å from the protein boundary which resulted overall system size containing 47,531 atoms including protein, water and a Na + ion.

Following system setup and parametrization of the complex, the resulting geometries of the systems were minimized to remove the poor contacts and relax the proteins. The systems were then gently annealed from 10 to 300 K under the NVT ensemble for 50 ps? Subsequently, the systems were maintained for 1 ns in the NPT ensemble at a target temperature of 300 K and with the target pressure of 1.0 atm. using the Langevin thermostat (Jorgensen et al., 1983) and Berendsen barostat (Izaguirre et al., 2001) with collision frequency of 2 ps and pressure relaxation time of 1 ps. This was followed by further equilibrations for ∼3 ns for each system. Finally, a productive MD run was performed for 100 ns for the complex. The 100 ns simulation was performed using a multi-trajectory approach in which we performed two consecutive simulations each of 50 ns. At every 50 ns we restarted the simulation for the next 50 ns with a random velocity. During all MD simulations, the hydrogen bonds were constrained using SHAKE (Berendsen et al., 1984) and particle mesh Ewald (PME) (Ryckaert et al., 1977) was used to treat long-range electrostatic interactions. All MD simulations were performed with the GPU version of Amber 20 package (Darden et al., 1993).

### Quantum Mechanical/Molecular Mechanical Setup

The equilibrated snapshot from the MD simulations were used for the QM/MM calculations. All water molecules beyond 6Å of the protein were stripped from the MD snapshot. The QM region during the QM/MM calculations consists of truncated heme-porphyrin, O2, the imidazole group of proximal histidine, and the substrate. The atomic coordinates for the QM region can be found in the supplementary information (SI) document. The protein and water residues as close as 8 Å of the QM regions were treated as active atoms to account for their effect due to electrostatic and van der Waals interactions. All QM/MM calculations were performed using ChemShell (Sherwood et al., 2003; Salomon-Ferrer et al., 2013) combining Turbomole (Metz et al., 2014) for the QM part and DL_POLY (Ahlrichs et al., 1989) for the MM part using the Amber force field. The electronic embedding scheme (Smith and Forester, 1996) was used to account for the polarizing effect of the enzyme environment on the QM region. Hydrogen link atoms with the charge-shift model (Sherwood et al., 2003; Salomon-Ferrer et al., 2013) was applied to treat the QM−MM boundary. In QM/MM geometry optimizations, the QM region was treated using the hybrid UB3LYP functional with two basis sets, B1 and B2, where B1 stands for LACVP* and B2 is for def2-TZVP. For geometry optimization and frequency calculations, we used B1, i.e., LACVP basis set. All of the QM/MM transition states (TSs) were located by relaxed potential energy surface (PES) scans followed by full TS optimizations using the P-RFO optimizer implemented in the HDLC code. The energies were further corrected with the large all-electron def2-TZVP basis set. The zero-point energy (ZPE) was calculated for all species, and all of the final energies are reported as UB3LYP/B2+ZPE. The feasibility of these protocol has already been validated in several other studies by us in similar heme metalloenzymes (Dubey et al., 2017; Dubey and Shaik, 2018; Dubey and Shaik, 2019; Kalita et al., 2020). Previous studies (Capece et al., 2012; Shin et al., 2018) show Fe^+3^ and triplet state as the ground state for the hIDO mediated reactions, therefore we used the same oxidation and spin state in our calculations.

## Results and Discussion

### UV-Vis Absorption Study

**FIGURE 3:**
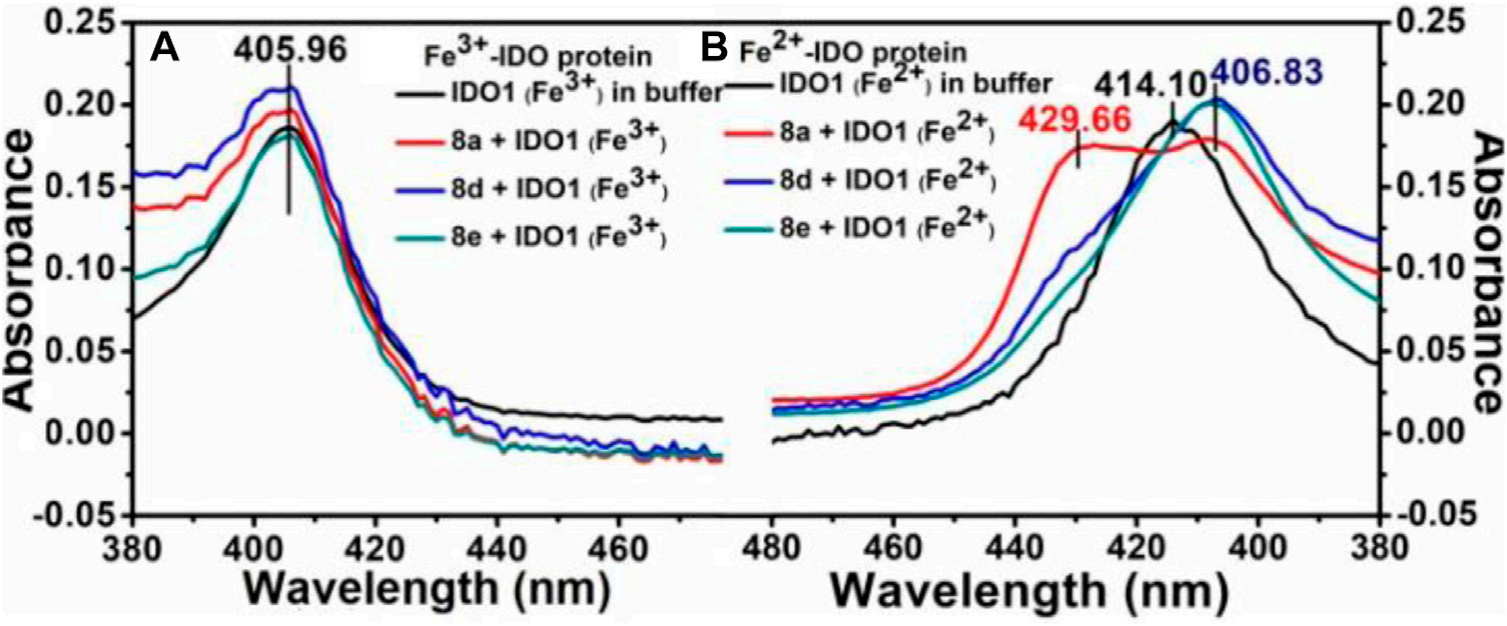
UV-Vis spectroscopic analyses of compounds **8a**, **d** and **e** with hIDO1, Experiment on enzyme kinetics to understand the mode of inhibition.

To unravel the mechanism by which our ligands interact with IDO1, we utilized the UV-Vis absorbance studies to understand the ligand-binding ability of compound **8d** with IDO1 enzyme. For better comparison the next best potent inhibitors compounds **8a** and **e** were also included. It is known that the heme group in the IDO1 enzyme undergoes change in optical properties such as optical rotation and absorption wavelength (in the UV-Vis region) based on the oxidation state of the metal of the heme part and on the nature of the ligands it is bound to (Soret, 1883). Our investigation correlates the IC_50_ values of inhibition of IDO1 enzyme by ligands **8a**, **d** and **e** with their binding ability at the active site of the enzyme. It further demonstrated that heme binding ligands like ours increases the stability of the enzyme under the experimental condition. Accordingly, the UV-Vis spectra of ferric-IDO1 and deoxy ferrous-IDO1 were measured in presence and absence of ligands **8a**, **d** and **e** (Figure 3 1) and b). In absence of the ligands the UV-VIS spectra of ferric-IDO1 in potassium phosphate buffer at pH - 6.5 exhibited a soret peak at 406 nm, which remained stable up to 3 h (Figure 3A). Interestingly, in presence of the ligands at 25 µM the soret peak of Fe^3+^-IDO1 remain unchanged at 406 nm with slight increase in intensity for **8a** and **8d** and decrease for **8e** (Figure 3A). This alteration of intensity of the soret band further suggest that the change in absorbance intensity of ligands with ferric-IDO1 (**8d** > **8a** > **8e**) can directly correlates with their inhibition potency (**8d** > **8a** > **8e**) (Figure 2) against IDO1 enzyme. In Figure 3B, the deoxy ferrous-IDO1 exhibited a soret peak at 414 nm. In the presence of compounds **8a**, **d** and **e** the soret band shifted to 407 nm which nearly coincided with the soret band of Fe^3+^-IDO1 (Figure 3A). This suggested that not only the ligands bound to the deoxy ferrous-IDO1 but also reacted with it to oxidize it back to the Fe^3+^ form. With compound **8a** the soret band also shifted to 430 nm, and according to reported literature this is typical a six coordinate low spin heme species complex bind with ligands (Figure 3B). (Terentis et al., 2002) Although proof of coordination of the compounds to the heme iron requires some further studies, these preliminary results strongly support the binding of all the three ligands **8a**, **d** and **e** to IDO1 (Figure 3).

**FIGURE 4:**
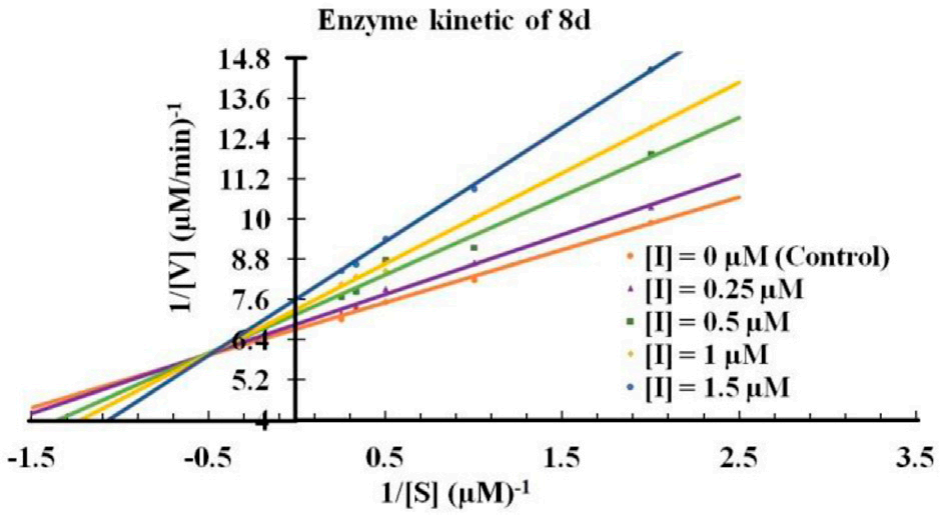
Lineweaver-Burke plot of compound **8d** against IDO enzyme. Molecular dynamics simulation of compound 8d with IDO1.

Next, It was important to understand the mode of inhibition of compound **8d** with IDO1. We performed enzyme kinetics experiments and obtained the Lineweaver-Burke plots (Figure 4). The plots of 1/V vs. 1/[S] divulged that **8d** inhibited IDO1 enzyme in a competitive manner (Figure 4). V and [S] depicted the initial rate of the reaction and the substrate concentration respectively. It is noteworthy, that the observed competitive mode of inhibition is with respect to IDO1. It may not be same for with respect to O_2_ (the other substrate in the L-Trp catabolism pathway). For this reason in order to determine the true mode of inhibition of IDO1 by **8d** additional kinetics measurements are required.

**FIGURE 5:**
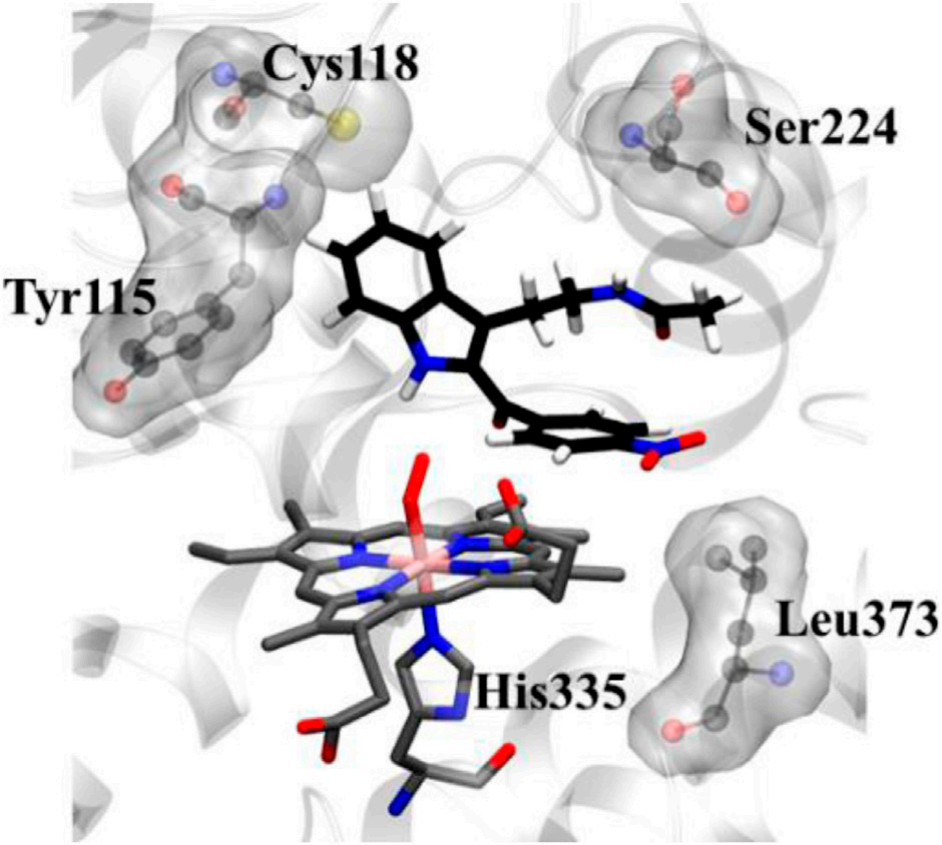
Key protein residues that forms the binding cleft for compound **8d**. The hydrogen atoms for the heme moiety are hidden for the sake of clarity. QM/MM calculations to investigate the IDO1 catalyzed degradation of compound 8d.

To get a microscopic insight of the substrate and enzyme interactions, we further enumerate the binding mode and crucial interactions between the inhibitor **8d** and IDO using molecular dynamics (MD) simulation in deoxy and resting state of heme. In both of the simulations the inhibitor **8d** stayed close to the catalytic center and occupied the axial position of heme prosthetic group. The binding stability of **8d** was mainly due to a stable stacking interaction with one of the pyrrole rings of heme-porphyrin and aromatic side chain of **8d**ays. A thorough inspection of MD trajectories showed that Tyr115, Ser224, and Leu373 surrounded the drug and provided an additional stability to the protein-drug complex (Figure 5). As can be seen from Figure 5, these residues form the active site architecture and provide a tight packing to the substrate via polar and hydrophobic interactions.

In addition, the indole ring of compound **8d** was also in the close contact with the Cys118 which may have act as a base for the catalytic reaction. A previous structural comparison of TDO with IDO indicated a Cysteine residue (C118) in IDO as a catalytic analogue of H55 of TDO (Uyttenhove et al., 2003). Incidentally, this C118 residue was far away from the catalytic center in the crystal structure of IDO-1 which had raised doubt on the role of Cysteine as a catalytic mechanism of IDO-1. However to our surprise, this Cys118 residue comes close to the substrate during the MD simulations which indicated that the presence of substrate may have instigated a local active site rearrangement. The role of Cysteine 118 could not be fully downplayed in the catalysis mechanism of IDO-1 and whether it really played a role in the catalysis is an open question (Figure 5). As classical MD simulations cannot describe the reaction mechanism, subsequently the QM/MM calculations were performed to study the mechanism of the catalysis as discussed in the next section.

**SCHEME 1:**
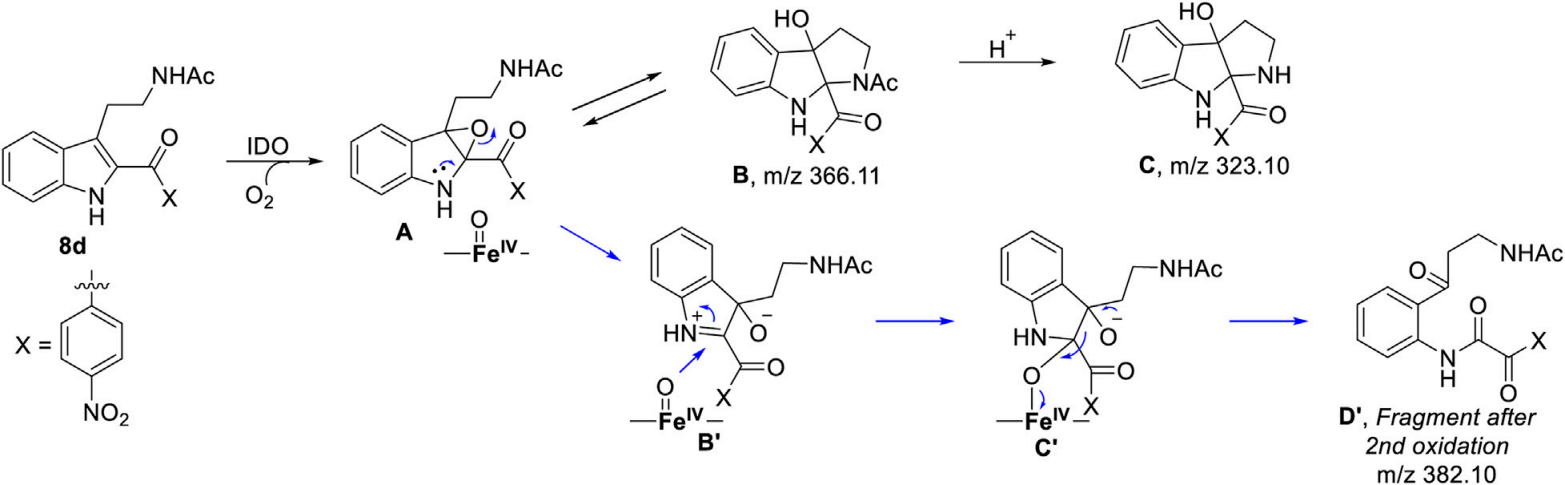
Probable mechanism of the degradation of compound **8d** based on UV-Vis spectroscopy, molecular dynamics simulation and QM/MM calculations.

**FIGURE 6:**
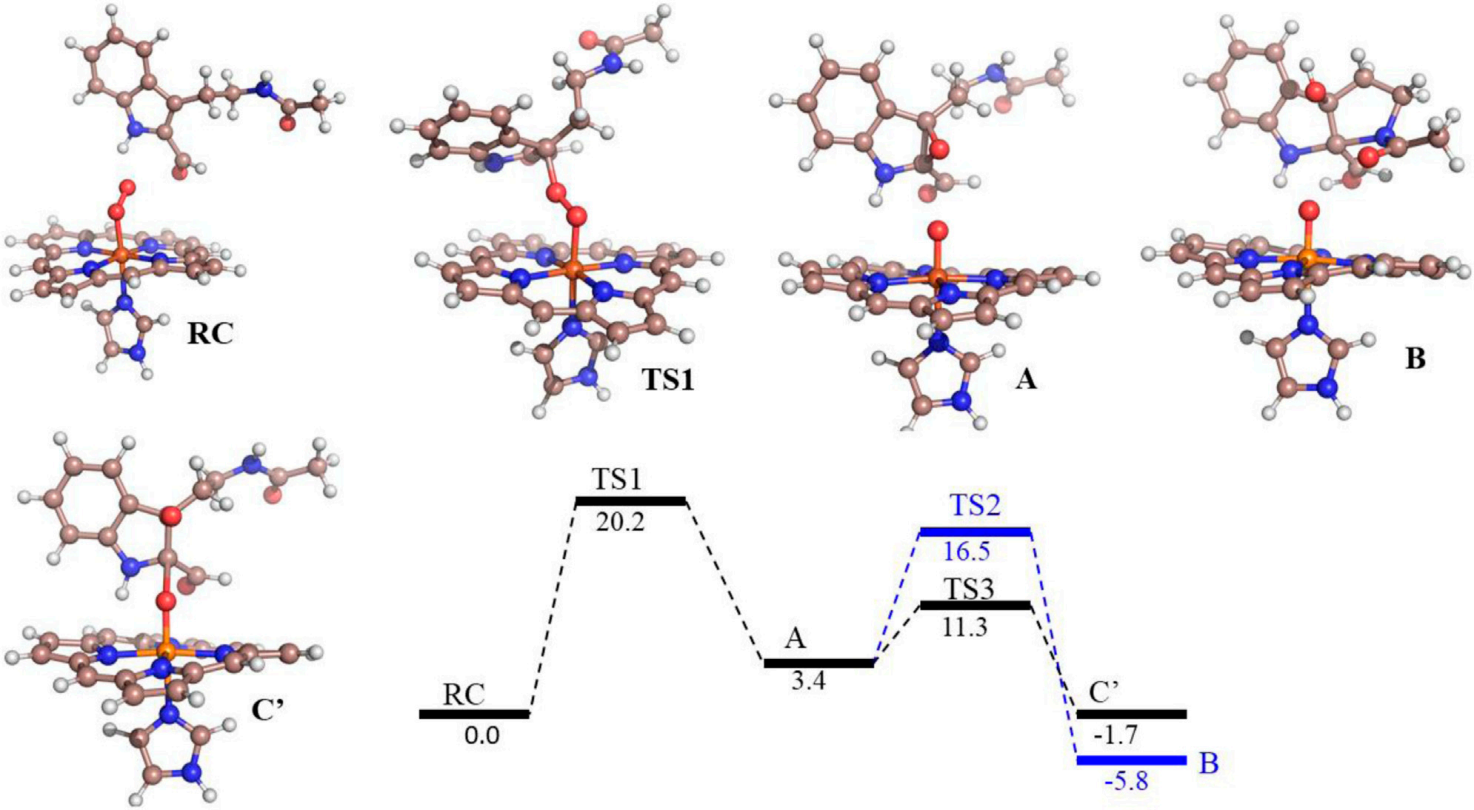
Potential energy profile of the reaction of compound 8d with hIDO1. Energies (in kcal/mol) are represented as QM(B1+ZPE)/MM followed by QM(B2+ZPE)/MM levels of theory where B1 is B3LYP-D3/LACVP* and B2 is B3LYP-D3/def2-TZVP Note that propionate group of heme, and benzoate group of substrate is truncated.

To understand the degradation of ligand **8d** with IDO1, the reaction mechanism (as depicted in Scheme 1) was modeled using hybrid QM/MM calculations (refer Materials and Methods) for a representative snapshot from the MD simulations (Figure 6). This reaction mechanism was based on the putative mechanism of the catabolism of l-tryptophan by the heme protein IDO1 as reported in the literature and from our initial UV-Vis absorption studies, which revealed the change in the oxidation state of the Fe moiety in IDO1 (Chauhan et al., 2009; Basran et al., 2011). The strategy involves enzyme dynamics at room temperature during the reaction while generating the quantum mechanical accuracy for the description of the active site atoms. This approach has been utilized successfully to model reaction mechanics in a diverse range of enzyme reaction.

To get the energy profile of the reaction, we started the PES scanning from 8d. The energy profile of this reaction along with the key intermediates are shown in Scheme 1/Figure 6. As it can be observed that, the reaction could begin with the conversion of compound **8d** (Figure 6), to the intermediate **A** (Figure 6) *via* a transition state **TS1** (Figure 6). The energy barrier of 20.2 kcal/mol, for this step is significantly high and therefore could be the rate determining process. The calculations indicated that the formation of **A** is endothermic and can easily transform to **B** or **C′** through low transition state barriers of 16.5 kcal/mol and 11.3 kcal/mol respectively. Therefore, the oxygen insertion from dioxyheme could be a stepwise process composed of two stages. At the initial stage, the reaction could have started with the bridging of molecular oxygen with the inhibitor **8d** which formed an epoxy indole intermediate at the cost of high energy. In the second step this epoxy-indole intermediate underwent the second oxygen insertion to ultimately afford the product. Surprisingly, as mentioned in previous section, we could not identify the involvement of cysteine residue as a possible base in the catalytic machinery. In summary, the theoretical calculations, predicted that the catalysis could have proceeded in a multistep sequence **via** different intermediates **A**, **B** and **C′**, where the epoxidation could be the rate determining step (Figure 6) (Sugimoto et al., 2006).

Mass spectroscopy studies of the metabolism of compound 8d by IDO1.

**FIGURE 7:**
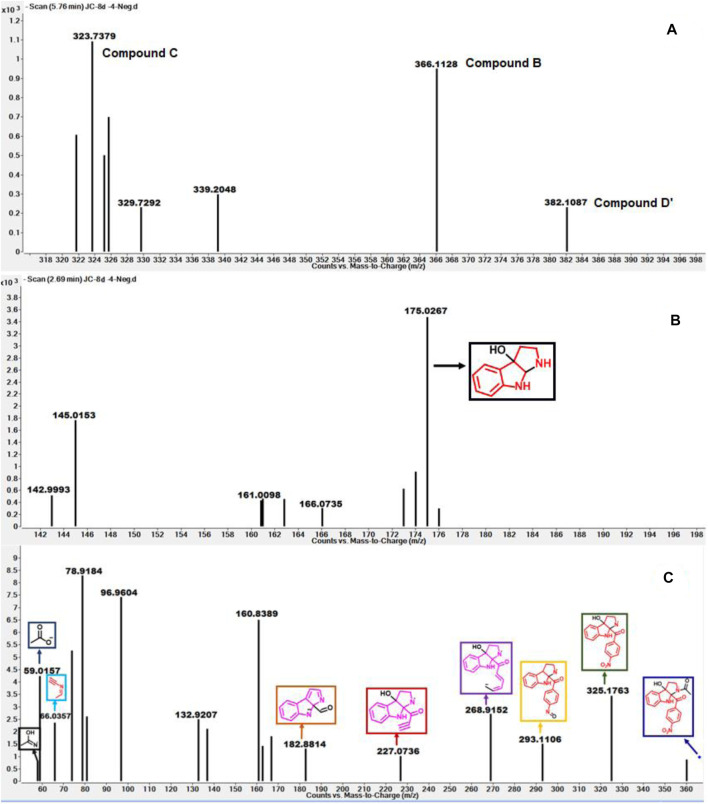
**(A)** The mass analysis of reaction of compound **8d** with IDO1 through QTof LC-MS; **(B)** LC-MS of **8d** with IDO1 at R_t_ = 2.69°min, **(C)** Fragmentation pattern via LC-MS/MS of compound **B** in negative mode.

As depicted in Figure 6 above, we understand that compound **8d** and l-tryptophan could initiate a competition between them for binding to the active site of IDO1 enzyme (deemed by QM/MM calculations. This encouraged us to corroborate our the predicted mechanism of the catabolism of **8d** with IDO1 *via* mass spectroscopic analysis (LC-MS). Accordingly IDO1 was incubated with the ligand in tris-HCl buffer (pH 8.0) and ascorbic acid at 37°C for 3 h (refer method and material section). An aliquot of the reaction mixture was then analyzed through QTOF LC-MS. In the LC-MS analysis a peak eluting with m/z 382 was observed in addition to the peaks with m/z 366 and 323. The peak with m/z 366 is consistent with insertion of a single oxygen atom into the substrate (m/z 8d = 350.11) (refer Figure 7). Using selected ion monitoring, the elution profile revealed a peak with m/z 175 (R_T_ 2.69 min) (refer Figure 7) which could have been generated by the loss of C2 aroyl moiety from intermediate **C** (refer Scheme 1 and Figure 7). We further speculated that the peak with m/z 382 corroborates with the formation of **D′**, the dioxygenated ring opened analogue of N-formyl kynuramine (NFK) (refer Scheme 1 and Figure 7). We further anticipated that the peak with m/z 366 may have originated from the initial transformation of compound **8d** to the 2, 3-epoxide derivative **A** (as the rate determining step by QM/MM calculations [Figure 6]) which could have immediately converted to the cyclic amino acetal intermediate **B** (same m/z as the epoxide A). The formation of **B** could be further established either from the peak with m/z 323 (refer Scheme 1 and Figure 7) which could be either assigned to the deacetylated intermediate **C** or by the MS-MS of intermediate **B** (refer Figure 7) (corroborated by MS-MS).

Hence this detailed analysis of the mechanism of metabolization of compound **8d** by IDO1 highlighted that during this reaction driven by oxygen, intermediate **A** was formed *via* insertion of a single oxygen atom into the compound **8d** (Scheme 1). Mechanistically it emphasized that the initial attack by the ferrous superoxide intermediate from IDO1 (formed as the primary requirement of the process) at C2 of **8d** afforded the epoxide **A**. Furthermore competitive reactions on **A** happened which included 1) an intramolecular cyclization of the N-acetylamine to open up the epoxide ring to provide **C** and 2) second oxidation by IDO-Fe(IV)-oxide to afford the ring opened N1-acyl-N2-acetylkynuramine derivative **D′** (Scheme 1). This investigation clarifies the degradation pathway by which indole based ligands metabolizes in presence of IDO1. This information will not only help to modify the lead compound **8d** into an appropriate preclinical candidate but will also guide to design appropriate new indole based lead molecules. It is noteworthy that the literature reports till date indicated that the reaction always occurred in an intermolecular fashion where the oxidized enzyme played the key role in the metabolism of l-tryptophan (the substrate) (Chauhan et al., 2009; Basran et al., 2011; Röhrig et al., 2019). It is the delivery of the oxygen atom from the oxidized enzyme that initiates the reaction at the C2-C3 position of the indole nucleus of the substrate and for our case, the ligand **8d** (Scheme 1). Ultimate cleavage of the C2-C3 bond to afford metabolite **D′** or intramolecular cyclization of the tethered amide to afford metabolite **C** was prompted by the transfer of oxygen atom from the oxidized heme to the ligand/substrate (Scheme 1).

### Drug Metabolism/Pharmacokinetics Studies of Compounds 8a, d, and e

Understanding the drug metabolism and pharmacokinetics (DMPK) properties of molecules is critical to decide its ability to become drug candidates. Hence the kinetic solubility, partition/distribution coefficient and PAMPA permeability of compounds **8a**, **d** and **e** were measured (Table 1). The experiments demonstrated that **8a**, **d**, and **e** showed very good solubility and permeability. The data in Table 1 indicated that these compounds may be amenable toward oral absorption in vivo pharmacokinetic studies. The partition and distribution studies of these compounds revealed a reasonable balance between hydrophilic and lipophilic property. Metabolic stability in different liver microsomes is presented in Table 2. **8a** was unstable and showed high clearance, where as **8d** and **8e** were found to be moderately stable. They showed 33 and 58% hepatic extraction ratio which is 30–70% of human hepatic blood flow. The animal microsomal data among rat, dog and monkey indicated high clearance for compound **8a** and low to moderate clearance for **8d** and **e**. Plasma stability data of compounds **8a**, **8d,** and **8e** in human plasma is also presented in Table 2. More than 85% of all the three compounds were retained in the plasma even after 120 min, which meant they are stable in the plasma. The rat and human plasma protein binding is presented in Table 2. Compound **8a** binds highly to human plasma followed by rat plasma with fraction unbound of 0.64, 0.49 and 2.7 and 1.6 respectively. On the contrary, compounds **8d** and **8e** exhibited moderate binding in human and rat plasma with fraction unbound of 9.5, 2.1 and 17.9 and 4.1 respectively. The stability and recovery of all three compounds in plasma was good across the species tested. Overall the DMPK properties of compound **8d** make it the most compatible for a lead candidate.

**TABLE 1:** Drug Metabolism and Pharmacokinetic (DMPK) Studies of compounds 8a, d and e.

S.No	Code of the compounds	Kinetic solubility of the compounds(μg/ml)^a^	Log D^a^	Log *p*	PAMPA (10^−6^cm/sec)^a^
1	**8a**	64.1	1.32	>3.5	6.8
2	**8d**	56.9	1.51	2.67	15.4
3	**8e**	67.4	2.95	2.88	32.3

a Kinetic solubility, Log D and PAMPA data of compounds at 1 mg/ml in phosphate buffer at pH 7.4.

Log D: Distribution coefficient; Log p: Indicates permeability of the drugs; PAMPA: indicates passive, trans-cellular permeability of drugs.

**TABLE 2:** Metabolic stability of compound 8a, d, and e in rat, dog, monkey and human liver microsomes.^a^

S. No	Human plasma stability	Human plasma protein binding		Human liver microsomal	Rat plasma stability	Rat plasma protein binding		Rat liver microsomal stability	Dog liver microsomal stability	Monkey liver microsomal stability
	%Ge remaining after 120 min	%Ge bound	%Ge free	% QH (hepatic extraction ratio)	%Ge remaining after 120 min	%Ge bound	%Ge free	% QH (hepatic extraction ratio)	% QH (hepatic extraction ratio)	% QH (hepatic extraction ratio)
**8a**	84.5	99.36	0.64	73.9	84.5	97.3	2.7	12.6	69	97
**8d**	85.0	90.48	9.52	32.6	91.3	82.1	17.9	2.2	39	74
**8e**	108.3	97.87	2.13	58.0	96.3	95.9	4.1	14.9	67	96

a Plasma protein binding data of compounds at 10 μM in liver microsomes of human, dog, monkey, and rats.

## Conclusion

Here in we have investigated the mechanism of inhibition of C2-aroyl indoles, a novel class of molecules against IDO1 through UV-Vis spectroscopy, MD simulation and QM/MM calculations. Ligand **8d** inhibits IDO1 substantially with an IC_50_ of 180 nM. UV absorption spectroscopy demonstrated that under the erobic reaction condition the heme binding ligands like **8a**, **d** and **e** stabilizes the ferric IDO1 more than the deoxy ferrous IDO1. Based on this and from the literature reports of l-tryptophan degradation by IDO a probable reaction mechanism was proposed that involved epoxidation of **8d** by IDO1 in presence of single oxygen, leading to either an ultimate ring opening of the indole moiety to provide the *o*-aminoacetophenone derivative **D′** or intramolecular cyclization of the exocyclic amine to afford the hexahydropyrrolo [2, 3-b]indole **E**. The MD simulations and molecular docking calculations rationalized the strong binding of the inhibitor **8d**, while the QM/MM calculations supported the putative mechanism of this reactions. This was supported by mass spectroscopic analysis of the reaction mixture over time. The reaction aliquots were analyzed to identify their structure and corroborate the predicted mechanism. The advantage of indole based ligands as inhibitors are their established legacy as IDO1 antagonist. However the collateral disadvantage is the competition that the ligand may face from l-tryptophan which is the original substrate of the enzyme. Through the computational calculations and experimental analysis we have demonstrated that ligand **8d** (structurally similar to tryptophan the original substrate of IDO1) is a suitable lead molecule with appropriate DMPK properties that can be developed into a preclinical candidate. This is presently ongoing in our lab.

## Data Availability

The original contributions presented in the study are included in the article/Supplementary Material, further inquiries can be directed to the corresponding authors.
