# Supplementary material for: Developing C2-Aroyl Indoles as Novel Inhibitors of IDO1 and Understanding Their Mechanism of Inhibition via Mass Spectroscopy, QM/MM Calculations and Molecular Dynamics Simulation
Source: Front Chem. 2021 Jul 15;9:691319. doi: 10.3389/fchem.2021.691319 (PMC8319603; doi:10.3389/fchem.2021.691319)
Supplement: Supplementary file 1 [file DataSheet1.PDF]

Developing C2-aroyl indoles as novel inhibitors of hIDO1 and understanding their mechanism of inhibition *via* mass spectroscopy, QM/MM calculations and molecular dynamics simulation

Jyoti Chauhan,[a] Srinivas R Maddi, [b] and Kshatresh Dutta Dubey* [a] Subhabrata Sen,*[a]

[a.]Department of Chemistry, School of Natural Sciences. Shiv Nadar University, Dadri, Chithera, Gautambudh Nagar, Uttar Pradesh 201314, India

[b.]Acubiosys PVT LTD, TBI, BITS-Pilani Campus, Jawahar Nagar, Hyderabad, Telangana, 500078, India.

Table of Content

1.	Figure S1. Percentage inhibition of the inhouse inventory of compounds against hIDO at 5 μM concentration	Pg 2
2	Figure S2. Molecular library of C2-aroyl indole derivatives 8a - p	Pg 3
3.	Figure S3.(a) The mass analysis of reaction of L-Trp with hIDO1 through Q-Tof LC-MS, b) LC-MS of L-trp without hIDO1	Pg 4
4	Details of chemical synthesis, biological and DMPK experiments	Pg, 5-14
5	Coordinates of QM region	Pg. 15
6	Figure S4. Root Mean Square Deviation during MD simulations	Pg. 16
7	1H and 13C Spectra	Pg. 17 to 48
8	Acquisition methods in LC-MS	Pg. 49 to 58
9	Figure S5. QM geometry showing link atom	Pg.16
10	Figure S6. Electronic structure of Reactant complex	Pg.17

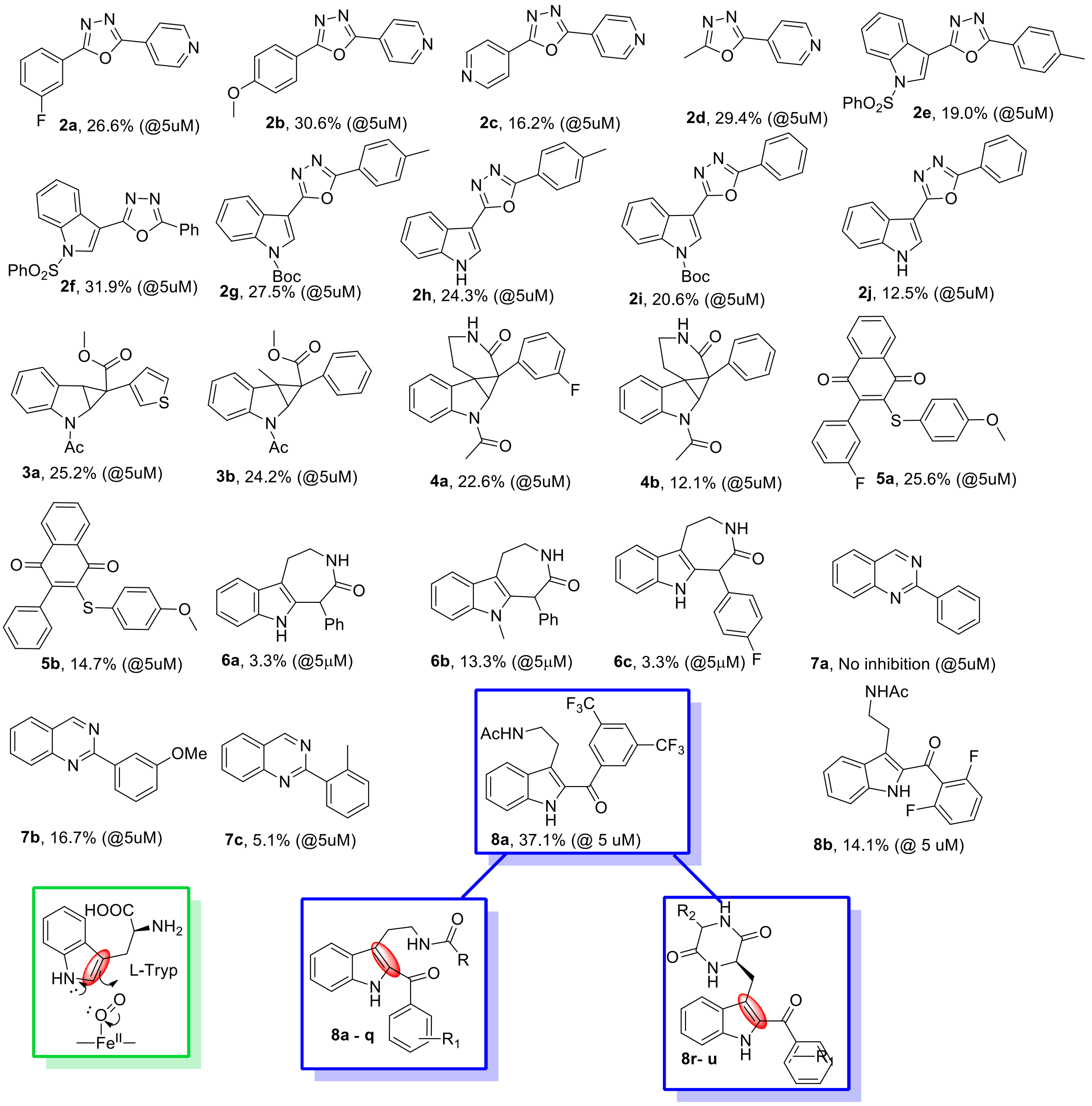

Figure S1. Percentage inhibition of the inhouse inventory of compounds against hIDO at 5 μM concentration

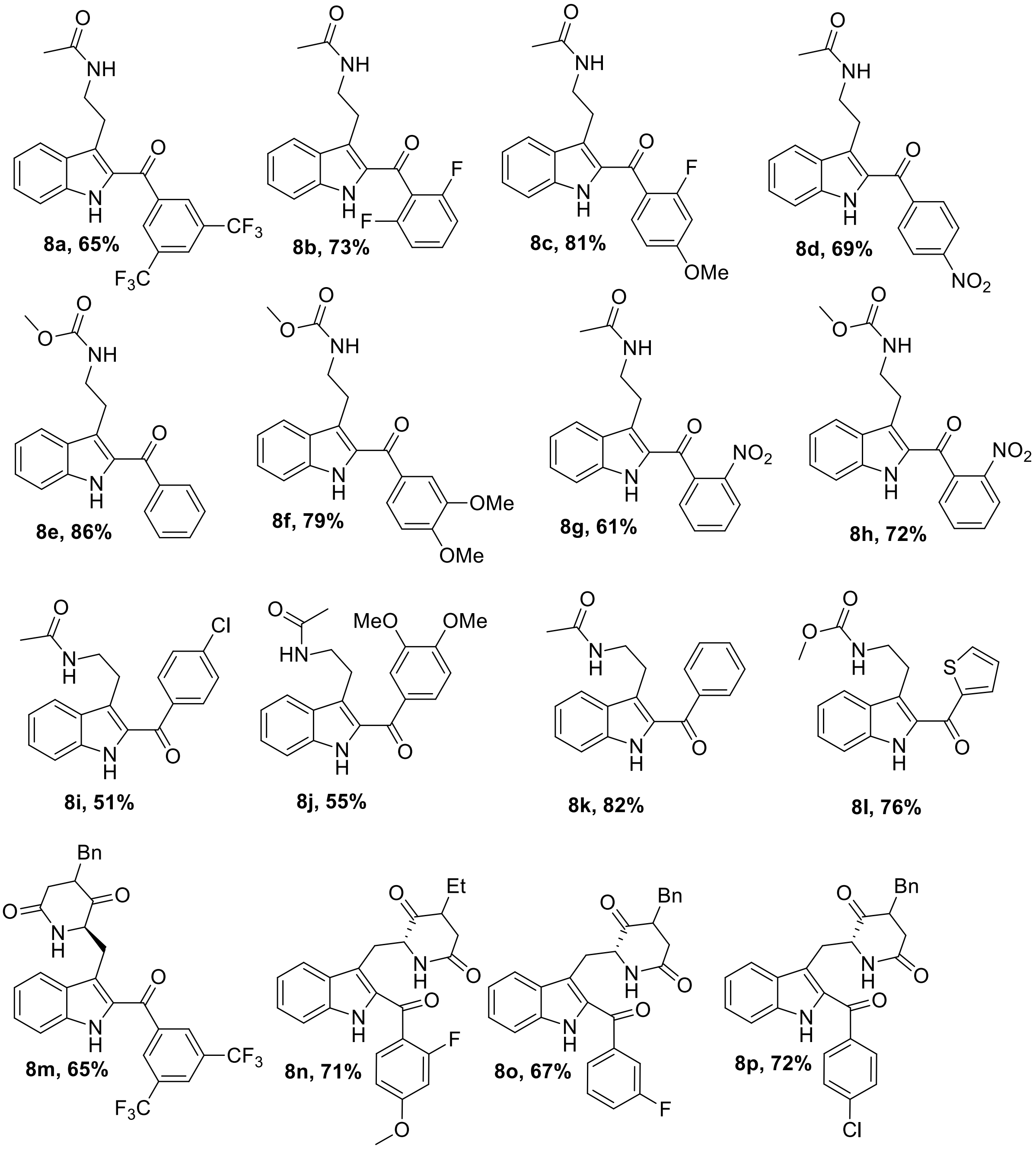

Figure S2. Molecular library of C2-aroyl indole derivatives **8a** - **p**

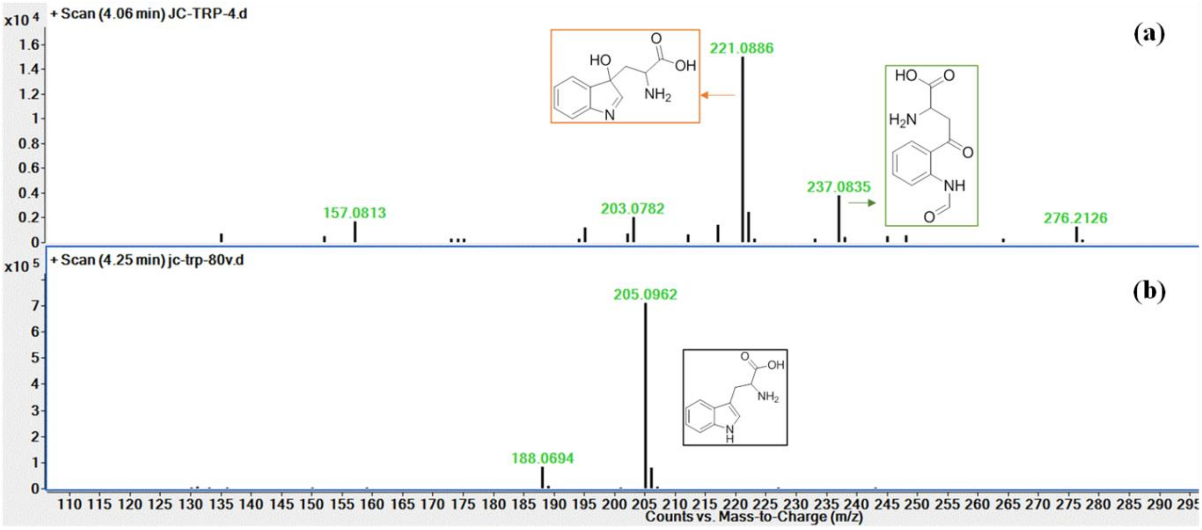

Figure.S3.(a) The mass analysis of control reaction of L-Trp with hIDO1 through Q-Tof LC-MS, b) LC-MS of L-trp without hIDO1.

Synthesis of Compounds and experimental details:

Preparation of **9a** - **n** and **10a** - **d**.

To the solution of tryptamine or L-tryptophan methyl ester (1 equiv.) in [bbim]BF_4 (5 mL) was added substituted benzaldehydes (0.8 equiv). The resulting mixture was allowed to stirred at 100 °C for 24 h. Once thin layer chromatography (TLC) confirmed complete consumption of the starting material, the reaction mixture was extracted with ether and the organic layer was separated, washed with water, brine and dried over anhydrous magnesium sulfate and the solvent was concentrated under reduced pressure. The 1-aryl tetrahydrocarbolines (1-arylTHC) **6a** - **r** were obtained by recrystallization with minimum amount of ethanol and was taken to the next step without further purification.

To a solution of **6a** - **n** (1 equiv.) (obtained from tryptamine derivatives) in 4N aq. sodium hydroxide (NaOH) solution (1 equiv.) or triethylamine (3 equiv.) in DCM was added drop wise methyl chloroformate (1 equiv.) or acetyl chloride (2 equiv.) at 0 °C. The reaction mixture was stirred at room temperature under nitrogen atmosphere for 3 h. Once TLC confirms the complete consumption of the starting material the reaction mixture was diluted with water and extracted with ethyl acetate. The organic layer was washed with saturated solution of sodium bicarbonate ($NaHCO_3$) and brine. The organic layer was dried over anhydrous sodium sulphate (Na_2SO_4), and evaporated under reduced pressure to obtained crude **9a** - **n**, which was pure enough to be used for green oxidation without purification.

Similarly to the solution of 1-arylTHCs **6o** - **r (**1equiv.) (obtained from tryptophan methyl ester), sodium bicarbonate (2.4 equiv.) and chloroacetyl chloride (1.2 equiv.) were stirred in dichloromethane at r. t. Once the reaction is over it is quenched with water and extracted with dichloromethane. The organic layer was separated, dried and evaporated to provide the crude intermediate. It was then treated with benzyl amine (5 equiv.) in dioxane and stirred at 60 °C for 24 h. The reaction was monitored by TLC. Once the reaction is complete the reaction mixture was poured in ice cold water and the product was precipitated. The crude product **10a** - **d** (obtained as diasteromeric mixture of 7:3), was filtered under suction and dried under reduced pressure which in the green oxidation step without purification.

N-(2-(2-(3,5-bis(trifluoromethyl)benzoyl)-1H-indol-3-yl)ethyl)acetamide (**8a**). Prepared according to general procedure from **7a** (tryptamine (500.00 mg, 3.12 mmol) and 3,5-bis(trifluoromethyl)benzaldehyde (0.41 ml, 2.49 mmol) and protected with acetyl group) and was purified with column chromatography with ethyl acetate-hexane (3:7) as eluent. It was generated as white solid with yield of 65%. ^{1}H NMR (400 MHz; DMSO): δ 11.58 (s, 1H), 8.45 (s, 1H), 8.36 (s, 2H), 7.86 (d, J= 4.0 Hz, 1H), 7.78 (d, J= 8.0 Hz, 1H), 7.44 (d, J= 8.0 Hz, 1H), 7.34 (t, J= 8.0 Hz, 1H), 7.14 (t, J= 8.0

Hz, 1H), 3.25 (dd, J= 4.0, 12.0 Hz, 2H), 3.06 (t, J = 8.0 Hz, 2H), 1.65 (s, 3H). ^{13}C NMR (100 MHz; DMSO): δ 185.6, 169.0, 141.2, 137.3, 130.7, 130.4, 129.5, 127.6, 126.1, 124.5, 122.9, 120.9, 120.2, 112.9, 24.8, 22.5. IR (neat, ν cm^{-1}): IR (neat, ν cm^{-1}): 3269, 2939, 1719, 1689, 1633, 1342. HRMS (EI+) m/z calcd. for $C_{21}H_{16}F_6N_2O_2$ $[M+H]^+$ 443.1189, found 443.1193. M.P. 154 °C. The purity (98.7%) was determined by HPLC on a stationary phase [Agilent Eclipse Plus C18 column, pump mode used Gradient with A (ACN) and B (0.1 % formic acid in water) as solvent system for 11 minutes), t_R = 7.498 min].

N-(2-(2-(2,6-difluorobenzoyl)-1H-indol-3-yl)ethyl)acetamide (**8b**). Prepared according to general procedure from **7b** (tryptamine (500.00 g, 3.12 mmol) and 2,6-difluorobenzaldehyde (0.26 ml, 2.49 mmol) and protected with acetyl group) and was purified by column chromatography with ethyl acetate-hexane (1:1) as eluent. It was generated as yellow solid with yield of 73%. ^{1}H NMR (400 MHz, DMSO): δ 11.68 (s, 1H), 7.91 (t, J = 4.0 Hz, 1H), 7.75 (d, J = 8.0 Hz, 1H), 7.73-7.65 (m, 1H), 7.42 (d, J = 8.0 Hz, 1H), 7.36-7.29 (m, 3H), 7.12 (t, J = 4.0 Hz, 1H), 3.16 (dd, J = 8.0, 12.0 Hz, 2H), 2.91 (t, J = 8.0 Hz, 2H), 2.56 (s, 3H). ^{13}C NMR (100 MHz, DMSO): δ 179.5, 169.1, 137.5, 132.9, 131.6, 127.8, 126.9, 122.4, 121.4, 120.3, 112.9, 112.7, 112.4, 29.5, 24.8, 22.6. IR (neat, ν cm^{-1}): 3316, 2922, 1771, 1689, 1465, 1189, 1002. HRMS (EI+) m/z calcd. for $C_{19}H_{16}F_2N_2O_2$ $[M+H]^+$ 343.1253, found 343.1299. M.P- 112 ˚C. The purity (96.6%) was determined by HPLC on a stationary phase [Agilent Eclipse Plus C18 column, pump mode used Gradient with A (acetonitrile) and B (0.1 % formic acid in water) as solvent system for 11 minutes), t_R = 4.782 min].

N-(2-(2-(2-fluoro-4-methoxybenzoyl)-1H-indol-3-yl)ethyl)acetamide (**8c**). Prepared according to general procedure from **7c** (tryptamine (500.00 mg, 3.12 mmol) and 2-fluoro-4-methoxybenzaldehyde (0.32 ml, 2.49 mmol) and protected with acetyl group) and was purified by column chromatography with ethyl acetate-hexane (1:1) as eluent. It was generated as yellow solid with yield of 81%. ^{1}H NMR (400 MHz, DMSO): δ 11.47 (s, 1H), 7.90 (t, J = 8.0 Hz, 1H), 7.75 (d, J = 8.0 Hz, 1H), 7.55 (t, J = 8.0 Hz, 1H), 7.41 (d, J = 8.0 Hz, 1H), 7.29 (t, J = 8.0 Hz, 1H), 7.09 (t, J = 8.0 Hz, 1H), 7.01 (d, J = 12.0 Hz, 1H), 6.95 (t, J = 8.0 Hz, 1H), 3.88 (s, 3H), 3.21 (dd, J = 4.0, 12.0 Hz, 2H), 2.95 (t, J = 8.0 Hz, 2H), 1.72 (s, 3H). ^{13}C NMR (100 MHz, DMSO): δ 184.2, 169.0, 163.2, 162.0, 159.5, 136.8, 132.1, 131.6, 127.6, 125.6, 121.2, 120.9, 119.8, 112.8, 110.8, 102.3, 102.0, 56.1, 24.9, 22.6. IR (neat, ν cm^{-1}): 3310, 2933, 1649, 1629, 1189, 1161, 1094. HRMS (EI+) m/z calcd. for $C_{20}H_{19}FN_2O_3$ $[M+H]^+$ 355.1492, found 355.1492. M.P- 135 ˚C. The purity (94.3%) was determined by HPLC on a stationary phase [Agilent Eclipse Plus C18 column, pump mode used Gradient with A (acetonitrile) and B (0.1 % formic acid in water) as solvent system for 11 minutes), t_R = 4.897 min].

N-(2-(2-(4-nitrobenzoyl)-1H-indol-3-yl)ethyl)acetamide (**8d**). Prepared according to general procedure from **7d** (tryptamine (500.00 mg, 3.12 mmol) and 4-nitrobenzaldehyde (377.50 mg, 2.49 mmol) and

protected with acetyl group) and was purified by column chromatography with ethyl acetate-hexane (1:1) as eluent. It was generated as yellow solid with yield of 69%. ^{1}H NMR (400 MHz, DMSO): δ 11.55 (s, 1H), 8.41 (d, J = 8.0 Hz, 2H), 7.99 (d, J = 12.0 Hz, 2H), 7.78 (s, 1H), 7.76 (d, J = 8.0 Hz, 1H), 7.44 (d, J = 8.0 Hz, 1H), 7.33 (t, J = 8.0 Hz, 1H), 7.12 (t, J = 8.0 Hz, 1H), 3.22 (dd, J = 8.0, 12.0 Hz, 2H), 2.96 (t, J = 4.0 Hz, 2H), 1.69 (s, 3H). ^{13}C NMR (100 MHz, DMSO): δ 187.0, 168.9, 149.2, 144.6, 137.2, 130.9, 129.9, 127.6, 126.1, 123.8, 122.5, 121.0, 112.9, 25.1, 22.6. IR (neat, ν cm^{-1}): 3292, 3164, 2936, 1630, 1599, 1435, 1195, 1007. HRMS (EI+) m/z calcd. for $C_{19}H_{17}N_3O_4$ $[M+H]^+$ 352.1292, found 352.1295. M.P- 158 ˚C. The purity (99.9%) was determined by HPLC on a stationary phase [Agilent Eclipse Plus C18 column, pump mode used Gradient with A (acetonitrile) and B (0.1 % formic acid in water) as solvent system for 11 minutes), t_R = 5.111 min].

Methyl (2-(2-benzoyl-1H-indol-3-yl)ethyl)carbamate (**8e**). Prepared according to general procedure from **7e** (tryptamine (500.00 mg, 3.12 mmol) and benzaldehyde (0.25 ml, 2.49 mmol) and protected with carbomethoxy group) and was purified by column chromatography with ethyl acetate-hexane (3:7) as eluent. It was generated as pale yellow solid with yield of 86%. ^{1}H NMR (400 MHz; DMSO): δ 11.47 (s, 1H), 7.78 (s, 1H), 7.76 (s, 2H), 7.68 (t, J = 8.0 Hz, 1H), 7.58 (t, J = 8.0 Hz, 2H), 7.43 (t, J = 8.0 Hz, 1H), 7.29 (t, J = 8.0 Hz, 1H), 7.17 (t, J = 4.0 Hz, 1H), 7.11 (t, J = 8.0 Hz, 1H), 3.47 (s, 3H), 3.19 (dd, J = 8.0, 12.0 Hz, 2H), 3.01 (t, J = 8.0 Hz, 2H). ^{13}C NMR (100 MHz; DMSO): δ 188.5, 156.6, 138.9, 136.8, 132.3, 131.5, 128.6, 127.6, 125.3, 120.8, 120.6, 119.8, 112.8, 51.1, 41.4, 25.3. IR (neat, ν cm^{-1}): 3393, 3057, 2923, 2851, 1730, 1705, 1615, 1595, 1447, 1335. HRMS (EI+) m/z calcd. for $C_{19}H_{18}N_2O_3$ $[M+H]^+$ 323.1390, found 323.1398. M.P- 105 ˚C. The purity (87.00%) was determined by HPLC on a stationary phase [Agilent Eclipse Plus C18 column, pump mode used Gradient with A (acetonitrile) and B (0.1 % formic acid in water) as solvent system for 11 minutes), t_R = 6.180 min].

N-(2-(2-(3,4-dimethoxybenzoyl)-1H-indol-3-yl)ethyl)acetamide (**8f**). Prepared according to general procedure from **7f** (tryptamine (500.00 mg, 3.12 mmol) and 3,4-dimethoxybenzaldehyde (414.59 mg, 2.49 mmol) and protected with acetyl group) and was purified by column chromatography with ethyl acetate-hexane (1:1) as eluent. It was generated as yellow solid with yield of 79%. ^{1}H NMR (400 MHz, $CDCl_3$): δ 11.45 (s, 1H), 7.75 (d, J = 8.0 Hz, 1H), 7.45-7.38 (m, 4H), 7.27 (t, J = 8.0 Hz, 1H), 7.19 (t, J = 8.0 Hz, 1H), 7.13 (d, J = 8.0 Hz, 1H), 7.10 (t, J = 8.0 Hz, 1H), 3.88 (s, 3H), 3.83 (s, 3H), 3.47 (s, 1H), 3.21 (dd, J = 8.0, 12.0 Hz, 2H), 3.02 (t, J = 8.0 Hz, 2H). ^{13}C NMR (100 MHz, CDCl3): δ 187.2, 156.6, 152.6, 148.6, 136.5, 131.8, 131.1, 127.5, 124.9, 124.0, 120.4, 119.6, 112.7, 111.7, 55.8, 55.5, 51.1, 25.3. IR (neat, ν cm^{-1}): 3391, 2919, 2848, 1715, 1645, 1512, 1418, 1135, 1002. HRMS (EI+) m/z calcd. for $C_{21}H_{22}N_2O_4$ $[M]^-$ 365.1505, found 365.1507. M.P- 168 ˚C. The purity (93.4%) was determined by HPLC on a stationary phase [Agilent Eclipse Plus C18 column, pump mode used Gradient with A (acetonitrile) and B (0.1 % formic acid in water) as solvent system for 11 minutes), t_R = 4.193 min].

N-(2-(2-(2-nitrobenzoyl)-1H-indol-3-yl)ethyl)acetamide (**8g**). Prepared according to general procedure from **7g** (tryptamine (500.00 mg, 3.12 mmol) and 2-nitrobenzaldehyde (377.50 mg, 2.49 mmol) and protected with acetyl group) and was purified by column chromatography with ethyl acetate-hexane (1:1) as eluent. It was generated as yellow solid with yield of 61%. ^{1}H NMR (400 MHz, $CDCl_3$): δ 8.88 (s, 1H), 8.27 (d, J = 8.0 Hz, 1H), 7.82 (t, J = 8.0 Hz, 1H), 7.74-7.70 (m, 2H), 7.54 (d, J = 8.0 Hz, 1H), 7.39 (d, J = 4.0 Hz, 2H), 7.19-7.15 (m, 1H), 5.74 (s, 1H), 3.39 (dd, J = 8.0, 12.0 Hz, 2H), 2.91 (t, J = 4.0 Hz, 2H), 1.83 (s, 3H). ^{13}C NMR (100 MHz, $CDCl_3$): δ 185.4, 170.5, 146.7, 137.2, 136.1, 134.6, 131.2, 131.0, 128.6, 128.4, 127.7, 125.3, 123.5, 121.7, 112.5, 40.5, 24.7, 23.3. IR (neat, ν cm^{-1}): 3296, 3184, 2923, 1651, 1548, 1411, 1182, 1012. HRMS (EI+) m/z calcd. for $C_{19}H_{17}N_3O_4$ $[M+H]^+$ 352.1292, found 352.1295. M.P- 128 ˚C. The purity (96.30%) was determined by HPLC on a stationary phase [Agilent Eclipse Plus C18 column, pump mode used Gradient with A (acetonitrile) and B (0.1 % formic acid in water) as solvent system for 11 minutes), t_R = 4.811 min].

Methyl (2-(2-(2-nitrobenzoyl)-1H-indol-3-yl)ethyl)carbamate (**8h**). Prepared according to general procedure from **7h** (tryptamine (500.00 mg, 3.12 mmol) and 2-nitrobenzaldehyde (377.50 mg, 2.49 mmol) and protected with carbomethoxy group) and was purified by column chromatography with ethyl acetate-hexane (3:7) as eluent. It was generated as pale yellow solid with yield of 72%. ^{1}H NMR (400 MHz; DMSO): δ 11.53 (s, 1H), 8.31 (d, J = 8.0 Hz, 1H), 7.94 (t, J = 8.0 Hz, 1H), 7.87 (t, J = 8.0 Hz, 1H), 7.75-7.69 (m, 2H), 7.40 (d, J = 8.0 Hz, 1H), 7.32 (t, J = 8.0 Hz, 1H), 7.16 (t, J = 8.0 Hz, 1H), 7.11 (t, J = 8.0 Hz, 1H), 3.48 (s, 3H), 3.08 (dd, J = 4.0, 12.0 Hz, 2H), 2.83 (t, J = 8.0 Hz, 2H). ^{13}C NMR (100 MHz; DMSO): δ 185.3, 156.6, 146.0, 137.3, 135.9, 134.9, 131.3, 130.7, 128.8, 127.6, 126.3, 125.0, 120.9, 120.1, 112.8, 51.2, 41.1, 25.3. IR (neat, ν cm^{-1}): 3386, 3093, 2986, 2866, 1720, 1715, 1622, 1586, 1426, 1323. HRMS (EI+) m/z calcd. for $C_{19}H_{17}N_3O_4$ $[M+H]^+$ 352.1292. M.P- 115 ˚C. The purity (89.00%) was determined by HPLC on a stationary phase [Agilent Eclipse Plus C18 column, pump mode used Gradient with A (acetonitrile) and B (0.1 % formic acid in water) as solvent system for 11 minutes), t_R = 5.180 min].

N-(2-(2-(4-chlorobenzoyl)-1H-indol-3-yl)ethyl)acetamide (**8i**). Prepared according to general procedure from **7i** (tryptamine (500.00 mg, 3.12 mmol) and 4-chlorobenzaldehyde (0.31 ml, 2.49 mmol) and protected with acetyl group) and was purified by column chromatography with ethyl acetate-hexane (1:1) as eluent. It was generated as yellow solid with yield of 51%. ^{1}H NMR (400 MHz, DMSO): δ 11.69 (s, 1H), 7.97 (s, 1H), 7.94 (d, J = 8.0 Hz, 1H), 7.90 (t, J = 8.0 Hz, 1H), 7.79 (d, J = 8.0 Hz, 2H), 7.66 (d, J = 8.0 Hz, 2H), 7.60-7.56 (m, 1H), 7.40 (s, 2H), 3.22 (dd, J = 8.0, 12.0 Hz, 2H), 2.96 (t, J = 8.0 Hz, 2H), 1.68 (s, 3H). ^{13}C NMR (100 MHz, DMSO): δ 187.3, 169.0, 137.4, 137.2, 135.3, 132.2, 131.2, 130.9, 129.3, 128.8, 127.9, 123.0, 120.7, 114.9, 112.4, 24.8, 22.6. IR (neat, ν cm^{-1}): 3272, 3137, 2924, 1748, 1647, 1585, 1434, 1196, 1065. HRMS (EI+) m/z calcd. for $C_{19}H_{17}ClN_2O_2$ $[M]^-$ 339.0966, found

339.0906. M.P- 139 ˚C. The purity (99.9%) was determined by HPLC on a stationary phase [Agilent Eclipse Plus C18 column, pump mode used Gradient with A (acetonitrile) and B (0.1 % formic acid in water) as solvent system for 11 minutes), t_R = 5.775 min].

N-(2-(2-(3,4-dimethoxybenzoyl)-1H-indol-3-yl)ethyl)acetamide (**8j**). Prepared according to general procedure from **7j** (tryptamine (500.00 mg, 3.12 mmol) and 3,4-dimethoxybenzaldehyde (414.59 mg, 2.49 mmol) and protected with acetyl group) and was purified by column chromatography with ethyl acetate-hexane (1:1) as eluent. It was generated as yellow solid with yield of 55%. ^{1}H NMR (400 MHz, $CDCl_3$): δ 8.67 (s, 1H), 7.75 (d, J = 8.0 Hz, 1H), 7.49-7.36 (m, 4H), 7.20 (t, J = 8.0 Hz, 1H), 6.94 (d, J = 8.0 Hz, 1H), 6.90 (s, 2H), 3.97 (s, 3H), 3.95 (s, 3H), 3.57 (dd, J = 8.0, 12.0 Hz, 2H), 3.21 (t, J = 8.0 Hz, 2H), 1.89 (s, 3H). ^{13}C NMR (100 MHz, $CDCl_3$): δ 187.9, 170.5, 153.5, 149.5, 136.4, 132.1, 131.1, 127.9, 126.4, 122.3, 121.1, 112.2, 111.8, 110.4, 56.3, 56.3, 41.2, 24.2, 23.4. IR (neat, ν cm^{-1}): 3282, 3127, 2984, 1756, 1667, 1549, 1429, 1136, 1066. HRMS (EI+) m/z calcd. for $C_{21}H_{22}N_2O_4$ $[M]^+$ 367.1652, found 367.1656. M.P- 135 ˚C. The purity (95.9%) was determined by HPLC on a stationary phase [Agilent Eclipse Plus C18 column, pump mode used Gradient with A (acetonitrile) and B (0.1 % formic acid in water) as solvent system for 11 minutes), t_R = 5.111 min].

N-(2-(2-benzoyl-1H-indol-3-yl)ethyl)acetamide (**8k**). Prepared according to general procedure from **7k** (tryptamine (500.00 mg, 3.12 mmol) and benzaldehyde (0.25 ml, 2.49 mmol) and protected with acetyl group) and was purified by column chromatography with ethyl acetate-hexane (1:1) as eluent. It was generated as yellow solid with yield of 82%. ^{1}H NMR (400 MHz, CD_3OD): δ 7.82 (s, 1H), 7.76 (d, J = 8.0 Hz, 1H), 7.65 (t, J = 8.0 Hz, 1H), 7.56 (d, J = 8.0 Hz, 1H), 7.43 (d, J = 12.0 Hz, 1H), 7.31 (t, J = 8.0 Hz, 1H), 7.12 (t, J = 8.0 Hz, 1H), 3.43 (t, J = 8.0 Hz, 2H), 3.16 (t, J = 8.0 Hz, 2H), 1.81 (s, 3H). ^{13}C NMR (100 MHz, CD_3OD): δ 191.2, 173.2, 140.6, 138.6, 133.5, 130.3, 129.7, 129.3, 126.9, 123.2, 121.7, 21.2, 113.6, 41.7, 25.7, 22.5. IR (neat, ν cm^{-1}): 3289, 2918, 2849, 1731, 1636, 1526, 1378, 1176, 1020. HRMS (EI+) m/z calcd. for $C_{19}H_{18}N_2O_2$ $[M]^-$ 305.1299, found 305.1296. M.P- 102 ˚C. The purity (99.9%) was determined by HPLC on a stationary phase [Agilent Eclipse Plus C18 column, pump mode used Gradient with A (acetonitrile) and B (0.1 % formic acid in water) as solvent system for 11 minutes), t_R = 4.607 min].

Methyl (2-(2-(4-bromothiophene-2-carbonyl)-1H-indol-3-yl)ethyl)carbamate (**8l**). Prepared according to general procedure from **7l** (tryptamine (500.00 mg, 3.12 mmol), 4-bromo-2-thiophenecarboxaldehyde (476.66 mg, 2.49 mmol) and protected with carbomethoxy group) and was purified by column chromatography with ethyl acetate-hexane (1:1) as eluent. It was generated as yellow solid with yield of 76%. ^{1}H NMR (400 MHz, $CDCl_3$): δ 8.67 (s, 1H), 7.77 (d, J = 8.0 Hz, 1H), 7.69 (s, 1H), 7.63 (s, 1H), 7.47-7.39 (m, 2H), 7.22 (t, J = 8.0 Hz, 1H), 5.55 (s, 1H), 3.61 (s, 3H), 3.53 (t, J = 8.0 Hz, 2H), 3.27 (t, J = 8.0 Hz, 2H): ^{13}C NMR (100 MHz, $CDCl_3$): δ 157.3, 144.6, 136.9, 135.2, 131.2, 128.2,

127.1, 124.0, 121.5, 121.3, 112.5, 111.0, 52.1, 42.1, 24.9. IR (neat, ν cm^{-1}): 3225, 3114, 2916, 2849, 1720, 1695, 1138, 1017. HRMS (EI+) m/z calcd. for $C_{17}H_{15}BrN_2O_3S$ $[M+H]^+$ 407.0060, found 407.0091. M.P- 130 ˚C. The purity (97.81%) was determined by HPLC on a stationary phase [Agilent Eclipse Plus C18 column, pump mode used Gradient with A (acetonitrile) and B (0.1 % formic acid in water) as solvent system for 11 minutes), t_R = 7.459 min].

1-benzyl-3-((2-(3,5-bis(trifluoromethyl)benzoyl)-1H-indol-3-yl)methyl)piperazine-2,5-dione (**8m**).

Prepared according to general procedure from **6a** (tryptophan methyl ester (500.00 mg, 2.29 mmol) and 3, 5-bis(trifluoromethyl)benzaldehyde (0.41 ml, 2.49 mmol) and protected with N-benzyl diketopiperzine group) and was purified by column chromatography with ethyl acetate-hexane (3:7) as eluent. It was generated as yellow solid with yield of 63%. ^1H NMR (400 MHz, DMSO): δ 11.70 (s, 1H), 8.42 (s, 1H), 8.31 (s, 1H), 8.05 (s, 1H), 7.75 (d, J = 8.0 Hz, 1H), 7.48 (d, J = 8.0 Hz, 1H), 7.36 (t, J = 8.0 Hz, 1H), 7.27 (d, J = 8.0 Hz, 1H), 7.15 (t, J = 8.0 Hz, 1H), 7.03 (t, J = 8.0 Hz, 1H), 4.43 (d, J = 12.0 Hz, 1H), 4.40 (d, J = 12.0 Hz, 1H), 4.20 (d, J = 12.0 Hz, 1H), 4.15-4.12 (m, 1H), 3.56 (dd, J = 4.0, 12.0 Hz, 1H), 3.50-3.42 (m, 1H), 3.24 (d, J = 16.0 Hz, 1H). ^{13}C NMR (100 MHz, DMSO): δ 185.9, 165.8, 164.7, 140.9, 137.0, 135.9, 131.6, 130.6, 130.3, 129.5, 128.5, 127.6, 127.5, 125.9, 125.3, 124.4, 121.7, 121.0, 118.5, 112.9, 55.5, 48.4, 48.2, 28.9. IR (neat, ν cm^{-1}): 3421, 3233, 2928, 1676, 1579, 1425, 1313, 1262, 1187, 972. HRMS (EI+) m/z calcd. for $C_{29}H_{21}F_6N_3O_3$ $[M+H]^+$ 574.1560, found 574.1559. M.P- 120 ˚C. The purity (86.21%) was determined by HPLC on a stationary phase [Agilent Eclipse Plus C18 column, pump mode used Gradient with A (acetonitrile) and B (0.1 % formic acid in water) as solvent system for 11 minutes), t_R = 6.136 min].

1-ethyl-3-((2-(2-fluoro-4-methoxybenzoyl)-1H-indol-3-yl)methyl)piperazine-2,5-dione (**8n**).

Prepared according to general procedure from **6b** (tryptophan methyl ester (500.00 mg, 2.29 mmol) and 2-fluoro-4-methoxybenzaldehyde (0.32 ml, 2.49 mmol) and protected with N-ethyl diketopiperzine group) and was purified by column chromatography with ethyl acetate-hexane (3:7) as eluent. It was generated as yellow solid with yield of 76%. ^1H NMR (400 MHz, DMSO): δ 11.49 (s, 1H), 7.91 (s, 1H), 7.66 (d, J = 8.0 Hz, 1H), 7.53 (t, J = 8.0 Hz, 1H), 7.41 (d, J = 8.0 Hz, 1H), 7.27 (t, J = 8.0 Hz, 1H), 7.07 (t, J = 8.0 Hz, 1H), 7.00-6.93 (m, 2H), 4.05-4.02 (m, 1H), 3.88 (s, 3H), 3.52-3.47 (m, 1H), 3.43-3.34 (m, 2H), 3.19 (d, J = 20.0 Hz, 1H), 3.13-3.02 (m, 2H), 0.75 (t, J = 8.0 Hz, 3H). ^{13}C NMR (100 MHz, DMSO): δ 184.1, 165.2, 164.9, 136.5, 132.9, 132.1, 127.5, 125.3, 120.9, 119.8, 116.9, 112.6, 110.6, 101.9, 56.0, 55.8, 48.1, 28.9, 11.2. IR (neat, ν cm^{-1}): 3412, 3306, 2894, 1676, 1563, 1438, 1321, 1284, 1197, 968. HRMS (EI+) m/z calcd. for $C_{23}H_{22}FN_3O_4$ $[M+H]^+$ 424.1667, found 424.1653 M.P- 116 ˚C. The purity (92.26%) was determined by HPLC on a stationary phase [Agilent Eclipse Plus C18

column, pump mode used Gradient with A (acetonitrile) and B (0.1 % formic acid in water) as solvent system for 11 minutes), t_R = 6.896 min].

1-benzyl-3-((2-(3-fluorobenzoyl)-1H-indol-3-yl)methyl)piperazine-2,5-dione (**8o**). Prepared according to general procedure from **6c** (tryptophan methyl ester (500.00 mg, 2.29 mmol) and 3-fluorobenzaldehyde (0.19 ml, 1.83 mmol)) and was purified by column chromatography with ethyl acetate-hexane (3:7) as eluent. It was generated as yellow solid with yield of 83%. ^{1}H NMR (400 MHz, DMSO): δ 11.59 (s, 1H), 8.05 (d, J = 4.0 Hz, 1H), 7.72 (d, J = 8.0 Hz, 1H), 7.60-7.55 (m, 1H), 7.49 (t, J = 8.0 Hz, 3H), 7.33 (t, J = 8.0 Hz, 2H), 7.27 (t, J = 8.0 Hz, 2H), 7.12 (t, J = 8.0 Hz, 1H), 7.04 (d, J = 8.0 Hz, 2H), 4.43 (d, J = 12.0 Hz, 1H), 4.15 (d, J = 16.0 Hz, 2H), 3.51 (dd, J = 4.0, 12.0 Hz, 1H), 3.44-3.40 (m, 2H), 3.21 (d, J = 16.0 Hz, 1H). ^{13}C NMR (100 MHz, DMSO): δ 187.2, 165.7, 164.6, 136.7, 135.9, 132.1, 128.5, 127.6, 127.5, 127.4, 125.4, 120.9, 120.1, 119.1, 117.5, 115.8, 115.6, 112.8, 55.7, 48.4, 48.2, 29.0. IR (neat, ν cm^{-1}): 3422, 3236, 2924, 1686, 1583, 1428, 1323, 1264, 1197, 967. HRMS (EI+) m/z calcd. for $C_{27}H_{22}FN_3O_3$ $[M+H]^+$ 456.1718, found 456.1723. M.P- 132 ˚C. The purity (88.21%) was determined by HPLC on a stationary phase [Agilent Eclipse Plus C18 column, pump mode used Gradient with A (acetonitrile) and B (0.1 % formic acid in water) as solvent system for 11 minutes), t_R = 6.063 min].

1-benzyl-3-((2-(4-chlorobenzoyl)-1H-indol-3-yl)methyl)piperazine-2,5-dione (**8p**). Prepared according to general procedure from **6d** (tryptophan methyl ester (500.00 mg, 2.29 mmol) and 4-chlorobenzaldehyde (0.27 ml, 1.83 mmol)) and was purified by column chromatography with ethyl acetate-hexane (3:7) as eluent. It was generated as pale yellow solid with yield of 62%. ^{1}H NMR (400 MHz, $CDCl_3$): δ 9.16 (s, 1H), 7.84 (d, J = 8.0 Hz, 1H), 7.60 (d, J = 8.0 Hz, 2H), 7.48 (d, J = 8.0 Hz, 2H), 7.43 (s, 1H), 7.39 (d, J = 8.0 Hz, 2H), 7.25 (d, J = 4.0 Hz, 2H), 7.05 (t, J = 4.0 Hz, 2H), 6.58 (s, 1H), 4.66 (d, J = 12.0 Hz, 1H), 4.41 (s, 1H), 4.08 (d, J = 16.0 Hz, 1H), 3.78 (dd, J = 4.0, 12.0 Hz, 1H), 3.68 (dd, J = 4.0, 12.0 Hz, 1H), 3.45 (d, J = 16.0 Hz, 1H), 2.97 (d, J = 16.0 Hz, 1H), 2.75 (s, 1H). ^{13}C NMR (100 MHz, $CDCl_3$): δ 188.9, 166.1, 165.6, 138.6, 136.7, 135.1, 132.6, 129.3, 128.9, 128.8, 128.4, 128.1, 127.7, 126.9, 121.7, 121.5, 118.8, 112.6, 56.4, 49.7, 48.5, 29.9, 28.9. IR (neat, ν cm^{-1}): 3175, 2920, 2851, 1678, 1645, 1372, 1172, 909. HRMS (EI+) m/z calcd. for $C_{27}H_{22}ClN_3O_3$ $[M+H]^+$ 472.1422, found 472.1431. M.P- 156 ˚C. The purity (99.24%) was determined by HPLC on a stationary phase [Agilent Eclipse Plus C18 column, pump mode used Gradient with A (acetonitrile) and B (0.1 % formic acid in water) as solvent system for 11 minutes), t_R = 8.108 min].

The Assay of TDO/IDO

The working reaction mixture (200µL) contains 20 µL of 0.5 M potassium phosphate buffer pH- 6.5, 40 µL of 0.2 M sodium ascorbate pH- 7.0, 8 µL of 0.5 mM methylene blue, 4 µL of 5mg/ml catalase and 86 µL of dd water was added in to the deep well plate and incubate for 10 min at 35 ˚C. After 10 min

20 µL of IDO1/TDO2 protein (1µg/ml concentration) was added and incubates for 10 min. After that 20 µL of 2 mM L-tryptophan was added and then treated with different test compounds concentration (10 µM, 5 µM, 1 µM, 500 nM, 250 nM). The plate was then sealed well for 2hr incubation process at 37°C. After incubation, 40 µL of 30% TCA solution was added and again incubated for 15 min at 60 ˚C, the samples were centrifuged for 15 min at 13000 rpm at 4 ˚C. The 100 µL of supernatant was transferred in fresh 96 well plate and treated with 100 µL of freshly prepared 2% Ehlrich reagent which was then analyzed by absorbance plate reader at 492 nm to measure the extent of kynurenine formation. The DMSO content in the sample was 0.005 %. The final concentration of the compound in deep well plate was 5 µM. The IC50 values were calculated in triplicate by using GraphPadPrism Software (GraphPadPrism. Version 5.01 program).[22, 23]

For controls:

1. Water was substituted with L-tryphtophan

2. Water was substituted with L-tryhtophan and rhIDO/ rhTDO protein.

Calculations:

% Inhibition = 100-(A/B*100)

A = IDO activity in presence of inhibitor

B = IDO activity in absence of inhibitor

Kinetic solubility: Solubility of the compounds was determined by incubating the compound at 500 µM concentration in phosphate buffer saline, pH7.4 for 18 hours with constant vortex at 500 rpm. After incubation period, samples were centrifuged and supernatant was injected into HPLC to determine the solubility.

Details of DMPK studies:

Log P determination: The partition coefficient was determined by adding compounds to 2 mL deep well plate containing Octanol/water mixture and vortexed the plate for 2 hours at 1200 rpm on a plate shaker. After incubation, the samples were allowed to equilibrate for 20 minutes and then samples were centrifuged at 4000 rpm for 30 min. Octanol and buffer layers were analysed by HPLC-UV by adjusting the needle height for each Octanol and buffer and the amount of compound distributed into each phase was calculated

Log D determination: The distribution coefficient was determined by adding compounds to 2 mL deep well plate containing Octanol/Buffer mixture and vortexed the plate for 2 hours at 1200 rpm on a plate shaker. After incubation, the samples were allowed to equilibrate for 20 minutes and then samples were centrifuged at 4000 rpm for 30 min. Octanol and buffer layers were analysed by HPLC-UV by

adjusting the needle height for each Octanol and buffer and the amount of compound distributed into each phase was calculated.

PAMPA permeability: PAMPA permeability was determined by addition of the compounds to the donor wells at 100µM concentration in a total volume of 300µL in triplicates. The acceptor wells were filled with 200µL of phosphate buffered saline, pH 7.4, while maintaining 5% DMSO concentration on both sides. The plates were sandwiched and incubated for 5hours at room temperature. After incubation, 150µL of the sample from both donor and receiver wells was collected and centrifuged at 4000rpm for 20 minutes. The supernatant was analysed by HPLC and the apparent permeability was calculated.

Metabolic stability in mouse, rat and human liver microsomes: Metabolic stability was defined as the percentage of parent compound lost over time in the presence of a metabolically active test system. The incubation mixtures consisted of liver microsomes 1.0 mg microsomal protein /mL , compound 1 uM or positive control verapamil, 1 uM . The reactions were initiated by adding 1 mM NADPH. Reactions without NADPH at 0 and 60 min were also incubated to rule out non-NADPH metabolism or chemical instability in the incubation buffer. All reactions were terminated using 200 uL of ice-cold acetonitrile containing internal standard 200 ng/mL of telmisartan at 0, 5, 15, 30 and 60 min. The vials were centrifuged at 4000 rpm for 20 min. The supernatants thus obtained were analyzed on LC-MS/MS to monitor the disappearance of compound.

Stability in rat and human plasma: Plasma stability is defined as the percentage of parent compound lost over time. The frozen plasma was thawed at room temperature and centrifuged at 1400 rpm 4 °C, for 15 minutes. Approximately 90% of the clear supernatant fraction was transferred to a separate tube and was used for assay. The incubation mixtures consisted of human and rat palsma and compound (1 µM) or positive control (propanthelin, 1 µM) and incubated for 120 min at 37°C in shaker with 500 rpm. All reactions were terminated using 200 µL of ice-cold acetonitrile containing internal standard (150 ng/mL of telmisartan) at 0, 15, 30, 60 and 120 min and centrifuged at 4000 rpm (Eppendorf, Germany) for 20min. The supernatants thus obtained were analyzed on LC-MS/MS to monitor the disappearance of compound.

Plasma protein binding in rat and human plasma: To evaluate the ability of compound to bind the plasma proteins, the most common approach of plasma protein binding using rapid equilibrium dialysis device (RED inserts) used. Compounds were tested at a final concentration of 10 µM in mouse, rat and human plasma. An aliquot of 500 µL plasma containing compound 3k was added in first half (plasma side) of the well of RED device. An aliquot of 300 µL of 100 mM sodium phosphate buffer pH 7.4 was added in the second half (buffer side) of the Insert. The plate containing plasma and

buffer was equilibrated at 37 ± 1 °C for 6h, with constant shaking at 500 rpm on an orbital shaker. Samples were collected from respective halves after the completion of the incubation time. The proteins were precipitated using organic solvents. The samples were subjected to centrifugation and the supernatants were analysed on LC-MS/MS.

Coordinates for QM region:

c 48.299742 47.358975 35.067182
n 48.825027 47.956218 36.194324
h 49.753180 48.377575 36.255580
c 47.970585 47.712437 37.220553
h 48.138196 48.047924 38.240103
n 46.929353 47.009158 36.811746
c 47.113548 46.787704 35.469200
h 46.386376 46.216491 34.897811
n 44.548178 45.282109 36.415226
c 43.474033 45.752203 35.715899
c 44.807058 44.030913 35.913691
c 42.991750 44.744267 34.770950
c 43.833316 43.662751 34.870923
c 45.894926 43.257719 36.328228
h 46.091363 42.337243 35.775777
c 46.762636 43.513585 37.398069
n 46.651339 44.575578 38.253687
c 47.596966 44.397895 39.223707
c 48.381172 43.195628 38.944865
c 47.854383 42.635595 37.803387
c 47.779191 45.255206 40.317112
h 48.523371 44.940474 41.053715
c 47.188366 46.509536 40.506058
c 47.576034 47.490443 41.522628
c 46.093033 48.357917 40.051634
n 46.279496 47.065634 39.643343
c 46.926645 48.663600 41.207650
c 45.256676 49.284853 39.418156
h 45.229311 50.274347 39.878996
c 44.452778 49.083886 38.293864
c 43.615074 50.099084 37.649159
n 44.322643 47.881217 37.662143
c 43.408138 48.076300 36.668327
c 42.978297 47.055687 35.815061
h 42.176455 47.298824 35.117409
fe 45.424464 46.187272 38.021226
c 42.934489 49.473359 36.635932
o 44.193240 45.270158 39.198627
o 42.976399 45.701191 39.434592
c 37.507842 43.610134 42.201282
c 37.708794 43.216004 40.857919
c 38.613693 43.880162 40.031876
c 39.322168 44.965742 40.573063
c 39.137275 45.377062 41.933120
c 38.208268 44.685901 42.739872
n 40.236021 45.815186 40.009692
c 40.672414 46.722705 40.949408
c 40.019587 46.492307 42.160728
c 40.136029 47.283199 43.436642
c 39.487656 48.675106 43.380509
n 39.786915 49.443213 44.578932
c 40.211468 50.740458 44.542393
o 40.201565 51.415289 43.520465
c 41.525627 47.879452 40.517998
o 41.334831 48.407320 39.441254
c 40.720693 51.295853 45.860414
h 36.764858 43.088813 42.812175
h 37.113845 42.394047 40.449228
h 38.742863 43.590888 38.985819
h 38.025598 44.999490 43.772485
h 40.747696 45.699141 39.134367
h 41.192371 47.415425 43.718751
h 39.672934 46.708007 44.256101
h 38.401553 48.574633 43.224631
h 39.857908 49.259677 42.528269
h 39.907226 48.930548 45.444767
h 41.796256 51.505409 45.753660
h 40.587565 50.623915 46.717820
h 40.211880 52.249315 46.065770
h 42.058830 44.832680 34.214255
h 43.825708 42.703430 34.353515
h 49.194712 42.810222 39.559423
h 48.146456 41.713196 37.301450
h 48.339939 47.368619 42.290530
h 47.053993 49.657381 41.636890
h 43.629261 51.158972 37.903132
h 42.199299 49.851900 35.925813
h 48.782490 47.395833 34.090625
h 42.229998 48.282773 41.245501

Root Means Square Deviations

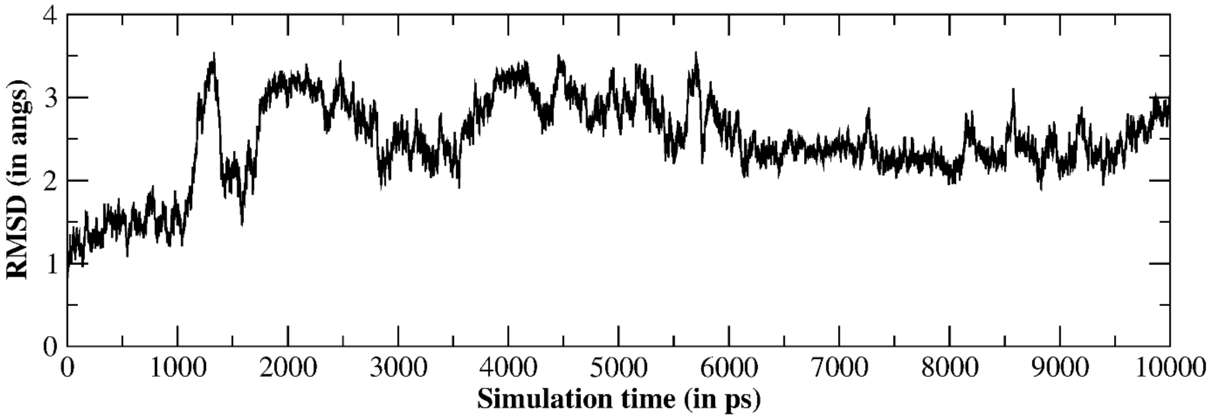

Figure S4. RMS deviation during MD simulations of h-IDO with inhibitor 8d. Note that RMS deviation becomes constant after 60ns which shows a converged MD trajectory. RMSD was calculated for heavy atoms i.e., C,CA, C*, N and O.

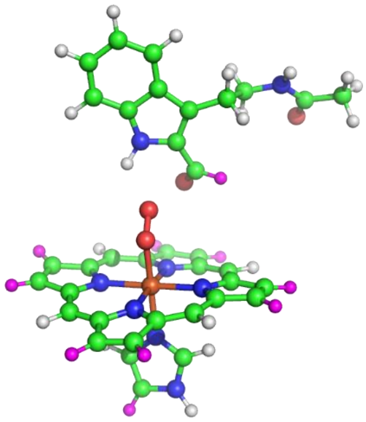

Figure S5. QM geometry with link atoms (shown by magenta)

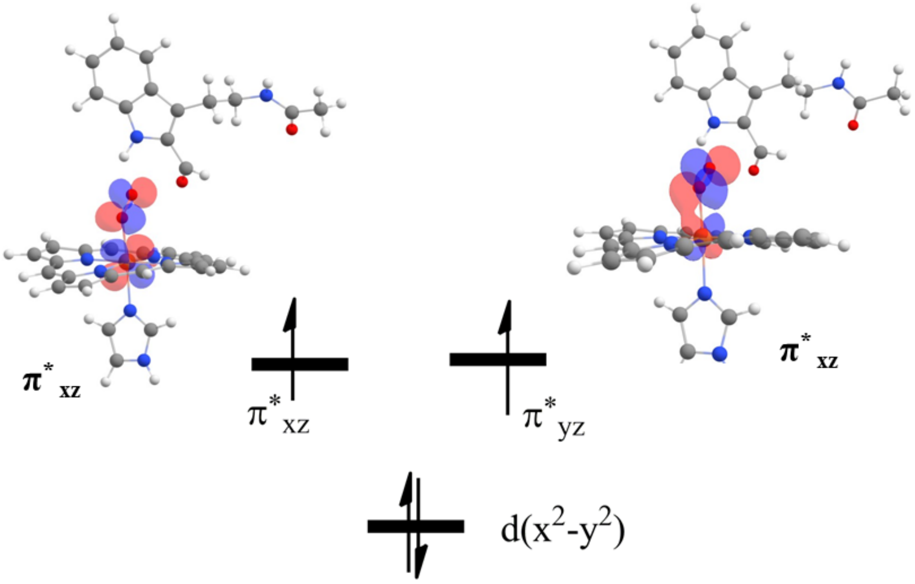

Figure S6. Orbital diagram of the reactant complex in the triplet spin state (ground state).

8a

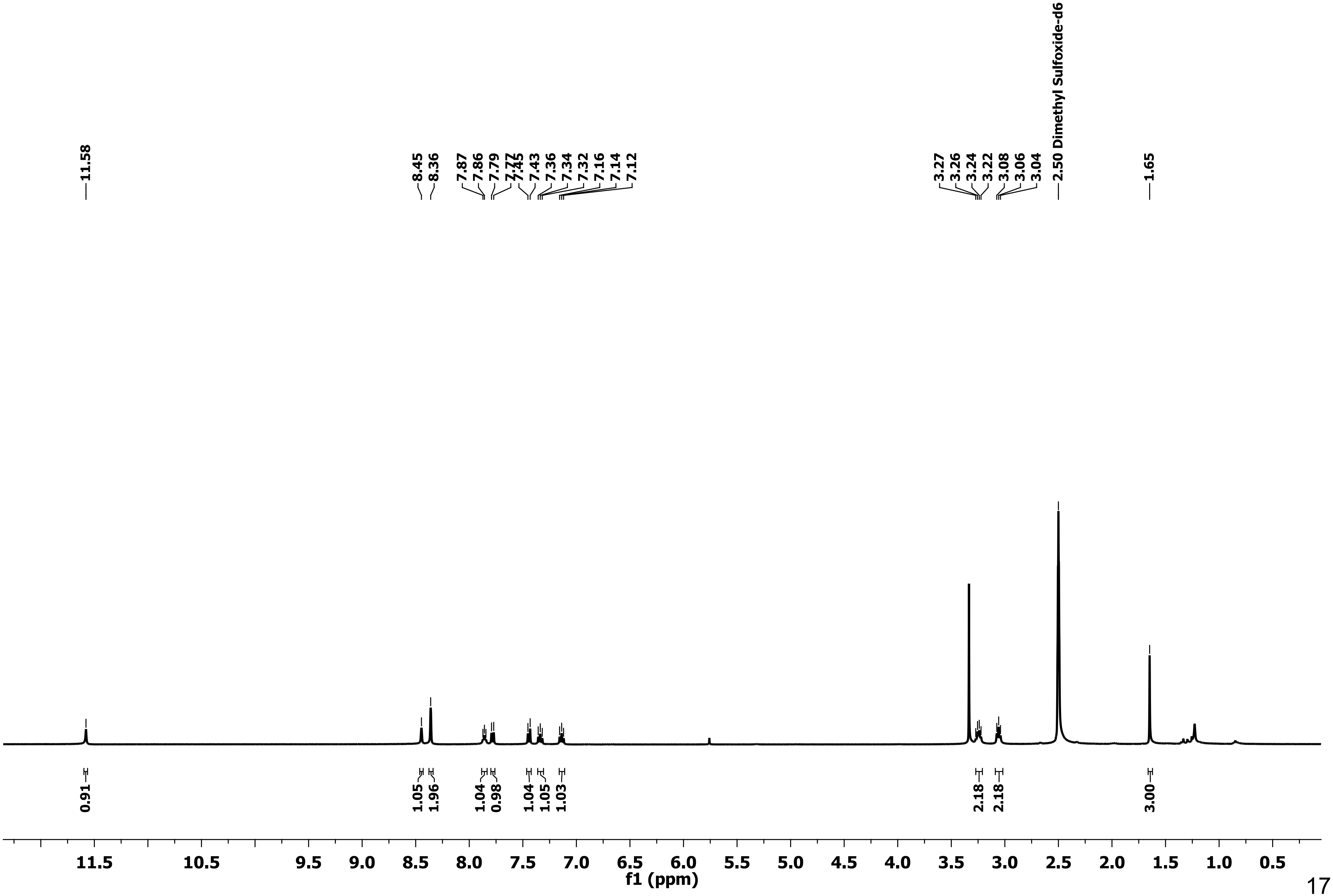
11.58
8.45
8.36
7.87
7.86
7.79
7.77
7.45
7.43
7.36
7.34
7.32
7.16
7.14
7.12
3.27
3.26
3.24
3.22
3.08
3.06
3.04
2.50 Dimethyl Sulfoxide-d6
1.65
0.91
1.05
1.96
1.04
0.98
1.04
1.05
1.03
2.18
2.18
3.00
11.5
10.5
9.5
9.0
8.5
8.0
7.5
7.0
6.5
6.0
5.5
5.0
4.5
4.0
3.5
3.0
2.5
2.0
1.5
1.0
0.5
f1 (ppm)

8a

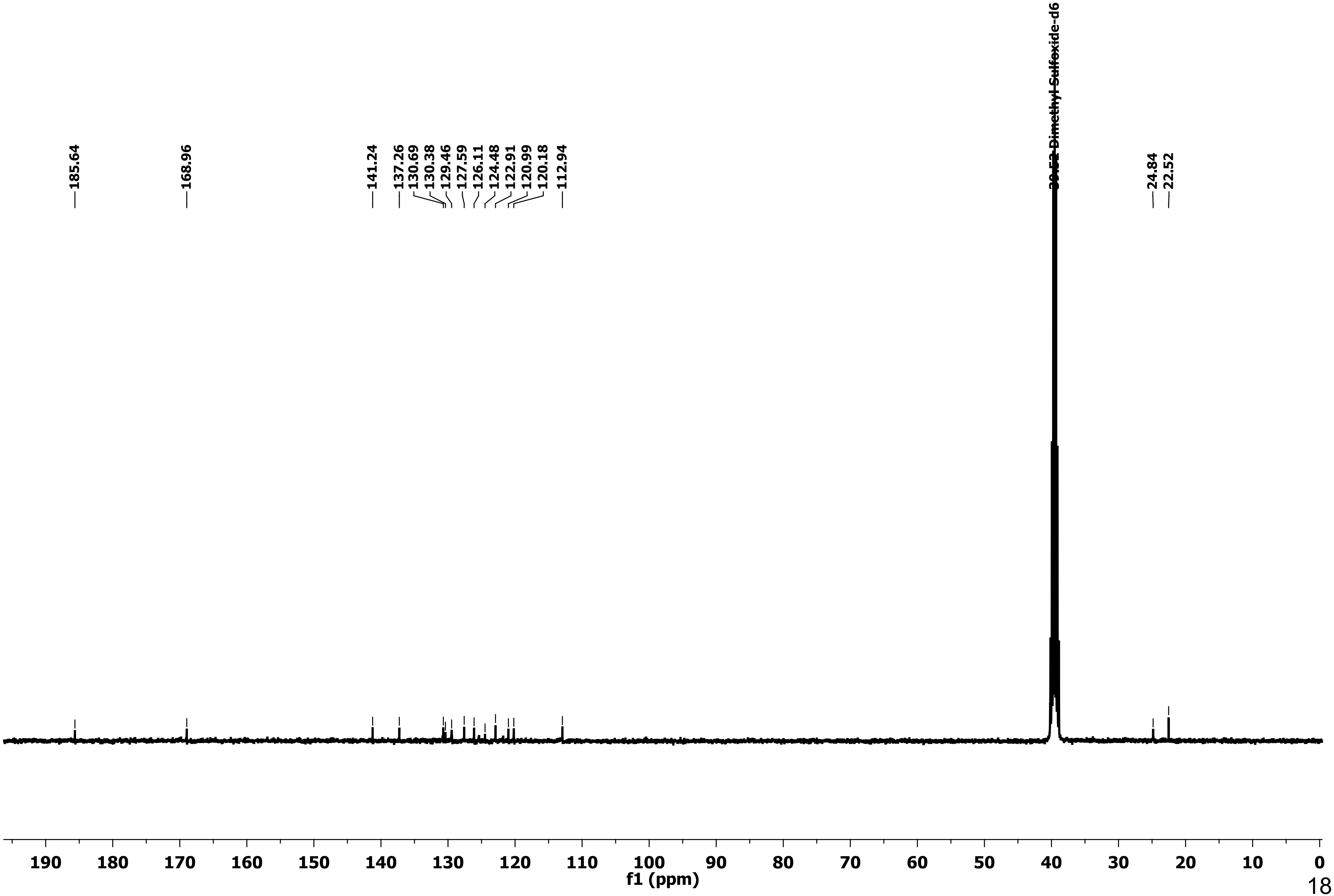

185.64
168.96
141.24
137.26
130.69
130.38
129.46
127.59
126.11
124.48
122.91
120.99
120.18
112.94
39.52 Dimethyl Sulfoxide-d6
24.84
22.52
190 180 170 160 150 140 130 120 110 100 90 80 70 60 50 40 30 20 10 0
f1 (ppm)

8b

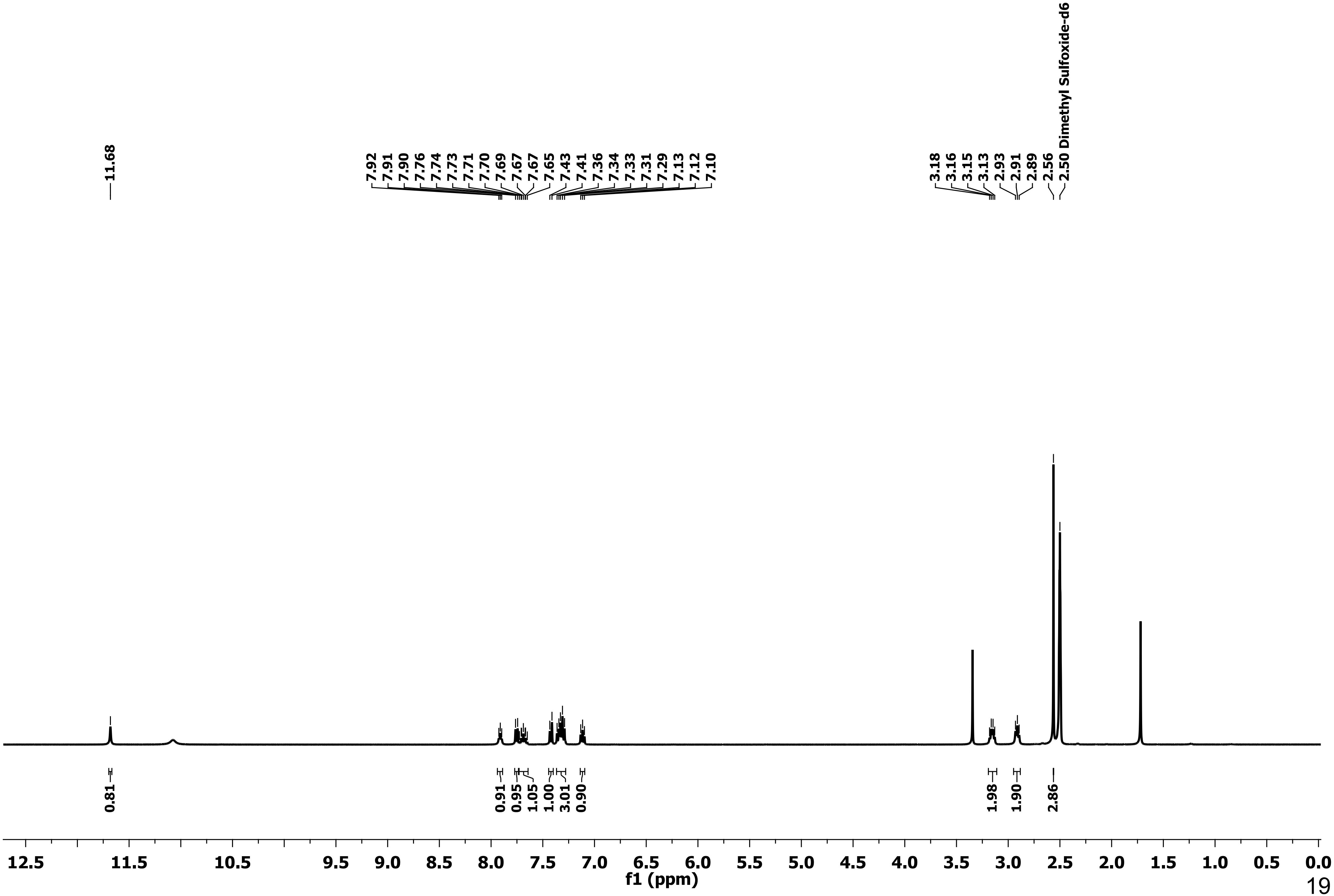

11.68
7.92
7.91
7.90
7.76
7.74
7.73
7.71
7.70
7.69
7.67
7.67
7.65
7.43
7.41
7.36
7.34
7.33
7.31
7.29
7.13
7.12
7.10
3.18
3.16
3.15
3.13
2.93
2.91
2.89
2.56
2.50 Dimethyl Sulfoxide-d6
0.81
0.91
0.95
1.05
1.00
3.01
0.90
1.98
1.90
2.86
12.5
11.5
10.5
9.5
9.0
8.5
8.0
7.5
7.0
6.5
6.0
5.5
5.0
4.5
4.0
3.5
3.0
2.5
2.0
1.5
1.0
0.5
0.0
f1 (ppm)

8b

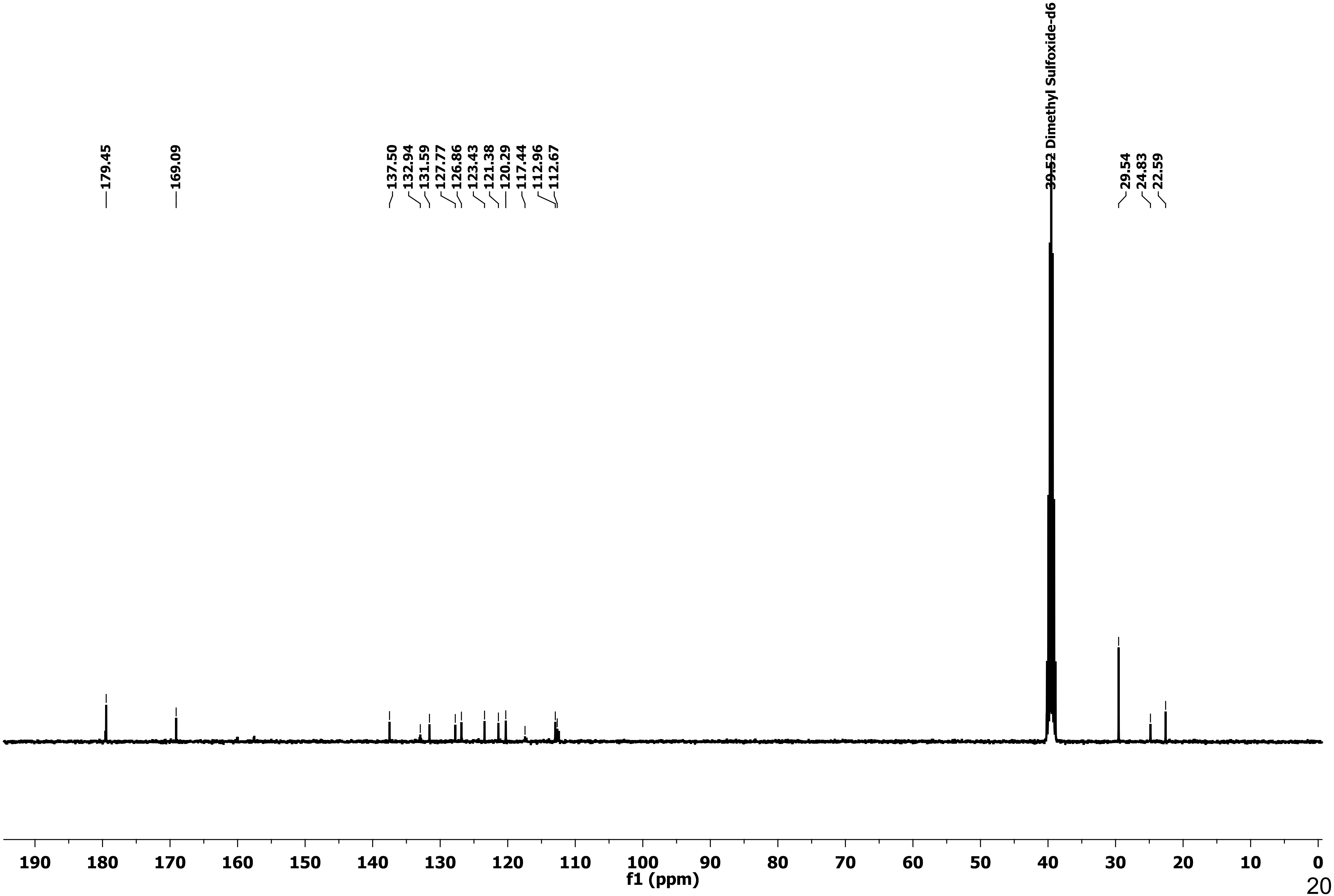
179.45
169.09
137.50
132.94
131.59
127.77
126.86
123.43
121.38
120.29
117.44
112.96
112.67
39.52 Dimethyl Sulfoxide-d6
29.54
24.83
22.59
190
180
170
160
150
140
130
120
110
100
90
80
70
60
50
40
30
20
10
0
f1 (ppm)

8c

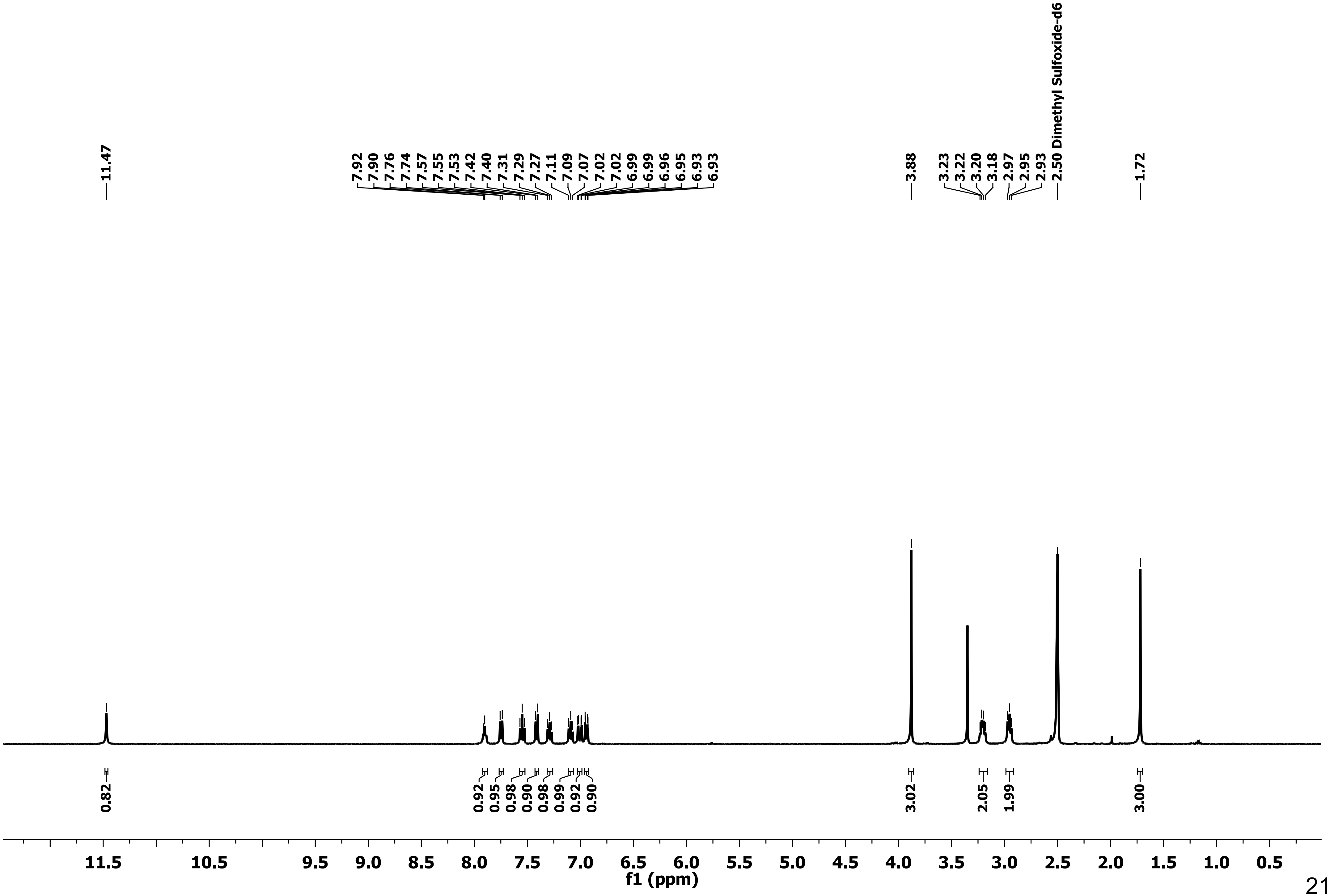

11.47
7.92
7.90
7.76
7.74
7.57
7.55
7.53
7.42
7.40
7.31
7.29
7.27
7.11
7.09
7.07
7.02
7.02
6.99
6.99
6.96
6.95
6.93
6.93
3.88
3.23
3.22
3.20
3.18
2.97
2.95
2.93
2.50 Dimethyl Sulfoxide-d6
1.72
0.82
0.92
0.95
0.98
0.90
0.98
0.99
0.92
0.90
3.02
2.05
1.99
3.00
11.5
10.5
9.5
9.0
8.5
8.0
7.5
7.0
6.5
6.0
5.5
5.0
4.5
4.0
3.5
3.0
2.5
2.0
1.5
1.0
0.5
f1 (ppm)

8c

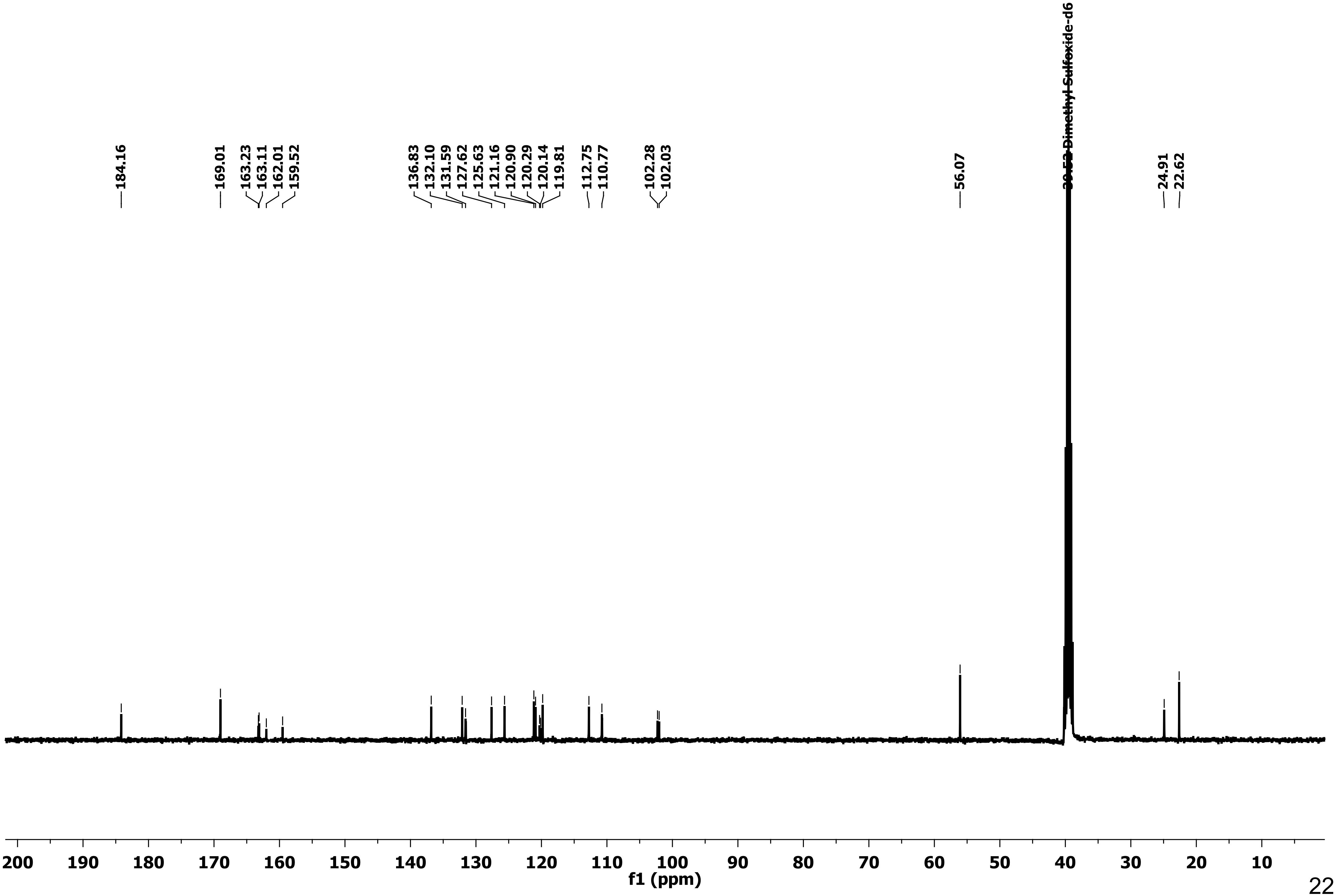

184.16
169.01
163.23
163.11
162.01
159.52
136.83
132.10
131.59
127.62
125.63
121.16
120.90
120.29
120.14
119.81
112.75
110.77
102.28
102.03
56.07
39.52 Dimethyl Sulfoxide-d6
24.91
22.62
200 190 180 170 160 150 140 130 120 110 100 90 80 70 60 50 40 30 20 10
f1 (ppm)

8d

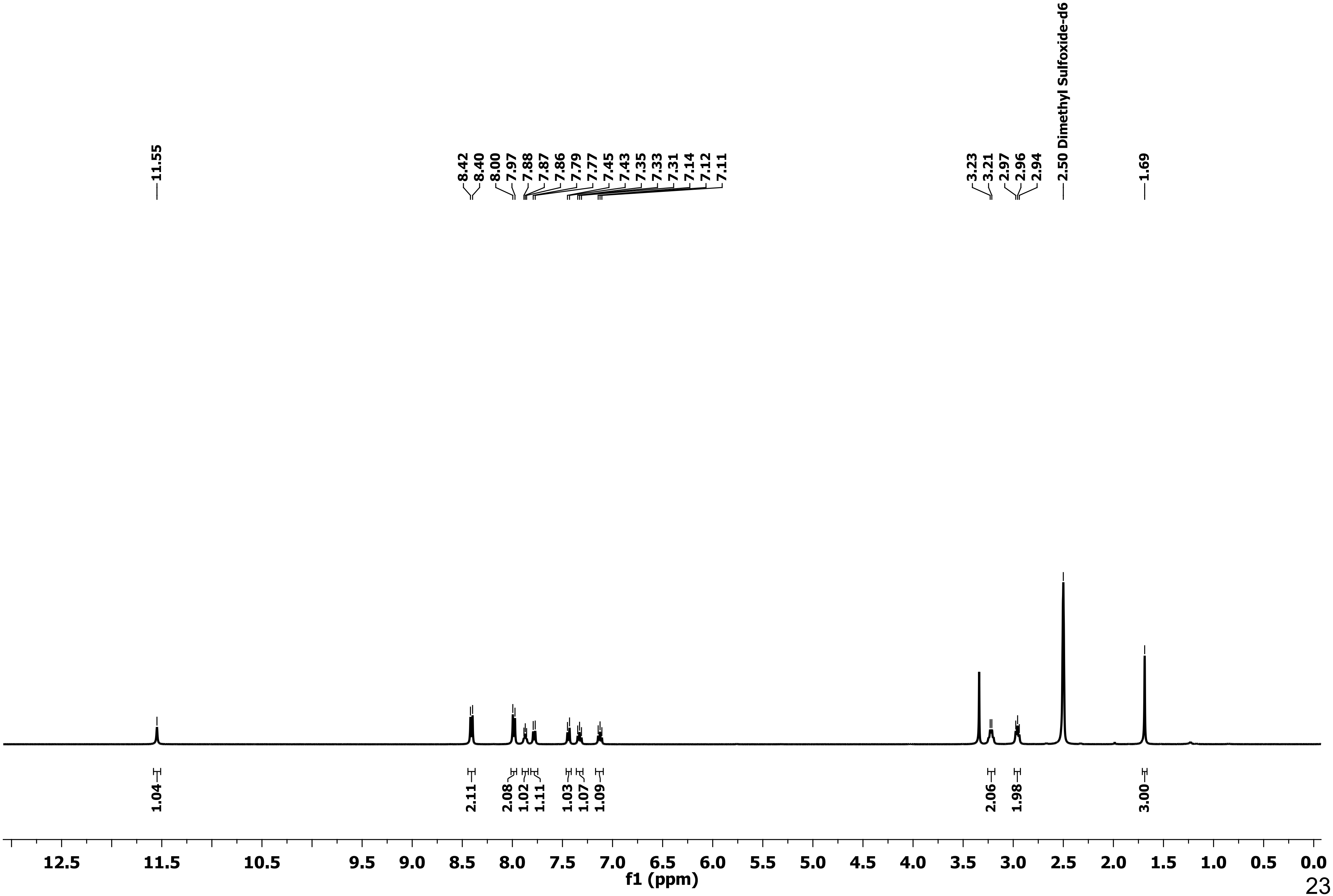
11.55
8.42
8.40
8.00
7.97
7.88
7.87
7.86
7.79
7.77
7.45
7.43
7.35
7.33
7.31
7.14
7.12
7.11
3.23
3.21
2.97
2.96
2.94
2.50 Dimethyl Sulfoxide-d6
1.69
1.04
2.11
2.08
1.02
1.11
1.03
1.07
1.09
2.06
1.98
3.00
12.5
11.5
10.5
9.5
9.0
8.5
8.0
7.5
7.0
6.5
6.0
5.5
5.0
4.5
4.0
3.5
3.0
2.5
2.0
1.5
1.0
0.5
0.0
f1 (ppm)

8d

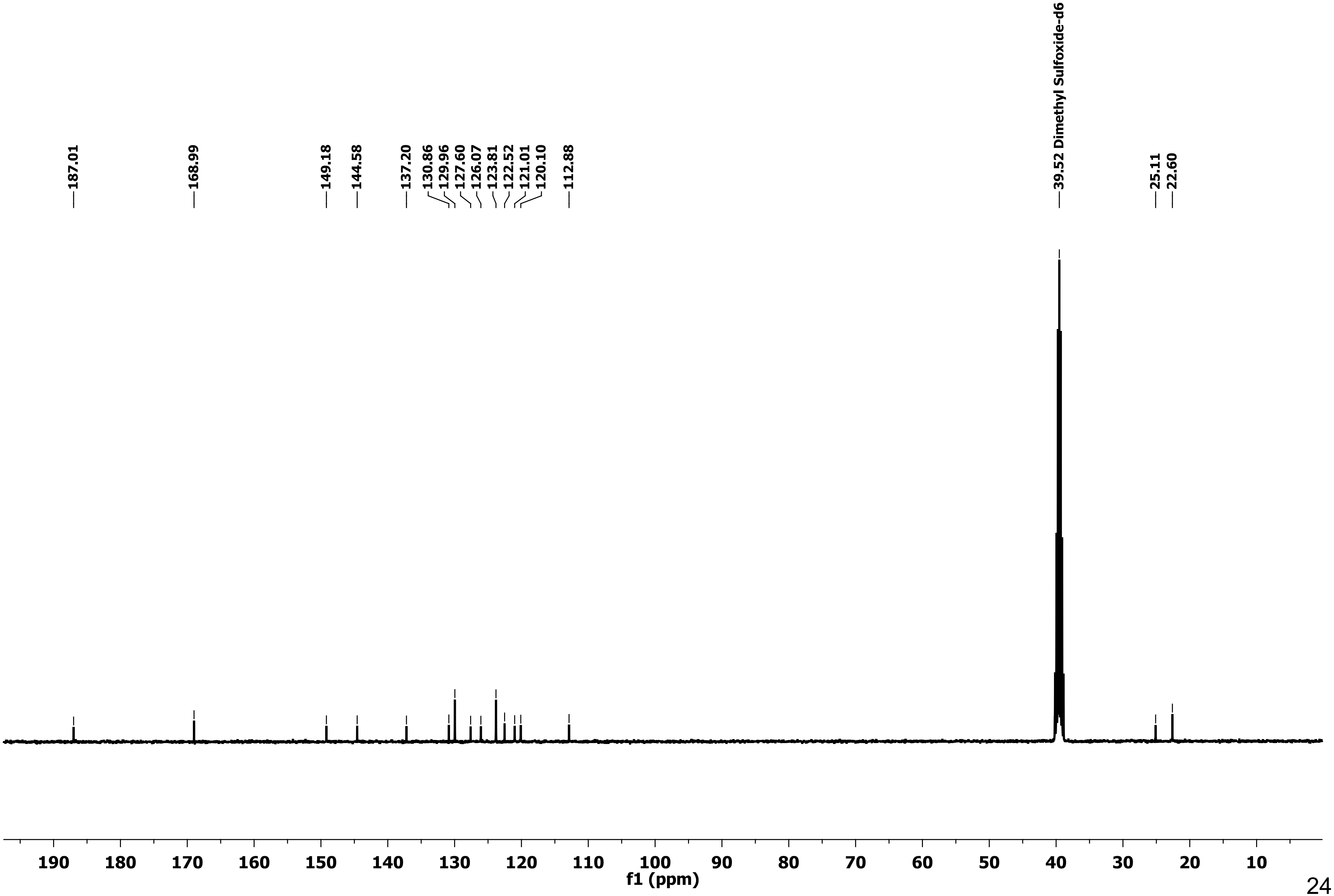
187.01
168.99
149.18
144.58
137.20
130.86
129.96
127.60
126.07
123.81
122.52
121.01
120.10
112.88
39.52 Dimethyl Sulfoxide-d6
25.11
22.60
190
180
170
160
150
140
130
120
110
100
90
80
70
60
50
40
30
20
10
f1 (ppm)

8e

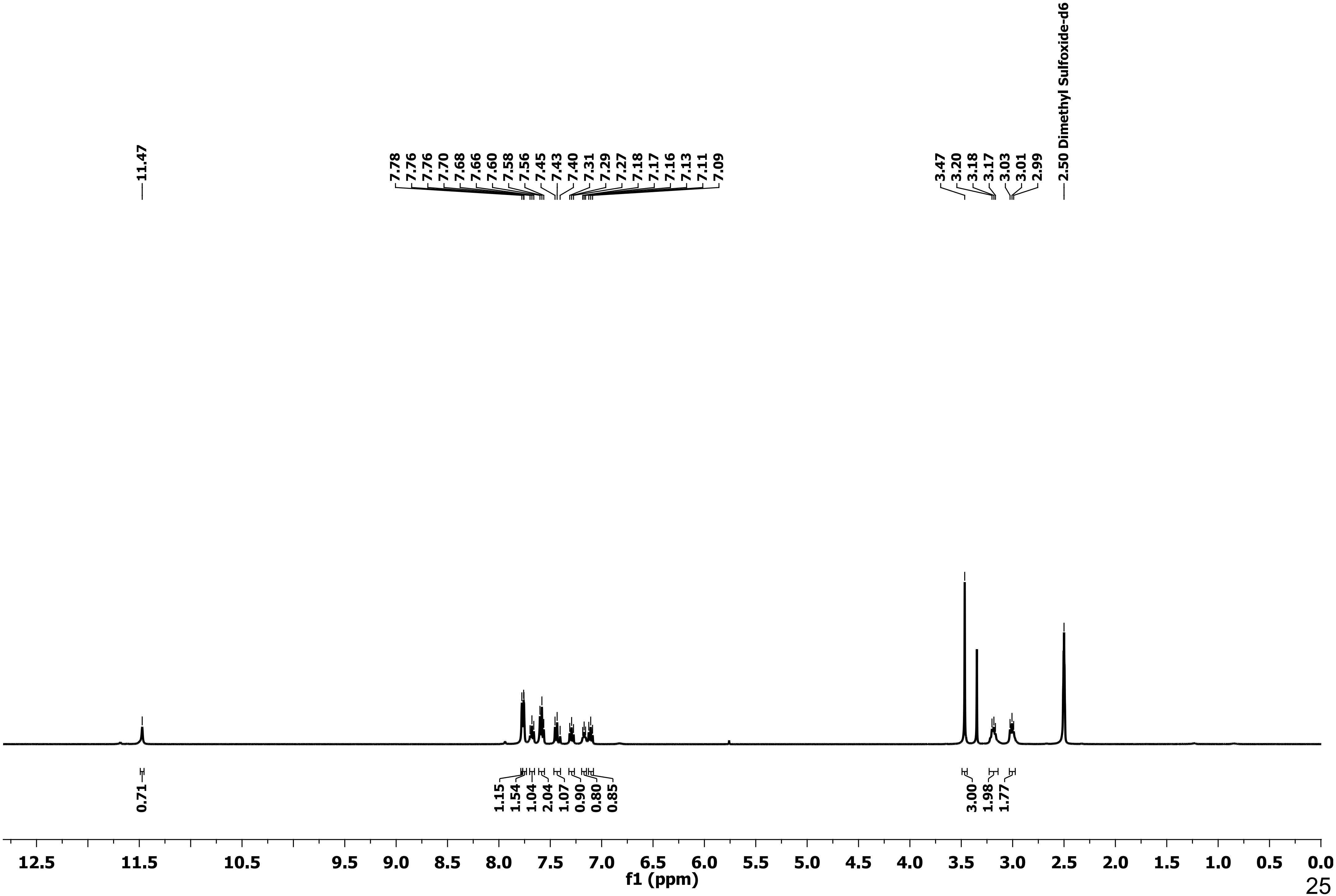

11.47
7.78
7.76
7.76
7.70
7.68
7.66
7.60
7.58
7.56
7.45
7.43
7.40
7.31
7.29
7.27
7.18
7.17
7.16
7.13
7.11
7.09
3.47
3.20
3.18
3.17
3.03
3.01
2.99
2.50 Dimethyl Sulfoxide-d6
0.71
1.15
1.54
1.04
2.04
1.07
0.90
0.80
0.85
3.00
1.98
1.77
12.5
11.5
10.5
9.5
9.0
8.5
8.0
7.5
7.0
6.5
6.0
5.5
5.0
4.5
4.0
3.5
3.0
2.5
2.0
1.5
1.0
0.5
0.0
f1 (ppm)

8e

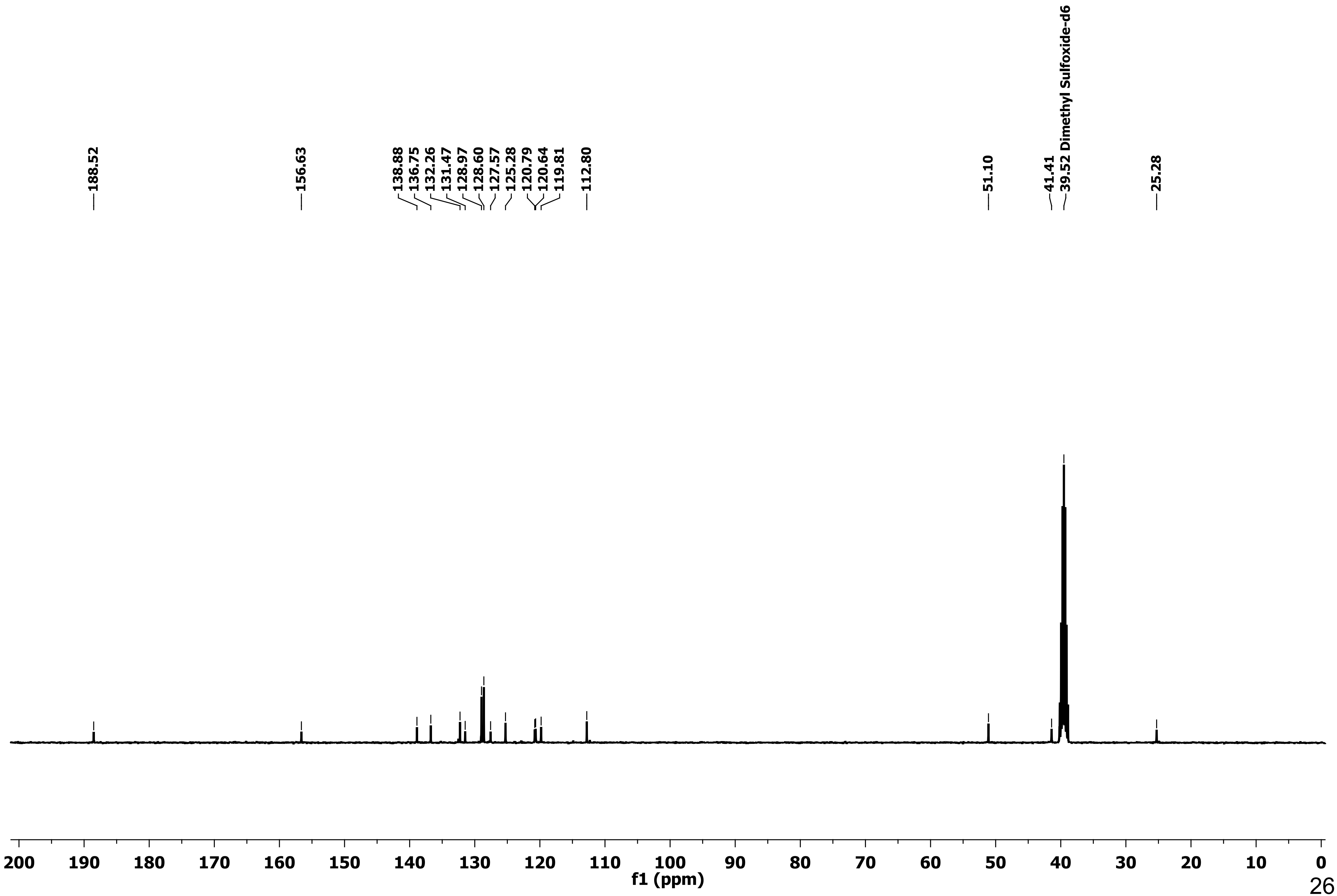
188.52
156.63
138.88
136.75
132.26
131.47
128.97
128.60
127.57
125.28
120.79
120.64
119.81
112.80
51.10
41.41
39.52 Dimethyl Sulfoxide-d6
25.28
200
190
180
170
160
150
140
130
120
110
100
90
80
70
60
50
40
30
20
10
0
f1 (ppm)

8f

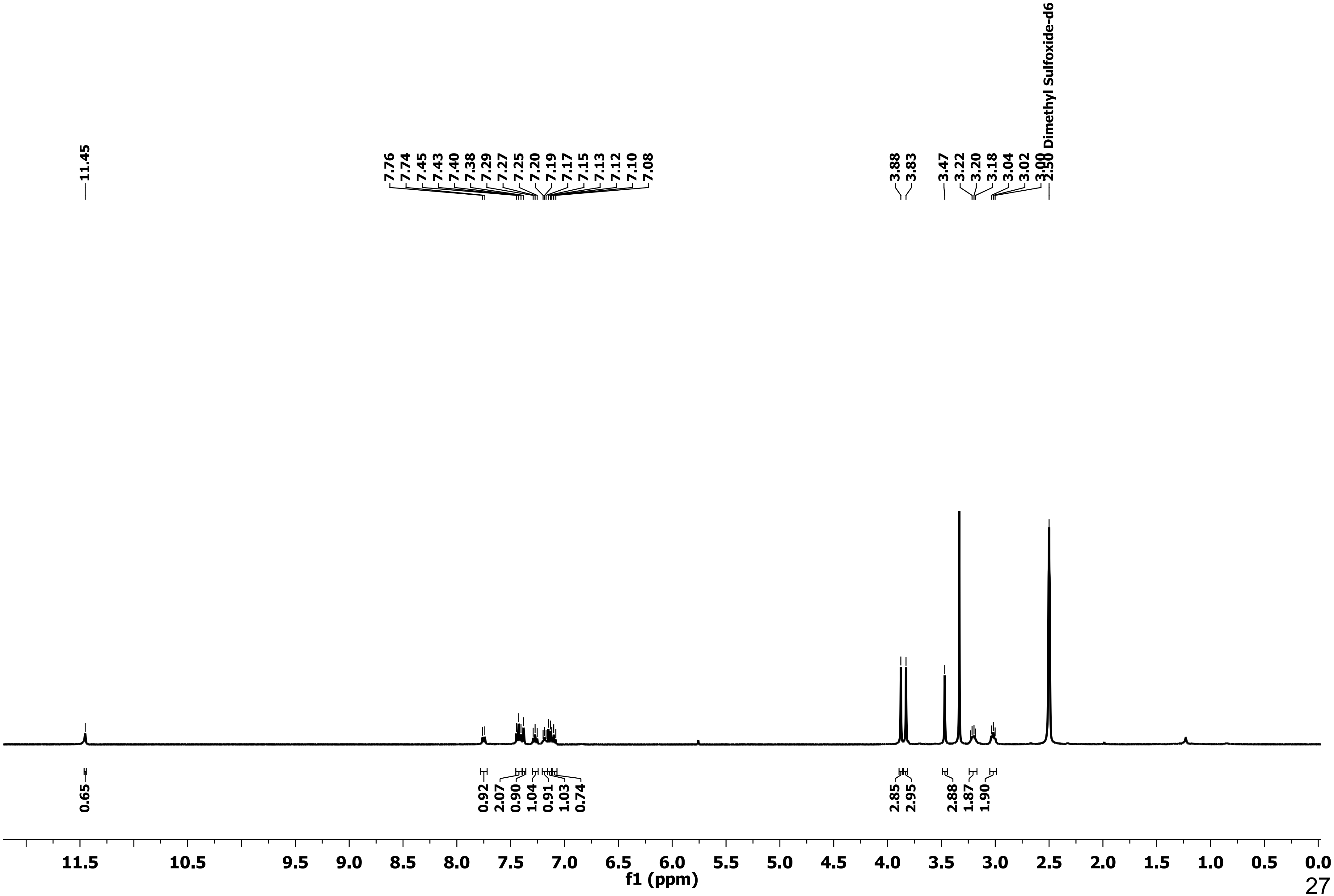
11.45
7.76 7.74 7.45 7.43 7.40 7.38 7.29 7.27 7.25 7.20 7.19 7.17 7.15 7.13 7.12 7.10 7.08
3.88 3.83 3.47 3.22 3.20 3.18 3.04 3.02 3.00 2.50 Dimethyl Sulfoxide-d6
0.65
0.92 2.07 0.90 1.04 0.91 1.03 0.74
2.85 2.95 2.88 1.87 1.90
11.5 10.5 9.5 9.0 8.5 8.0 7.5 7.0 6.5 6.0 5.5 5.0 4.5 4.0 3.5 3.0 2.5 2.0 1.5 1.0 0.5 0.0
f1 (ppm)

8f

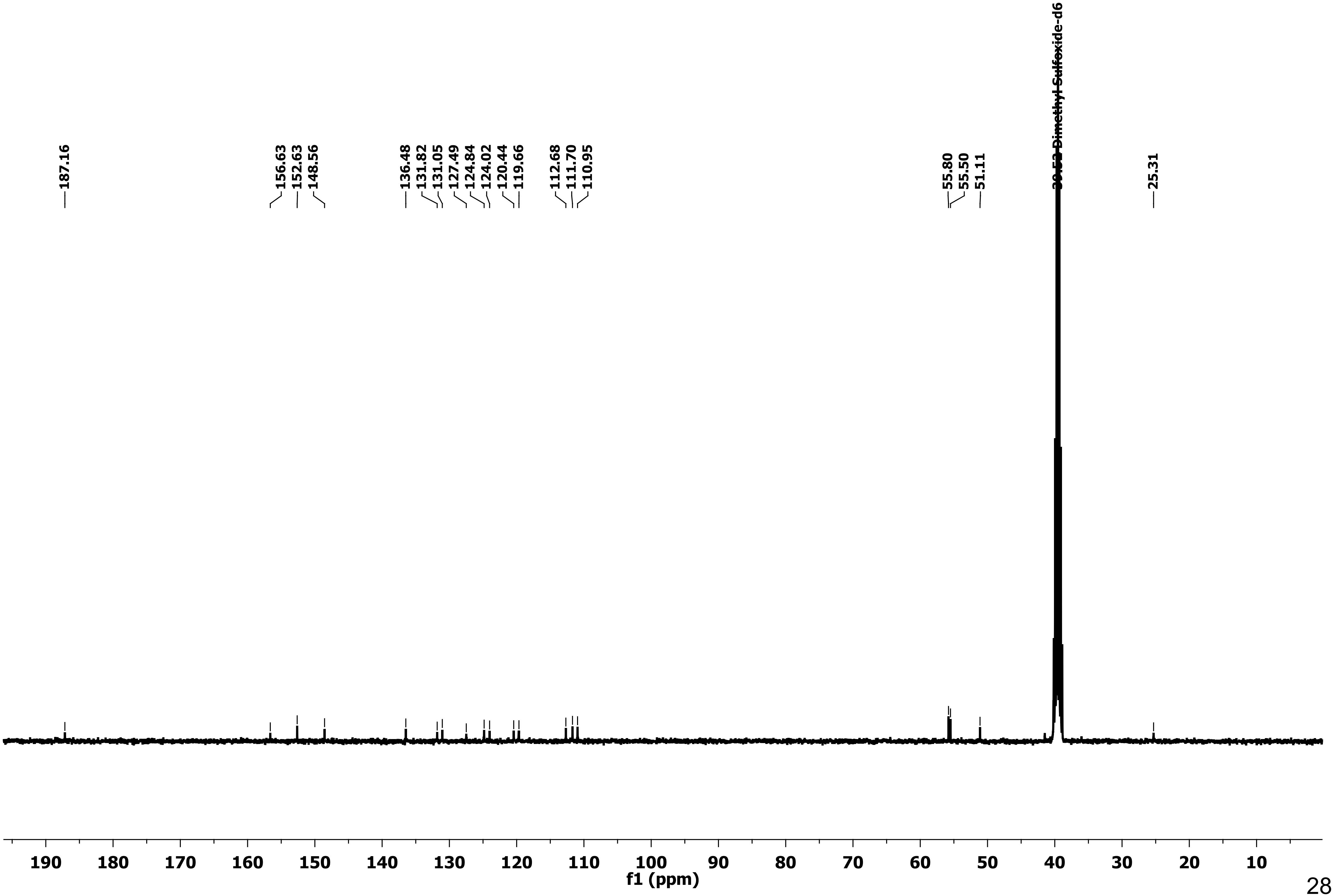
187.16
156.63
152.63
148.56
136.48
131.82
131.05
127.49
124.84
124.02
120.44
119.66
112.68
111.70
110.95
55.80
55.50
51.11
39.52 Dimethyl Sulfoxide-d6
25.31
190 180 170 160 150 140 130 120 110 100 90 80 70 60 50 40 30 20 10
f1 (ppm)

8g

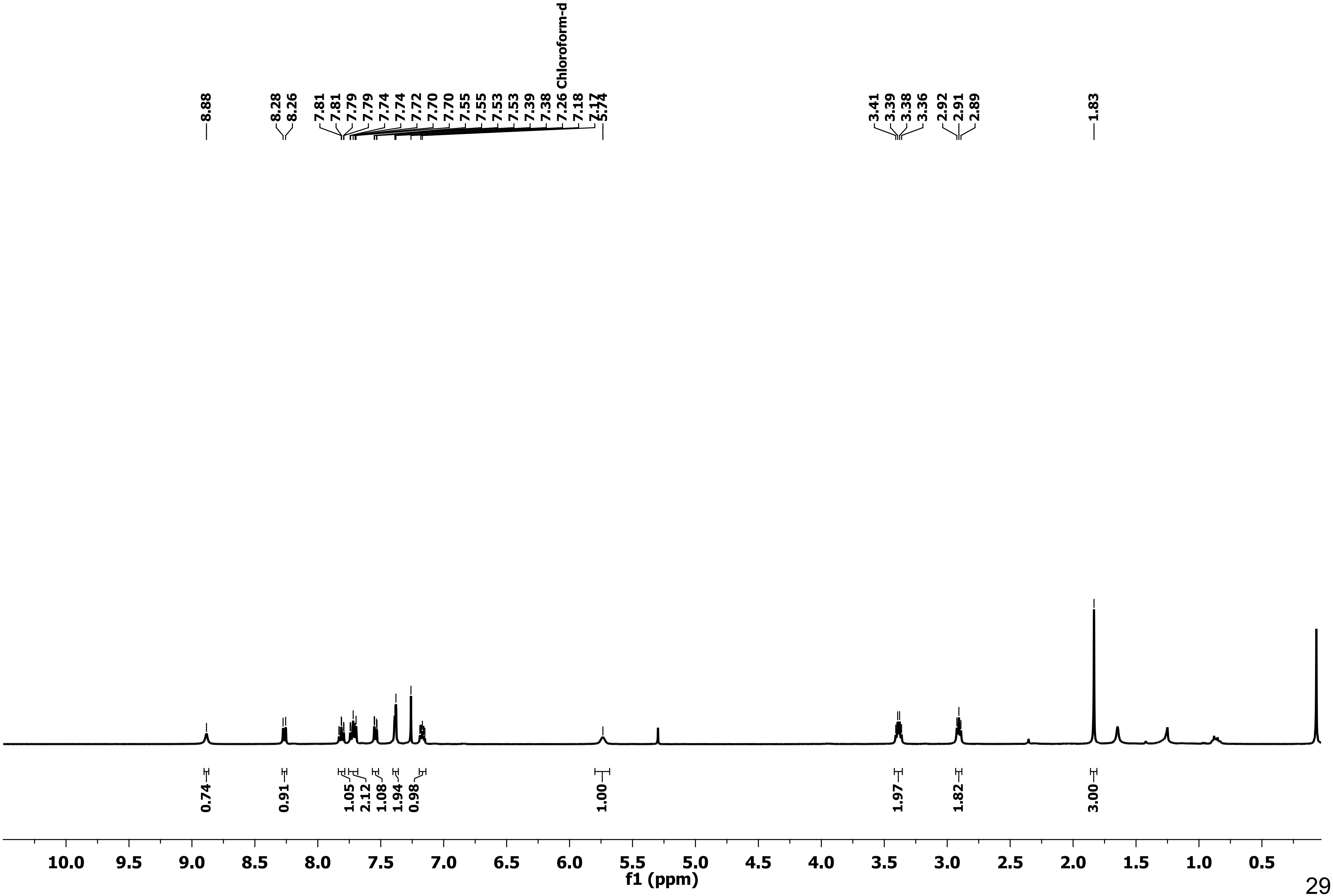
8.88
8.28
8.26
7.81
7.81
7.79
7.79
7.74
7.74
7.72
7.70
7.70
7.55
7.55
7.53
7.53
7.39
7.38
7.26 Chloroform-d
7.18
7.17
5.74
3.41
3.39
3.38
3.36
2.92
2.91
2.89
1.83
0.74
0.91
1.05
2.12
1.08
1.94
0.98
1.00
1.97
1.82
3.00
10.0 9.5 9.0 8.5 8.0 7.5 7.0 6.5 6.0 5.5 5.0 4.5 4.0 3.5 3.0 2.5 2.0 1.5 1.0 0.5
f1 (ppm)

8g

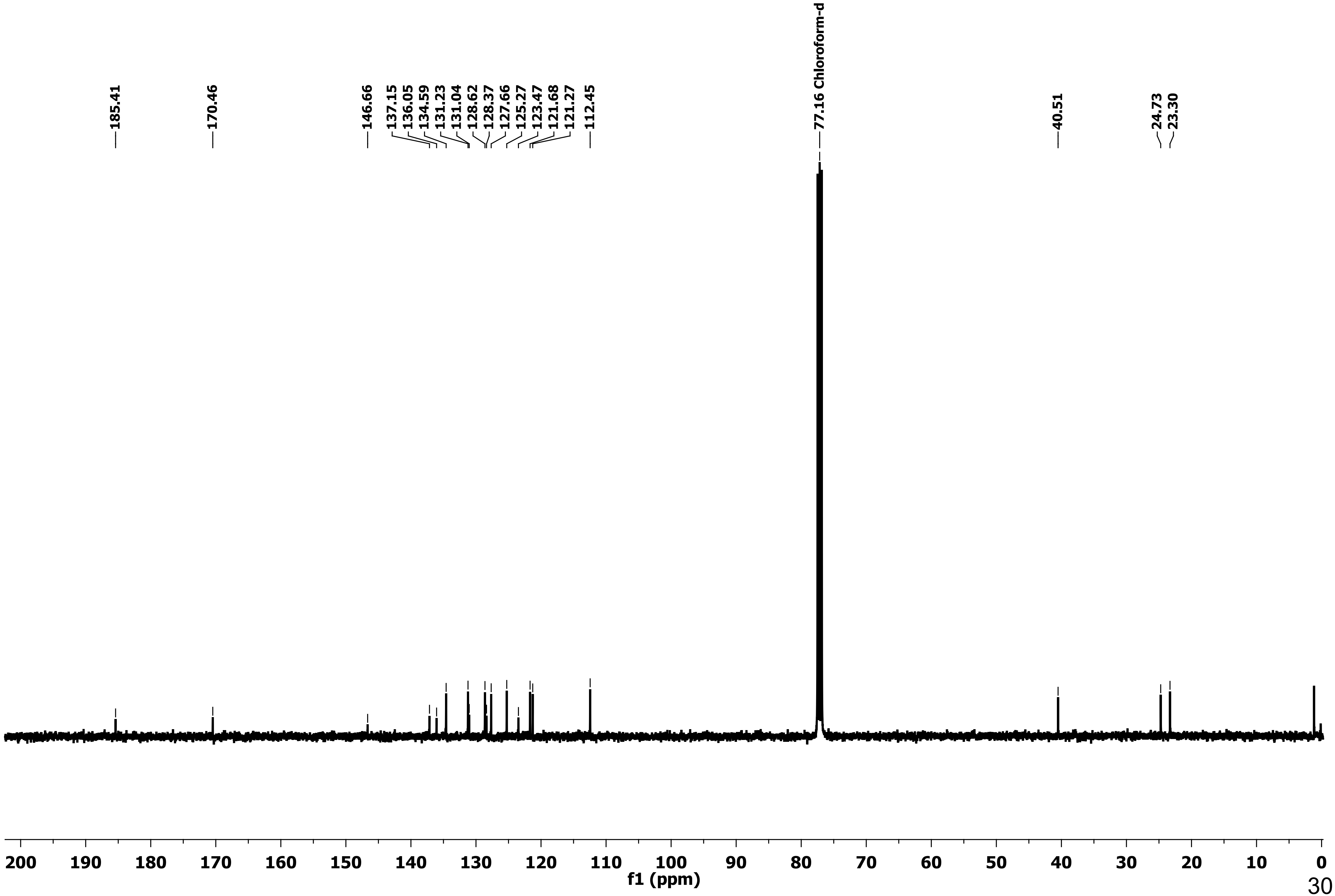
185.41
170.46
146.66
137.15
136.05
134.59
131.23
131.04
128.62
128.37
127.66
125.27
123.47
121.68
121.27
112.45
77.16 Chloroform-d
40.51
24.73
23.30
200 190 180 170 160 150 140 130 120 110 100 90 80 70 60 50 40 30 20 10 0
f1 (ppm)

8h

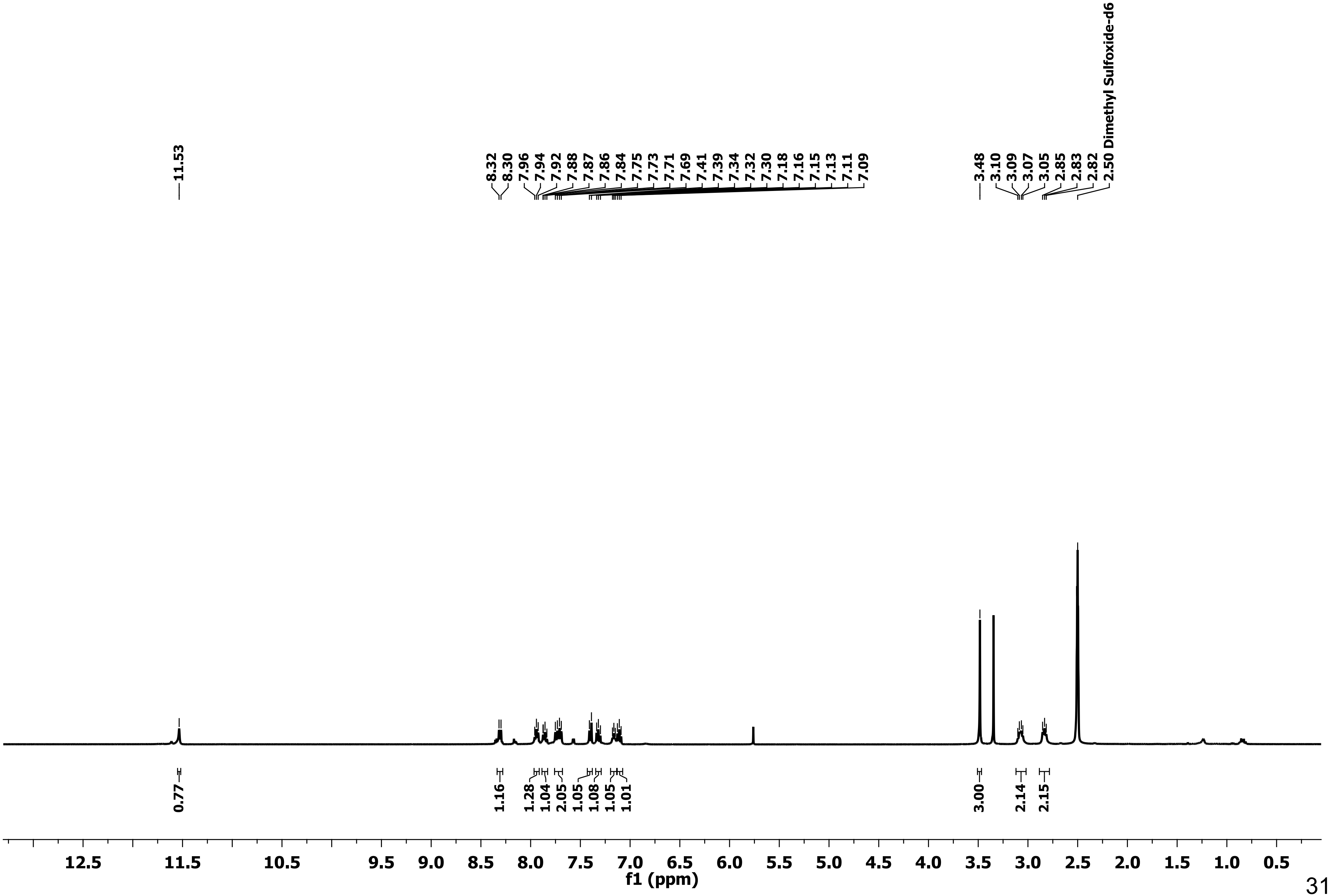
11.53
8.32
8.30
7.96
7.94
7.92
7.88
7.87
7.86
7.84
7.75
7.73
7.71
7.69
7.41
7.39
7.34
7.32
7.30
7.18
7.16
7.15
7.13
7.11
7.09
3.48
3.10
3.09
3.07
3.05
2.85
2.83
2.82
2.50 Dimethyl Sulfoxide-d6
0.77
1.16
1.28
1.04
2.05
1.05
1.08
1.05
1.01
3.00
2.14
2.15
f1 (ppm)

8h

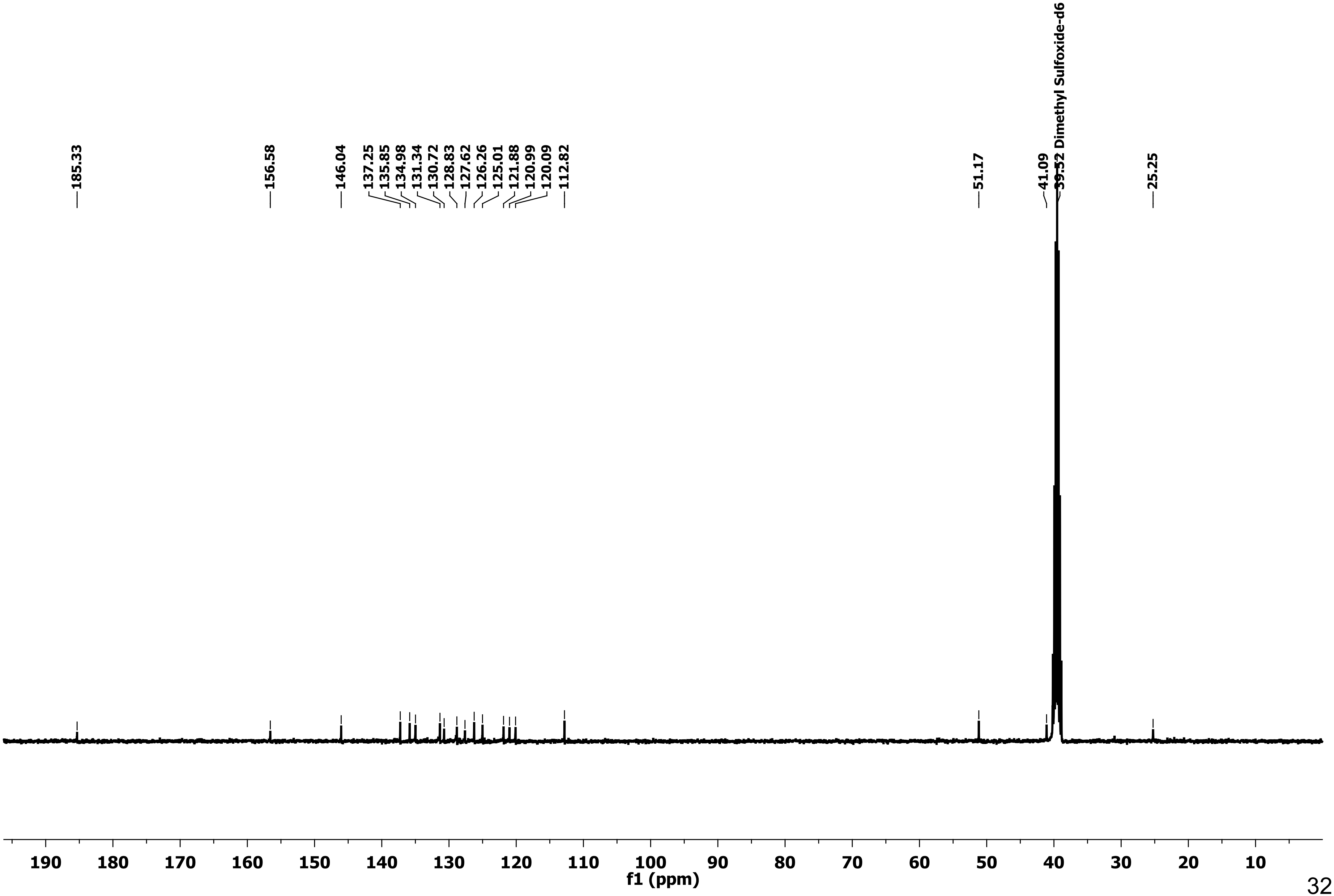
185.33
156.58
146.04
137.25
135.85
134.98
131.34
130.72
128.83
127.62
126.26
125.01
121.88
120.99
120.09
112.82
51.17
41.09
39.52 Dimethyl Sulfoxide-d6
25.25
190
180
170
160
150
140
130
120
110
100
90
80
70
60
50
40
30
20
10
f1 (ppm)

8i

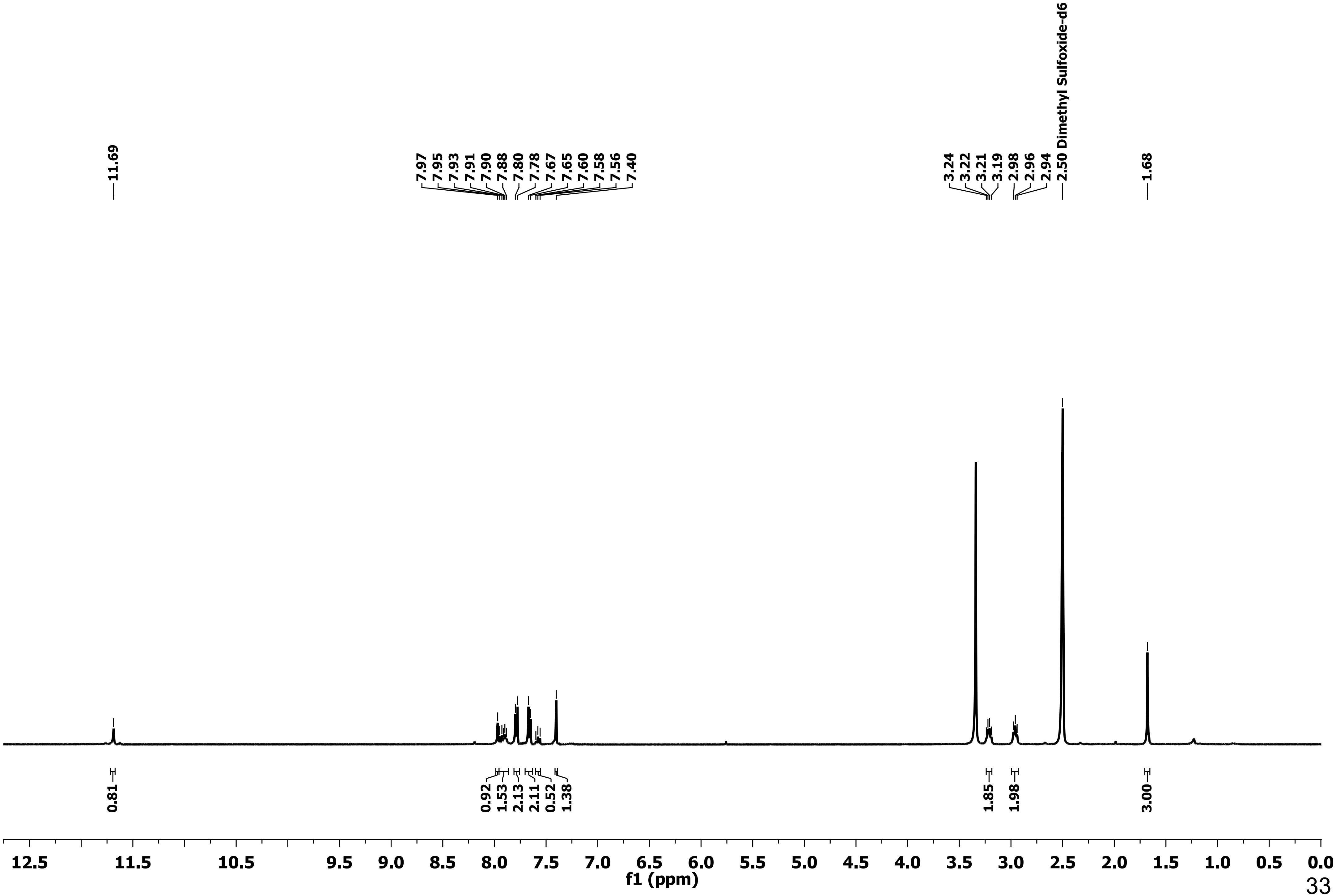
11.69
7.97
7.95
7.93
7.91
7.90
7.88
7.80
7.78
7.67
7.65
7.60
7.58
7.56
7.40
3.24
3.22
3.21
3.19
2.98
2.96
2.94
2.50 Dimethyl Sulfoxide-d6
1.68
0.81
0.92
1.53
2.13
2.11
0.52
1.38
1.85
1.98
3.00
12.5 11.5 10.5 9.5 9.0 8.5 8.0 7.5 7.0 6.5 6.0 5.5 5.0 4.5 4.0 3.5 3.0 2.5 2.0 1.5 1.0 0.5 0.0
f1 (ppm)

8i

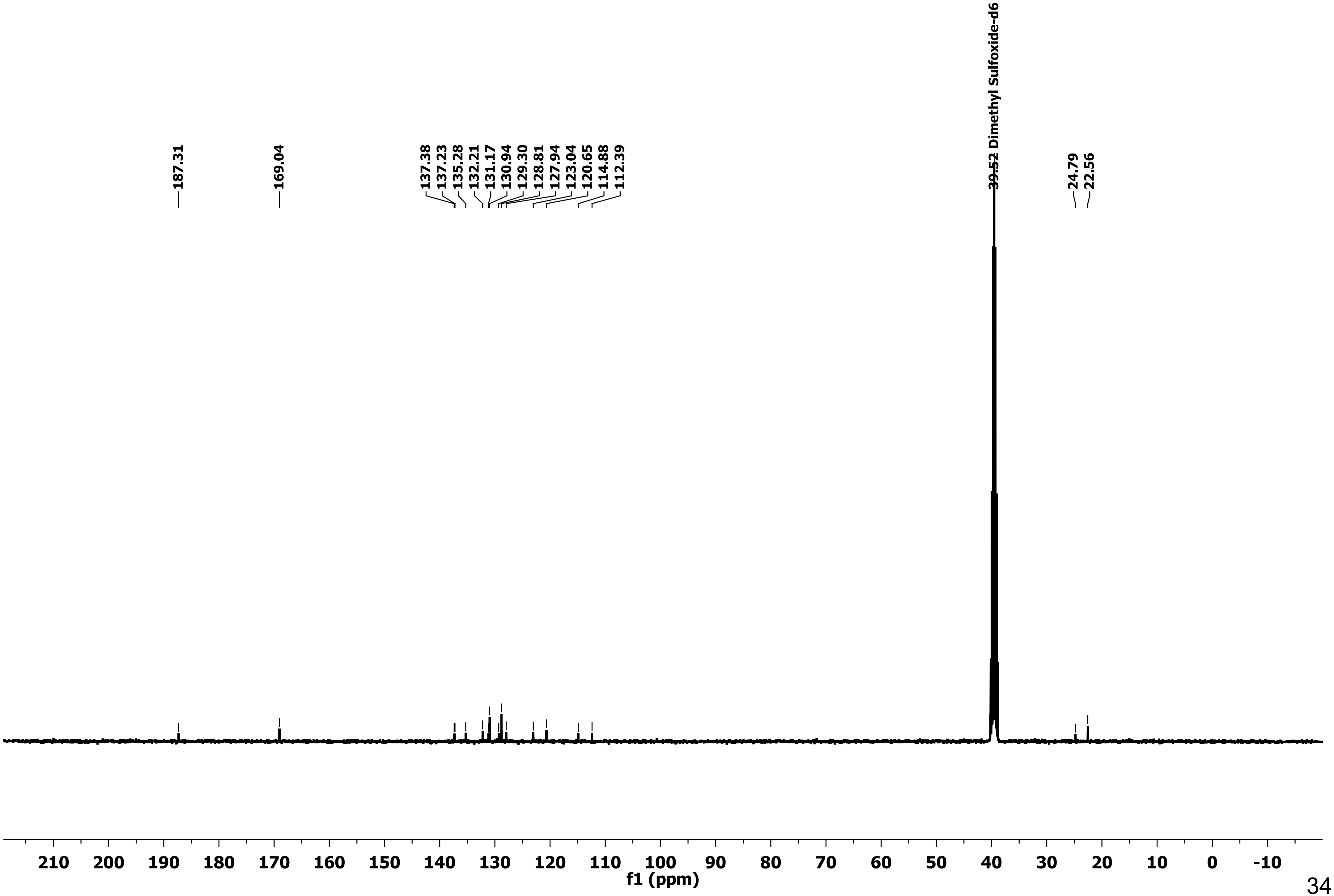
187.31
169.04
137.38
137.23
135.28
132.21
131.17
130.94
129.30
128.81
127.94
123.04
120.65
114.88
112.39
39.52 Dimethyl Sulfoxide-d6
24.79
22.56
210 200 190 180 170 160 150 140 130 120 110 100 90 80 70 60 50 40 30 20 10 0 -10
f1 (ppm)

8j

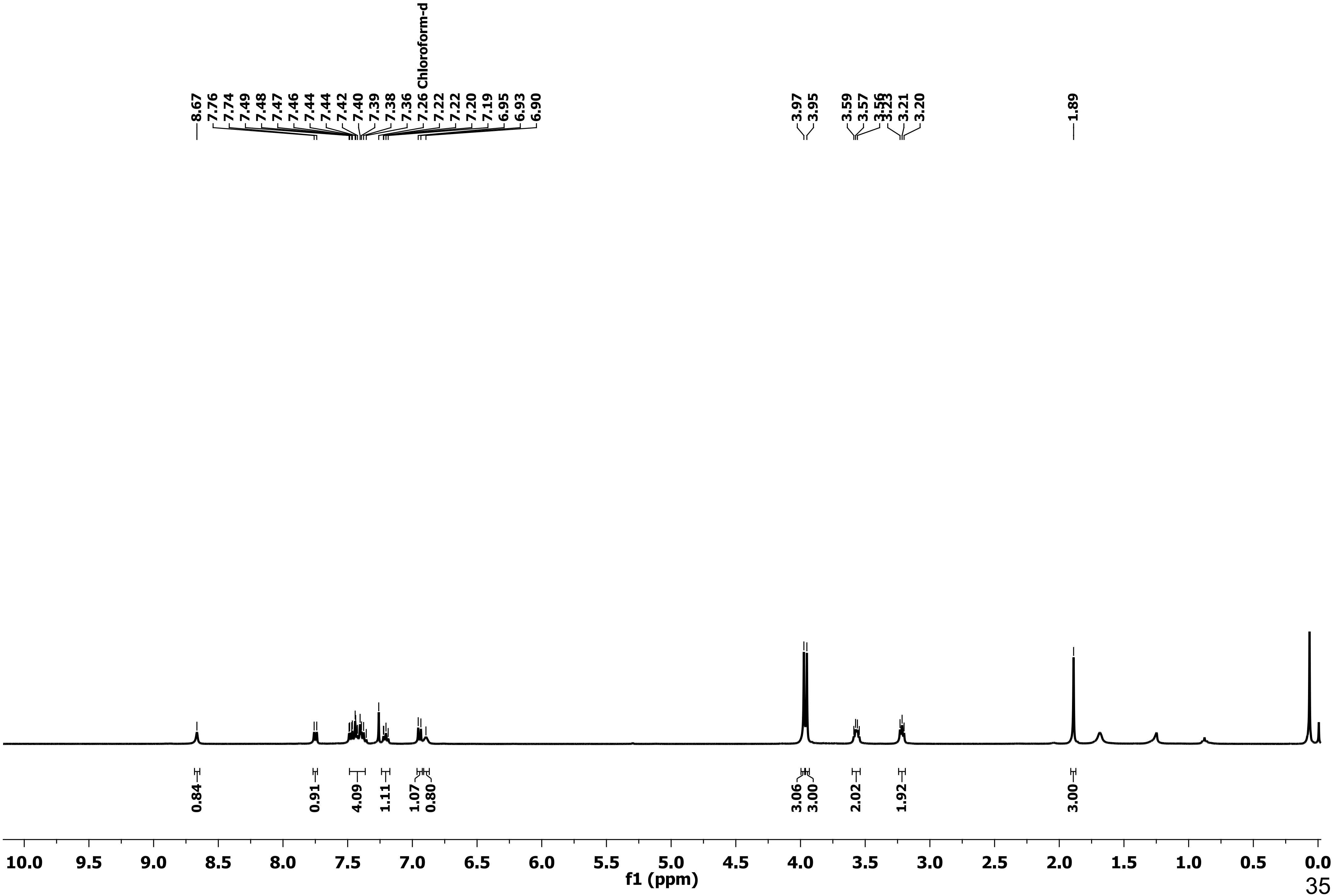
Chloroform-d
8.67
7.76
7.74
7.49
7.48
7.47
7.46
7.44
7.44
7.42
7.40
7.39
7.38
7.36
7.26
7.22
7.22
7.20
7.19
6.95
6.93
6.90
3.97
3.95
3.59
3.57
3.56
3.23
3.21
3.20
1.89
0.84
0.91
4.09
1.11
1.07
0.80
3.06
3.00
2.02
1.92
3.00
10.0
9.5
9.0
8.5
8.0
7.5
7.0
6.5
6.0
5.5
5.0
4.5
4.0
3.5
3.0
2.5
2.0
1.5
1.0
0.5
0.0
f1 (ppm)

8j

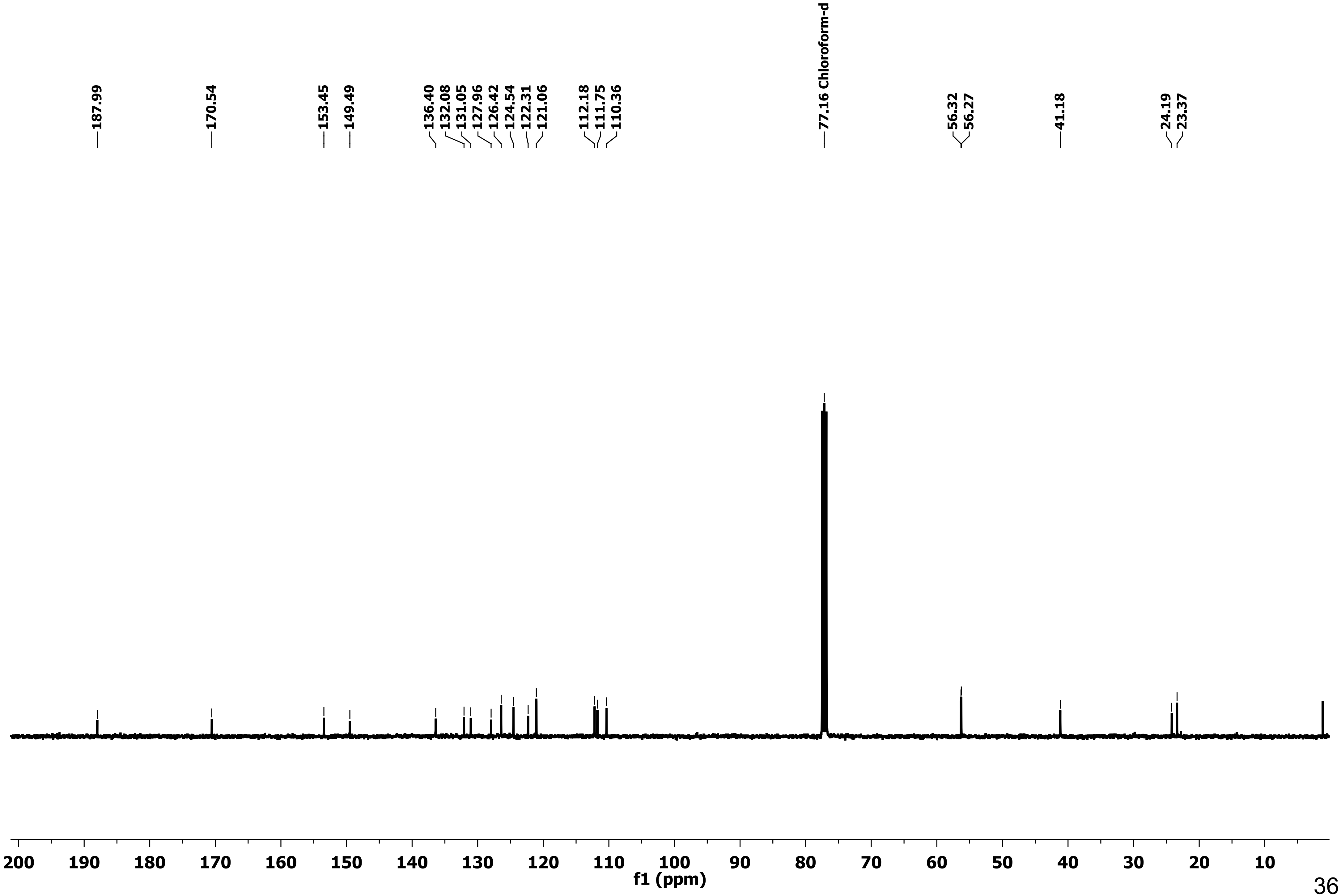
187.99
170.54
153.45
149.49
136.40
132.08
131.05
127.96
126.42
124.54
122.31
121.06
112.18
111.75
110.36
77.16 Chloroform-d
56.32
56.27
41.18
24.19
23.37
200 190 180 170 160 150 140 130 120 110 100 90 80 70 60 50 40 30 20 10
f1 (ppm)

8k

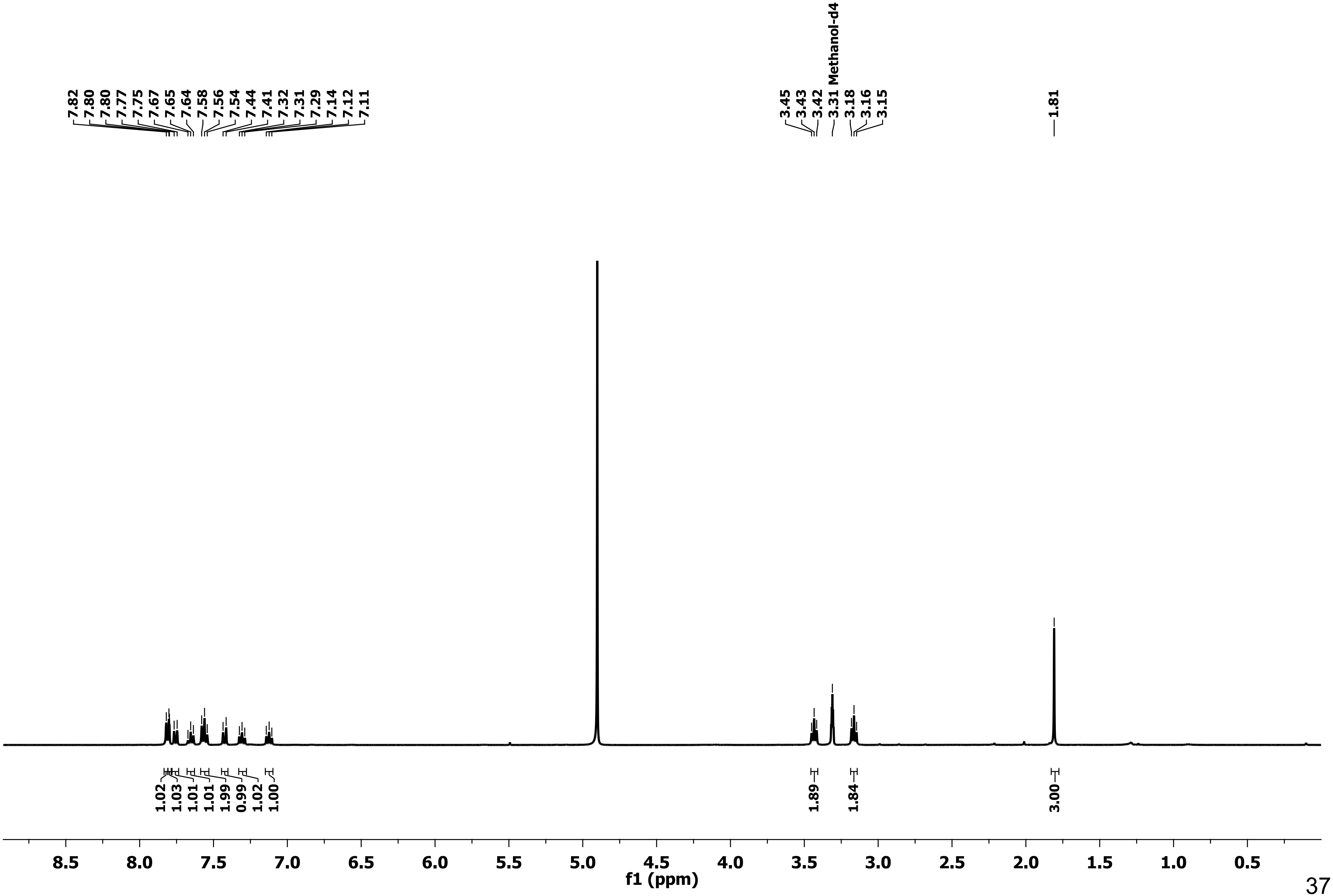
7.82
7.80
7.80
7.77
7.75
7.67
7.65
7.64
7.58
7.56
7.54
7.44
7.41
7.32
7.31
7.29
7.14
7.12
7.11
3.45
3.43
3.42
3.31 Methanol-d4
3.18
3.16
3.15
1.81
1.02
1.03
1.01
1.01
1.99
0.99
1.02
1.00
1.89
1.84
3.00
8.5
8.0
7.5
7.0
6.5
6.0
5.5
5.0
4.5
4.0
3.5
3.0
2.5
2.0
1.5
1.0
0.5
f1 (ppm)

8k

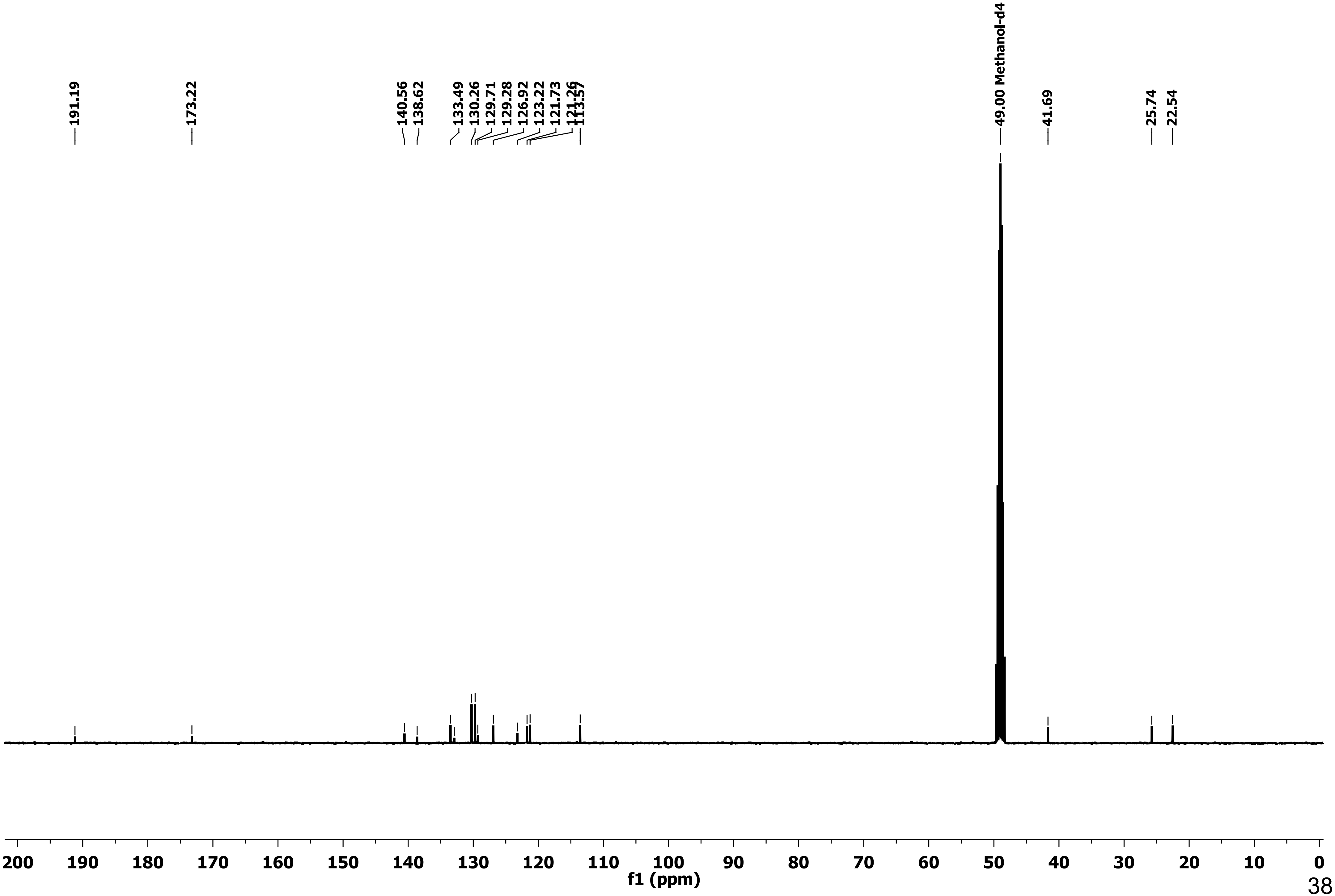
191.19
173.22
140.56
138.62
133.49
130.26
129.71
129.28
126.92
123.22
121.73
121.26
113.57
49.00 Methanol-d4
41.69
25.74
22.54
200
190
180
170
160
150
140
130
120
110
100
90
80
70
60
50
40
30
20
10
0
f1 (ppm)

8l

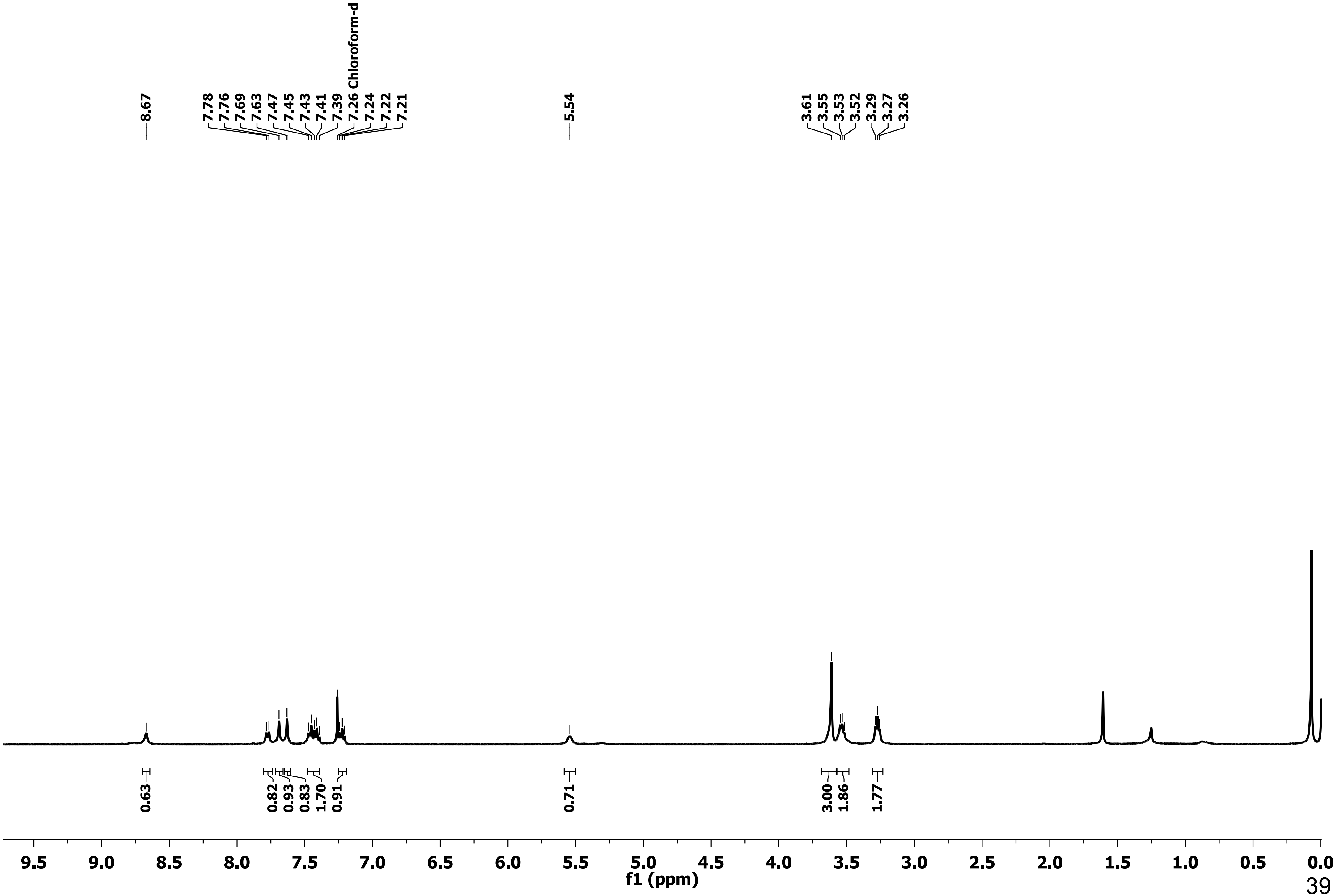

8.67
7.78
7.76
7.69
7.63
7.47
7.45
7.43
7.41
7.39
7.26 Chloroform-d
7.24
7.22
7.21
5.54
3.61
3.55
3.53
3.52
3.29
3.27
3.26
0.63
0.82
0.93
0.83
1.70
0.91
0.71
3.00
1.86
1.77
9.5 9.0 8.5 8.0 7.5 7.0 6.5 6.0 5.5 5.0 4.5 4.0 3.5 3.0 2.5 2.0 1.5 1.0 0.5 0.0
f1 (ppm)

8l

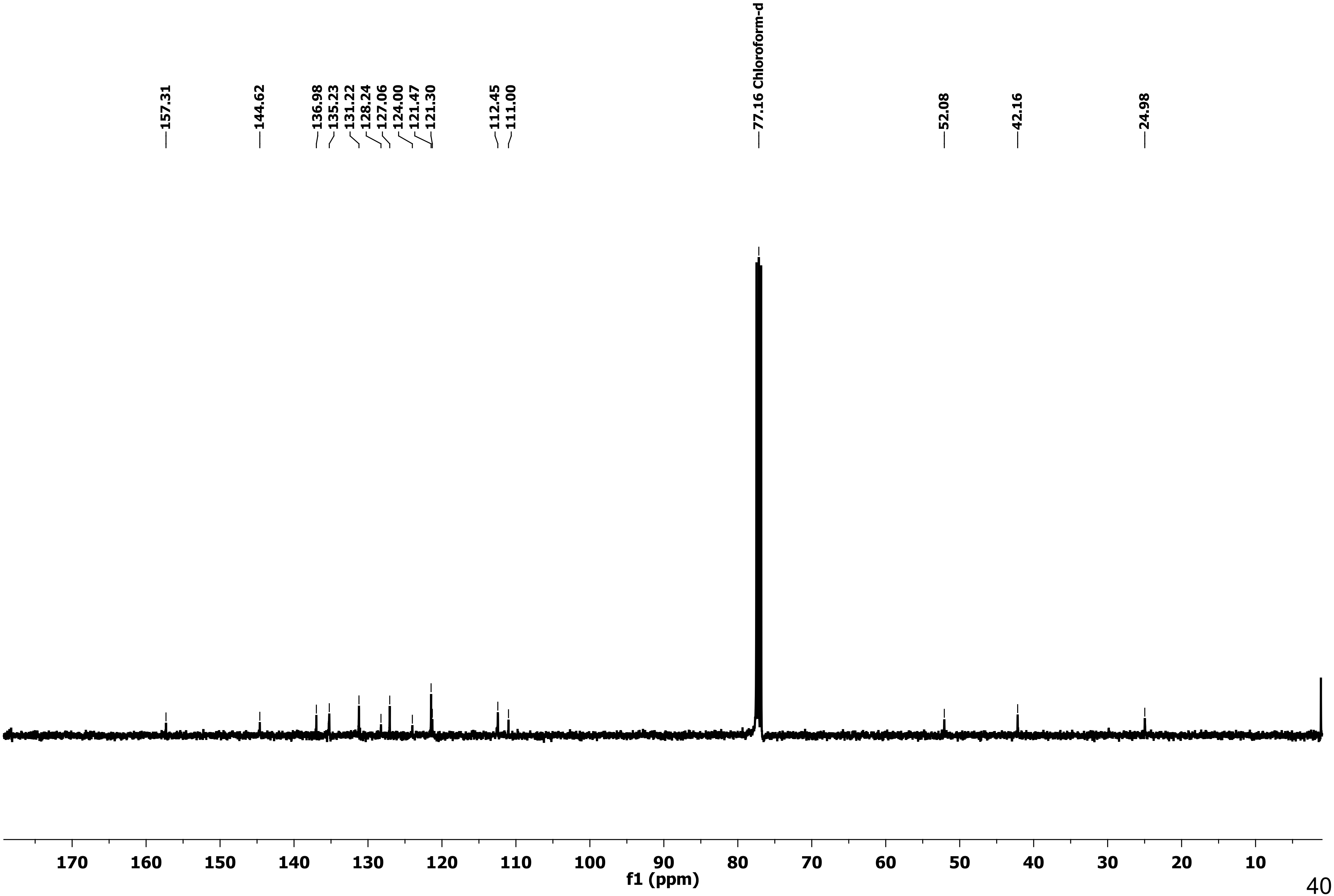
157.31
144.62
136.98
135.23
131.22
128.24
127.06
124.00
121.47
121.30
112.45
111.00
77.16 Chloroform-d
52.08
42.16
24.98
170
160
150
140
130
120
110
100
90
80
70
60
50
40
30
20
10
f1 (ppm)

8m

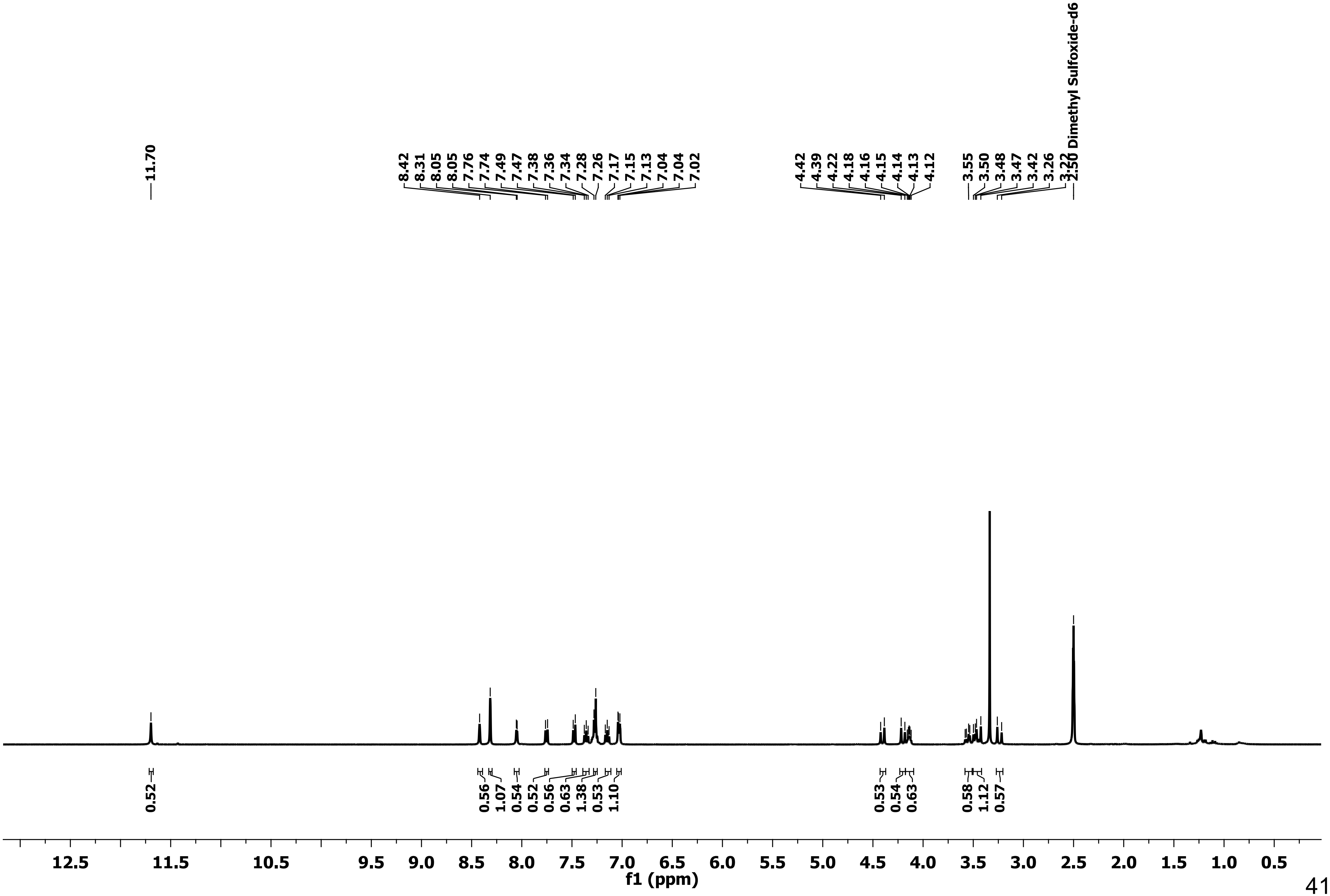
11.70
8.42
8.31
8.05
8.05
7.76
7.74
7.49
7.47
7.38
7.36
7.34
7.28
7.26
7.17
7.15
7.13
7.04
7.04
7.02
4.42
4.39
4.22
4.18
4.16
4.15
4.14
4.13
4.12
3.55
3.50
3.48
3.47
3.42
3.26
3.22
2.50 Dimethyl Sulfoxide-d6
0.52
0.56
1.07
0.54
0.52
0.56
0.63
1.38
0.53
1.10
0.53
0.54
0.63
0.58
1.12
0.57
12.5
11.5
10.5
9.5
9.0
8.5
8.0
7.5
7.0
6.5
6.0
5.5
5.0
4.5
4.0
3.5
3.0
2.5
2.0
1.5
1.0
0.5
f1 (ppm)

8m

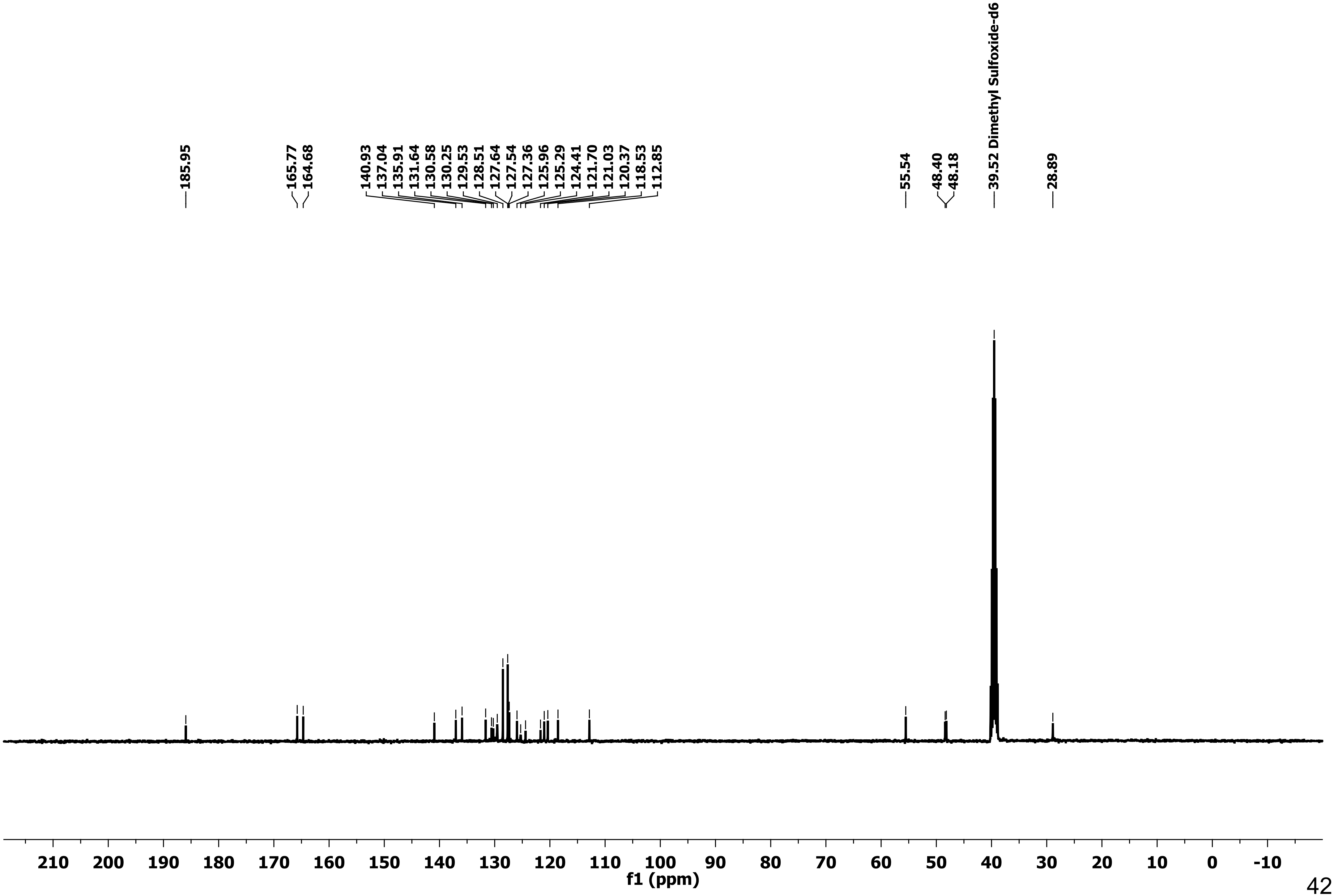

185.95
165.77
164.68
140.93
137.04
135.91
131.64
130.58
130.25
129.53
128.51
127.64
127.54
127.36
125.96
125.29
124.41
121.70
121.03
120.37
118.53
112.85
55.54
48.40
48.18
39.52 Dimethyl Sulfoxide-d6
28.89
210 200 190 180 170 160 150 140 130 120 110 100 90 80 70 60 50 40 30 20 10 0 -10
f1 (ppm)

8n

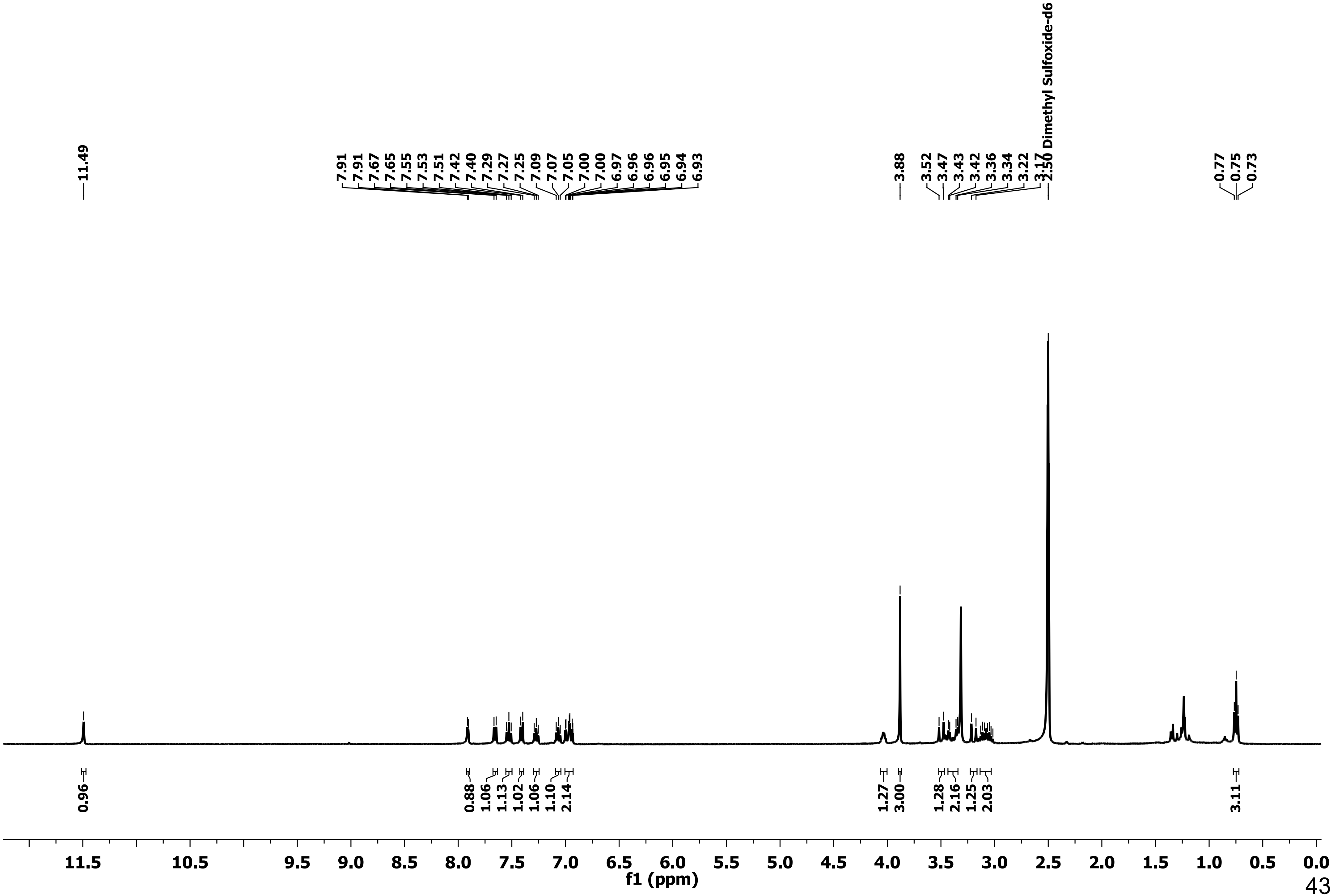
11.49
7.91
7.91
7.67
7.65
7.55
7.53
7.51
7.42
7.40
7.29
7.27
7.25
7.09
7.07
7.05
7.00
7.00
6.97
6.96
6.96
6.95
6.94
6.93
3.88
3.52
3.47
3.43
3.42
3.36
3.34
3.22
3.17
2.50 Dimethyl Sulfoxide-d6
0.77
0.75
0.73
0.96
0.88
1.06
1.13
1.02
1.06
1.10
2.14
1.27
3.00
1.28
2.16
1.25
2.03
3.11
11.5
10.5
9.5
9.0
8.5
8.0
7.5
7.0
6.5
6.0
5.5
5.0
4.5
4.0
3.5
3.0
2.5
2.0
1.5
1.0
0.5
0.0
f1 (ppm)

8n

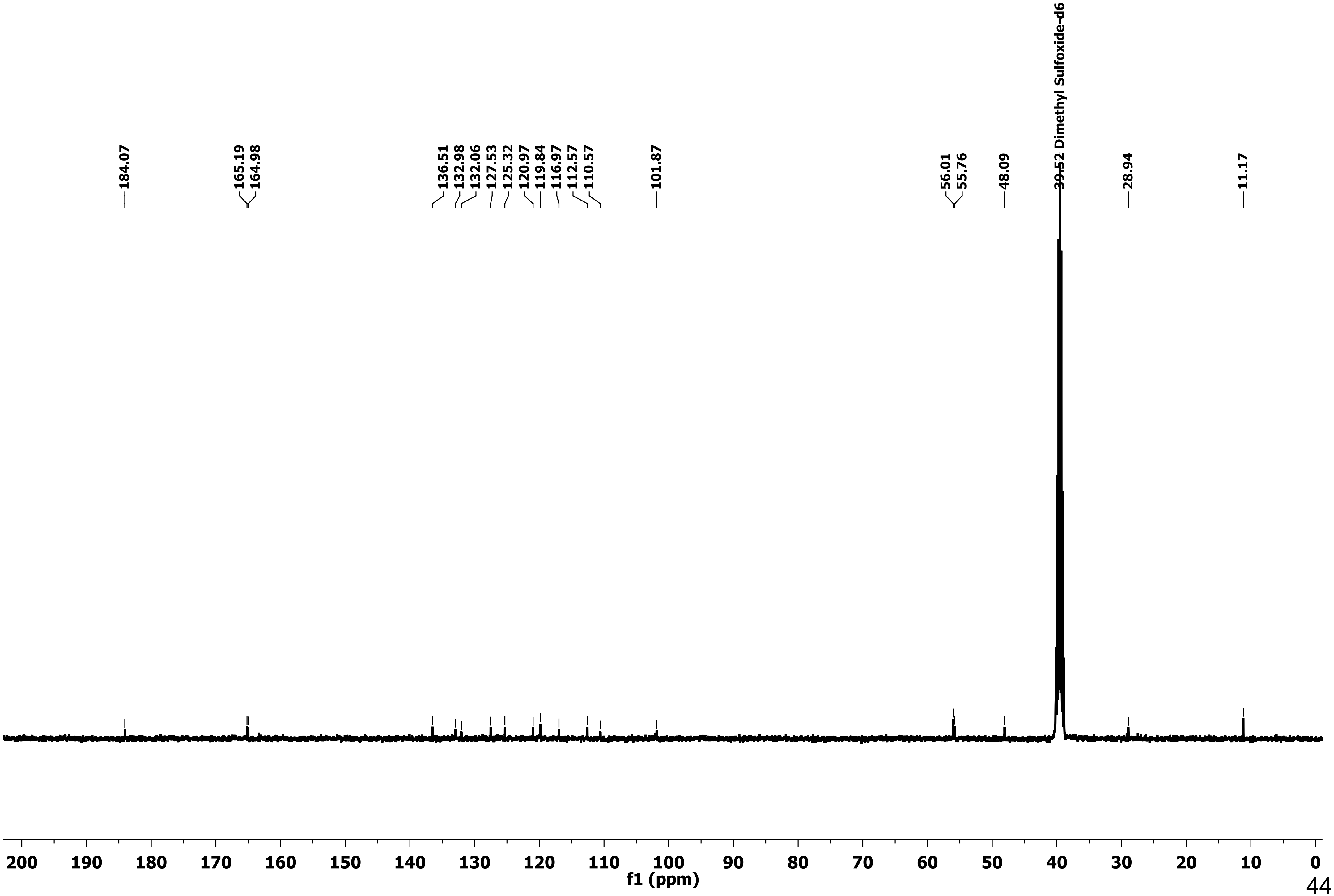
184.07
165.19
164.98
136.51
132.98
132.06
127.53
125.32
120.97
119.84
116.97
112.57
110.57
101.87
56.01
55.76
48.09
39.52 Dimethyl Sulfoxide-d6
28.94
11.17
200 190 180 170 160 150 140 130 120 110 100 90 80 70 60 50 40 30 20 10 0
f1 (ppm)

8o

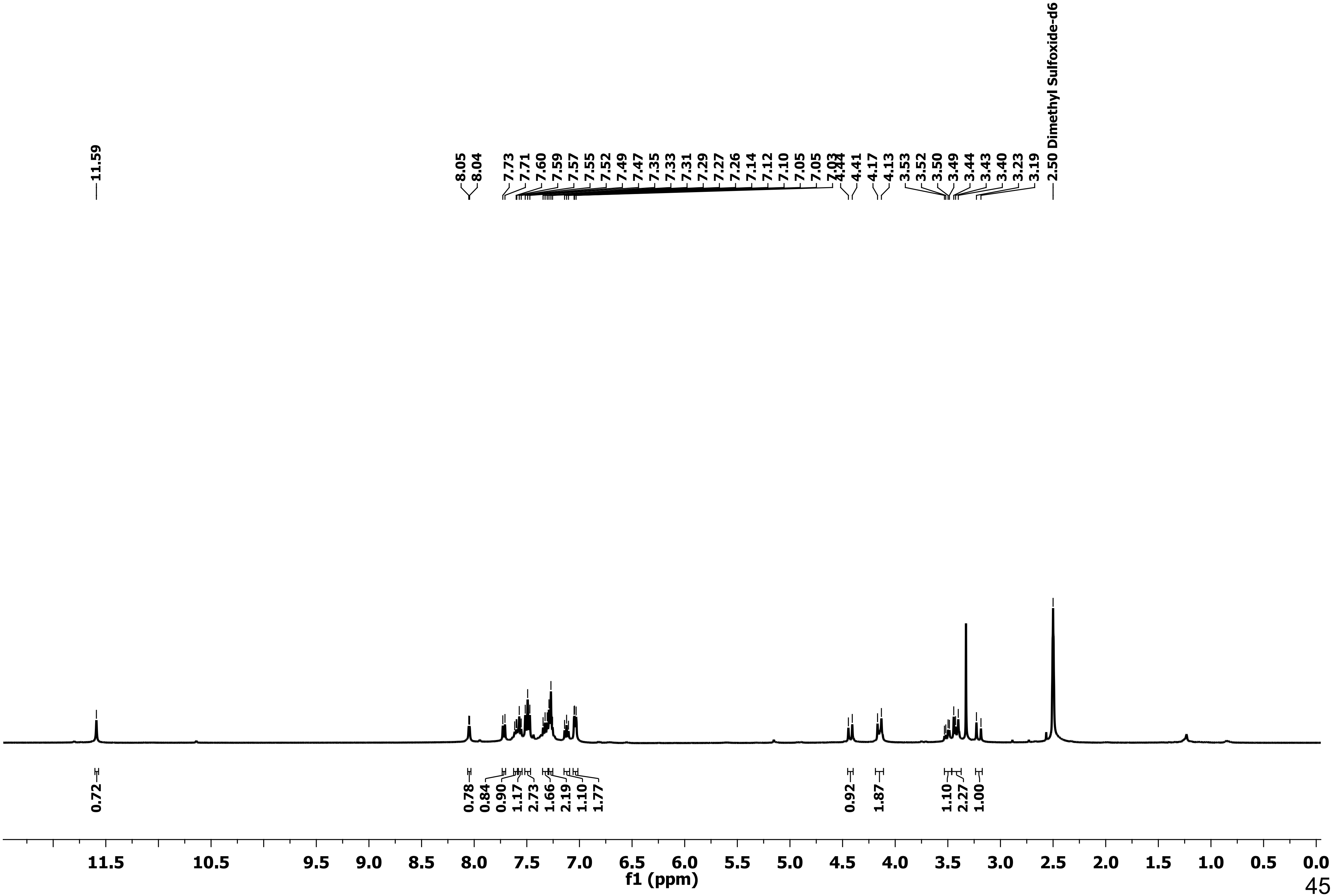

11.59
8.05
8.04
7.73
7.71
7.60
7.59
7.57
7.55
7.52
7.49
7.47
7.35
7.33
7.31
7.29
7.27
7.26
7.14
7.12
7.10
7.05
7.05
7.03
4.44
4.41
4.17
4.13
3.53
3.52
3.50
3.49
3.44
3.43
3.40
3.23
3.19
2.50 Dimethyl Sulfoxide-d6
0.72
0.78
0.84
0.90
1.17
2.73
1.66
2.19
1.10
1.77
0.92
1.87
1.10
2.27
1.00
11.5
10.5
9.5
9.0
8.5
8.0
7.5
7.0
6.5
6.0
5.5
5.0
4.5
4.0
3.5
3.0
2.5
2.0
1.5
1.0
0.5
0.0
f1 (ppm)

8o

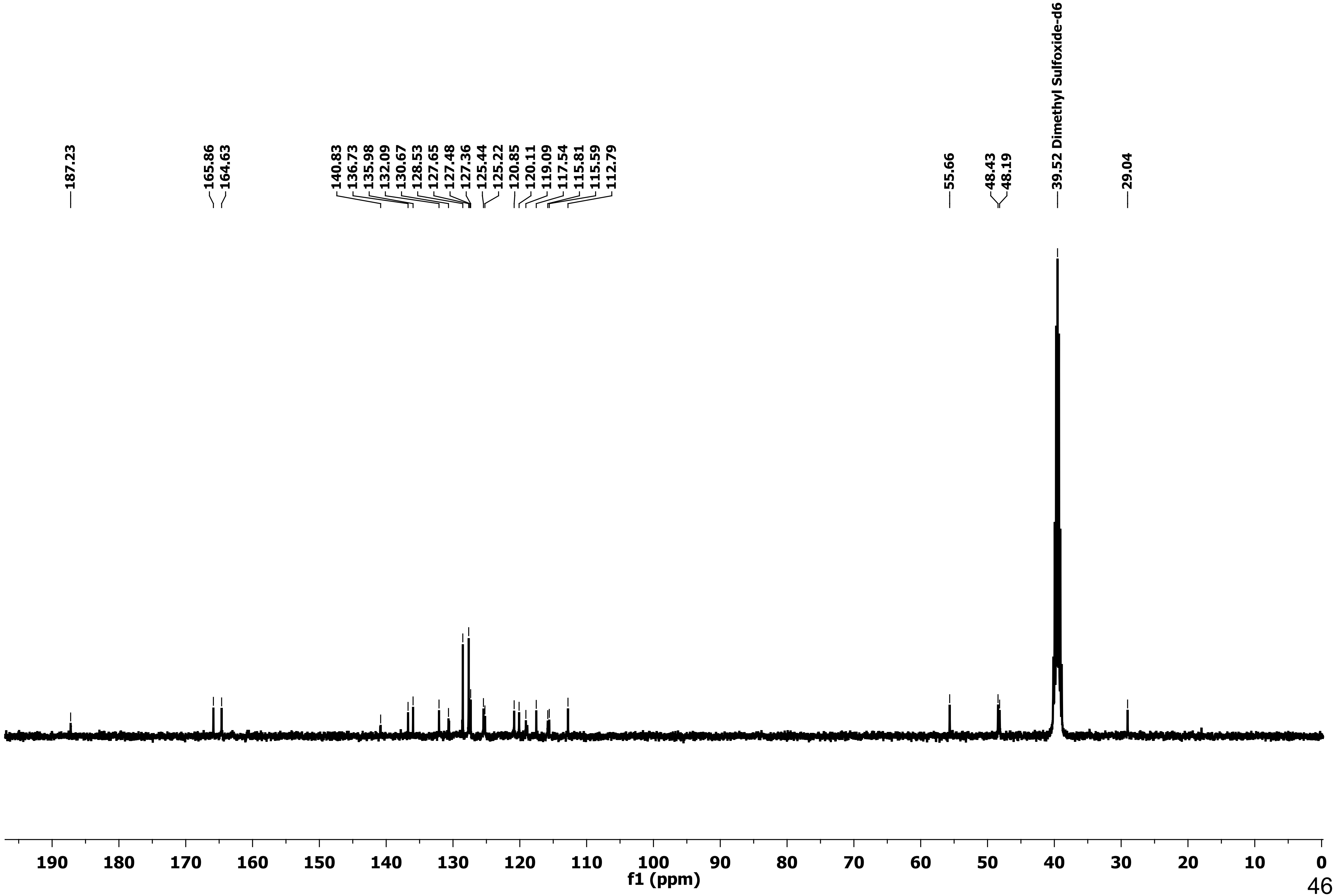

187.23
165.86
164.63
140.83
136.73
135.98
132.09
130.67
128.53
127.65
127.48
127.36
125.44
125.22
120.85
120.11
119.09
117.54
115.81
115.59
112.79
55.66
48.43
48.19
39.52 Dimethyl Sulfoxide-d6
29.04
190 180 170 160 150 140 130 120 110 100 90 80 70 60 50 40 30 20 10 0
f1 (ppm)

8p

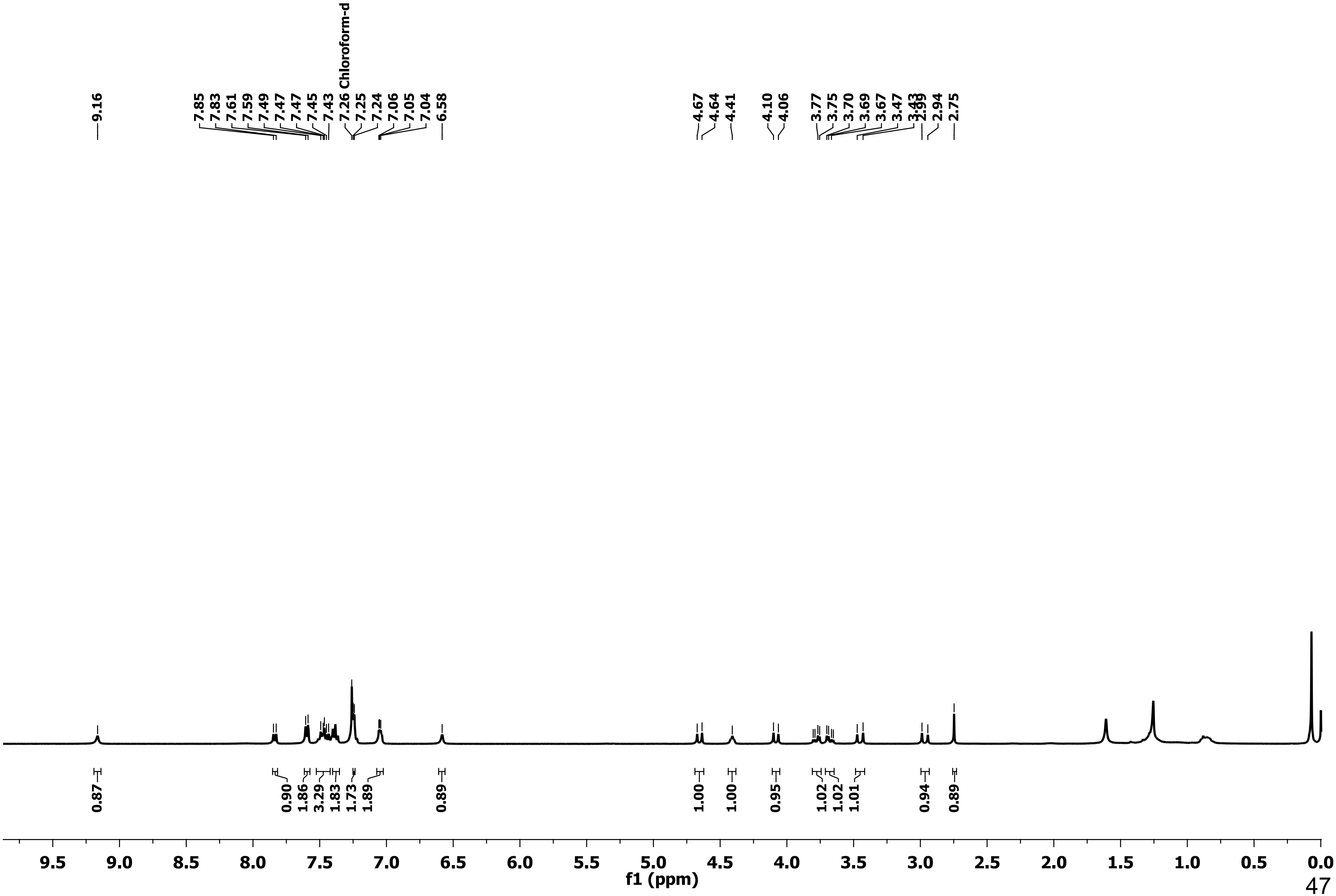

9.16
7.85
7.83
7.61
7.59
7.49
7.47
7.47
7.45
7.43
7.26 Chloroform-d
7.25
7.24
7.06
7.05
7.04
6.58
4.67
4.64
4.41
4.10
4.06
3.77
3.75
3.70
3.69
3.67
3.47
3.43
2.99
2.94
2.75
0.87
0.90
1.86
3.29
1.83
1.73
1.89
0.89
1.00
1.00
0.95
1.02
1.02
1.01
0.94
0.89
9.5
9.0
8.5
8.0
7.5
7.0
6.5
6.0
5.5
5.0
4.5
4.0
3.5
3.0
2.5
2.0
1.5
1.0
0.5
0.0
f1 (ppm)

8p

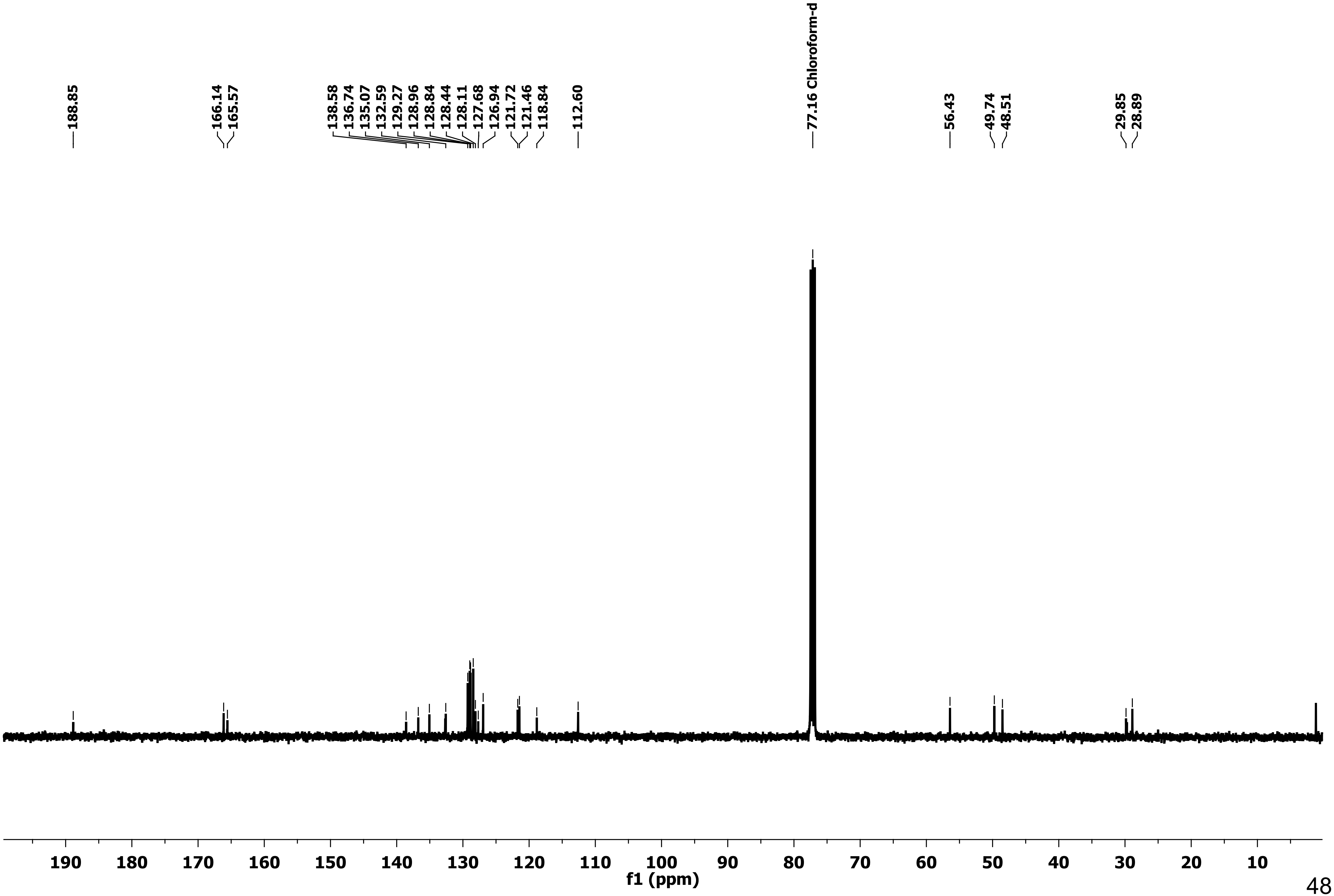
188.85
166.14
165.57
138.58
136.74
135.07
132.59
129.27
128.96
128.84
128.44
128.11
127.68
126.94
121.72
121.46
118.84
112.60
77.16 Chloroform-d
56.43
49.74
48.51
29.85
28.89
190 180 170 160 150 140 130 120 110 100 90 80 70 60 50 40 30 20 10
f1 (ppm)

Acquisition Method Report

Acquisition Method Info

**Method Name**	jyoti ido method 1.m
**Method Path**	D:\MassHunter\Methods\jyoti ido method 1.m
**Method Description**	Default Method

Device List

HiP Sampler
Binary Pump
Column Comp.
DAD
Q-TOF

TOF/Q-TOF Mass Spectrometer

**Component Name**	MS Q-TOF	**Component Model**	G6540B
**Ion Source**	Dual AJS ESI	**Stop Time (min)**	No Limit/As Pump
**Can wait for temp.**	Enable	**Fast Polarity**	N/A
**MS Abs. threshold**	200	**MS Rel. threshold(%)**	0.010
**MS/MS Abs. threshold**	5	**MS/MS Rel. threshold(%)**	0.010
**Tune File**	Autotune.tun		

Time Segments

Time Segment #	Start Time (min)	Diverter Valve State	Storage Mode	Ion Mode
1	0	Waste	Both	Dual AJS ESI
2	2.4	MS	Both	Dual AJS ESI

Time Segment 1

Acquisition Mode MS1

**Min Range (m/z)**	125
**Max Range (m/z)**	500
**Scan Rate (spectra/sec)**	2.00

Source Parameters

Parameter	Value
Gas Temp (°C)	310
Gas Flow (l/min)	10
Nebulizer (psig)	45
SheathGasTemp	300
SheathGasFlow	10

Scan Segments

Scan Seg #	Ion Polarity	Collision Energy
1	Positive	0

Scan Segment 1

Scan Source Parameters

Parameter	Value
VCap	3500
Nozzle Voltage (V)	1000
Fragmentor	80
Skimmer1	70
OctopoleRFPeak	750

Time Segment 2

Acquisition Mode MS1

**Min Range (m/z)**	125
**Max Range (m/z)**	500
**Scan Rate (spectra/sec)**	2.00

Source Parameters

Parameter	Value
Gas Temp (°C)	310
Gas Flow (l/min)	10
Nebulizer (psig)	45
SheathGasTemp	300
SheathGasFlow	10

Scan Segments

Scan Seg #	Ion Polarity	Collision Energy
1	Positive	0

Scan Segment 1

Scan Source Parameters

Parameter	Value
VCap	3500
Nozzle Voltage (V)	1000
Fragmentor	80
Skimmer1	70
OctopoleRFPeak	750

ReferenceMasses

**Ref Mass Enabled**	Disabled
**Ref Nebulizer (psig)**	

Chromatograms

Chrom Type	Label	Offset	Y-Range
TIC	TIC	0.01	100

Name: HiP Sampler	Model: G4226A
**Auxiliary**	
Draw Speed	100.0 µL/min
Eject Speed	400.0 µL/min
Draw Position Offset	0.0 mm
Wait Time After Drawing	1.2 s
Sample Flush Out Factor	5.0
Vial/Well bottom sensing	No
**Injection**	
Injection Mode	Injection with needle wash
Injection Volume	1.00 µL
**Needle Wash**	
Needle Wash Location	Flush Port
Wash Time	50.0 s
**High throughput**	
Automatic Delay Volume Reduction	No
**Overlapped Injection**	
Enable Overlapped Injection	No
**Valve Switching**	
Valve Movements	1
**Valve Switch Time 1**	
Switch Time 1 Enabled	No
**Valve Switch Time 2**	
Switch Time 2 Enabled	No
**Valve Switch Time 3**	
Switch Time 3 Enabled	No
**Valve Switch Time 4**	
Switch Time 4 Enabled	No
**Stop Time**	
Stoptime Mode	As pump/No limit
**Post Time**	
Posttime Mode	Off

Name: Binary Pump	Model: G4220B
Flow	0.300 mL/min
Use Solvent Types	Yes
Stroke Mode	Synchronized
Low Pressure Limit	0.00 bar
High Pressure Limit	400.00 bar
Max. Flow Ramp Up	100.000 mL/min²
Max. Flow Ramp Down	100.000 mL/min²
Expected Mixer	No check
**Stroke A**	
Automatic Stroke Calculation A	Yes
**Compress A**	
Compressibility Mode A	Compressibility Value Set
Compressibility A	45 10e-6/bar
**Compress B**	
Compressibility Mode B	Compressibility Value Set
Compressibility B	75 10e-6/bar
**Stop Time**	
Stoptime Mode	Time set
Stoptime	8.00 min
**Post Time**	
Posttime Mode	Off

Timetable

Timetable

	Time	Function	Parameter
1	1.00 min	Change Solvent Composition	Solvent composition A: 98.00 % B:2.00 %
2	1.00 min	Change Flow	Flow: 0.3 mL/min
3	1.00 min	Change Max. Pressure Limit	Max. Pressure Limit: 400.00 bar
4	5.00 min	Change Solvent Composition	Solvent composition A: 5.00 % B:95.00 %
5	5.00 min	Change Flow	Flow: 0.3 mL/min
6	5.00 min	Change Max. Pressure Limit	Max. Pressure Limit: 400.00 bar
7	6.00 min	Change Solvent Composition	Solvent composition A: 5.00 % B:95.00 %
8	6.00 min	Change Flow	Flow: 0.3 mL/min
9	6.00 min	Change Max. Pressure Limit	Max. Pressure Limit: 400.00 bar
10	7.00 min	Change Solvent Composition	Solvent composition A: 98.00 % B:2.00 %
11	7.00 min	Change Flow	Flow: 0.3 mL/min
12	7.00 min	Change Max. Pressure Limit	Max. Pressure Limit: 400.00 bar
13	8.00 min	Change Solvent Composition	Solvent composition A: 98.00 % B:2.00 %
14	8.00 min	Change Flow	Flow: 0.3 mL/min
15	8.00 min	Change Max. Pressure Limit	Max. Pressure Limit: 400.00 bar

Solvent Composition

	Channel	Ch. 1 Solv.	Name 1	Ch2 Solv.	Name 2	Selected	Used	Percent
1	A	100.0 % Water V.03	0.1% FA	100.0 % Water V.03		Ch. 1	Yes	98.00 %
2	B	100.0 % Acetonitrile V.03		100.0 % Acetonitrile V.03	0.1% FA	Ch. 2	Yes	2.00 %

Name: Column Comp. Model: G1316C

Ready when front door open Yes

Left Temperature Control

Temperature Control Mode Not Controlled

Enable Analysis Left Temperature

Enable Analysis Left Temperature On Yes
Enable Analysis Left Temperature Value 0.80 °C

Right Temperature Control

Right temperature Control Mode Not Controlled

Enable Analysis Right Temperature

Enable Analysis Right Temperature On Yes
Enable Analysis Right Temperature Value 0.80 °C

Stop Time

Stoptime Mode As pump/injector

Post Time

Posttime Mode Off

Name: DAD   Model: G4212A

**Peakwidth**	>0.10 min (2.0 s response time) (2.5 Hz)
**Slit**	4 nm
**UV Lamp Required**	No

Analog Output 1

**Analog 1 Zero Offset**	5 %
**Analog 1 Attenuation**	1000 mAU

Signals

Prepare Mode

**Margin for negative Absorbance**	100 mAU

Autobalance

**Autobalance Prerun**	No
**Autobalance Postrun**	No

Spectrum

**Spectrum Store**	None

Stoptime

**Stoptime Mode**	As pump/injector

Posttime

**Posttime Mode**	Off

Signals

Signal table

	Use Sig.	Signal
1	No	Signal A
2	No	Signal B
3	No	Signal C
4	No	Signal D
5	No	Signal E
6	No	Signal F
7	No	Signal G
8	No	Signal H

Acquisition Method Report

Acquisition Method Info

**Method Name**	jyoti ido method 1-Neg-8R-msms.m
**Method Path**	D:\MassHunter\Methods\jyoti ido method 1-Neg-8R-msms.m
**Method Description**	Default Method

Device List

- HiP Sampler
- Binary Pump
- Column Comp.
- DAD
- Q-TOF

TOF/Q-TOF Mass Spectrometer

**Component Name**	MS Q-TOF	**Component Model**	G6540B
**Ion Source**	Dual AJS ESI	**Stop Time (min)**	No Limit/As Pump
**Can wait for temp.**	Enable	**Fast Polarity**	N/A
**MS Abs. threshold**	200	**MS Rel. threshold(%)**	0.010
**MS/MS Abs. threshold**	5	**MS/MS Rel. threshold(%)**	0.010
**Tune File**	Autotune.tun		

Time Segments

Time Segment #	Start Time (min)	Diverter Valve State	Storage Mode	Ion Mode
1	0	MS	Both	Dual AJS ESI

Time Segment 1

Acquisition Mode TargetedMS2

**MS Min Range (m/z)**	50
**MS Max Range (m/z)**	1000
**MS Scan Rate (spectra/sec)**	2.00
**MS/MS Scan Rate (spectra/sec)**	2.00
**Max Time Between MS (sec)**	0.0
**Use Fixed Collision Energies**	35.00

Targeted Mass Table

Mass	Z	Ret. Time (min)	Delta Ret. Time (min)	Isolation Width	Collision Energy	Acq. Time (ms/spec)
382.1083	1	6	2	Medium (~4 amu)	30	
350.1192	1	5.5	2	Medium (~4 amu)	30	
366.1126	1	5.75	2	Medium (~4 amu)	30	

Source Parameters

Parameter	Value
Gas Temp (°C)	310
Gas Flow (l/min)	10
Nebulizer (psig)	45
SheathGasTemp	300
SheathGasFlow	10

Scan Segments

Scan Seg #	Ion Polarity	Collision Energy
1	Negative	0

Scan Segment 1

Scan Source Parameters

Parameter	Value
VCap	3500
Nozzle Voltage (V)	1000
Fragmentor	80
Skimmer1	70
OctopoleRFPeak	750

ReferenceMasses

**Ref Mass Enabled**	Disabled
**Ref Nebulizer (psig)**	

Chromatograms

Chrom Type	Label	Offset	Y-Range
TIC	TIC	0.01	100

Name: HiP Sampler Model: G4226A

**Auxiliary**	
Draw Speed	100.0 µL/min
Eject Speed	400.0 µL/min
Draw Position Offset	0.0 mm
Wait Time After Drawing	1.2 s
Sample Flush Out Factor	5.0
Vial/Well bottom sensing	No
**Injection**	
Injection Mode	Injection with needle wash
Injection Volume	1.00 µL
**Needle Wash**	
Needle Wash Location	Flush Port
Wash Time	50.0 s
**High throughput**	
Automatic Delay Volume Reduction	No
**Overlapped Injection**	
Enable Overlapped Injection	No
**Valve Switching**	
Valve Movements	1
**Valve Switch Time 1**	
Switch Time 1 Enabled	No
**Valve Switch Time 2**	
Switch Time 2 Enabled	No
**Valve Switch Time 3**	
Switch Time 3 Enabled	No
**Valve Switch Time 4**	
Switch Time 4 Enabled	No
**Stop Time**	
Stoptime Mode	As pump/No limit
**Post Time**	
Posttime Mode	Off

Name: Binary Pump Model: G4220B

Flow	0.300 mL/min
Use Solvent Types	Yes
Stroke Mode	Synchronized
Low Pressure Limit	0.00 bar
High Pressure Limit	400.00 bar
Max. Flow Ramp Up	100.000 mL/min²
Max. Flow Ramp Down	100.000 mL/min²
Expected Mixer	No check
**Stroke A**	
Automatic Stroke Calculation A	Yes
**Compress A**	
Compressibility Mode A	Compressibility Value Set
Compressibility A	45 10e-6/bar
**Compress B**	
Compressibility Mode B	Compressibility Value Set
Compressibility B	75 10e-6/bar
**Stop Time**	
Stoptime Mode	Time set
Stoptime	8.00 min
**Post Time**	
Posttime Mode	Off

Timetable

Timetable

	Time	Function	Parameter
1	1.00 min	Change Solvent Composition	Solvent composition A: 98.00 % B:2.00 %
2	1.00 min	Change Flow	Flow: 0.3 mL/min
3	1.00 min	Change Max. Pressure Limit	Max. Pressure Limit: 400.00 bar
4	5.00 min	Change Solvent Composition	Solvent composition A: 5.00 % B:95.00 %
5	5.00 min	Change Flow	Flow: 0.3 mL/min
6	5.00 min	Change Max. Pressure Limit	Max. Pressure Limit: 400.00 bar
7	6.00 min	Change Solvent Composition	Solvent composition A: 5.00 % B:95.00 %
8	6.00 min	Change Flow	Flow: 0.3 mL/min
9	6.00 min	Change Max. Pressure Limit	Max. Pressure Limit: 400.00 bar
10	7.00 min	Change Solvent Composition	Solvent composition A: 98.00 % B:2.00 %
11	7.00 min	Change Flow	Flow: 0.3 mL/min
12	7.00 min	Change Max. Pressure Limit	Max. Pressure Limit: 400.00 bar
13	8.00 min	Change Solvent Composition	Solvent composition A: 98.00 % B:2.00 %
14	8.00 min	Change Flow	Flow: 0.3 mL/min
15	8.00 min	Change Max. Pressure Limit	Max. Pressure Limit: 400.00 bar

Solvent Composition

	Channel	Ch. 1 Solv.	Name 1	Ch2 Solv.	Name 2	Selected	Used	Percent
1	A	100.0 % Water V.03	0.1% FA	100.0 % Water V.03		Ch. 1	Yes	98.00 %
2	B	100.0 % Acetonitrile V.03		100.0 % Acetonitrile V.03	0.1% FA	Ch. 2	Yes	2.00 %

Name: Column Comp. Model: G1316C

**Ready when front door open**	Yes
**Left Temperature Control**	
**Temperature Control Mode**	Not Controlled
**Enable Analysis Left Temperature**	
**Enable Analysis Left Temperature On**	Yes
**Enable Analysis Left Temperature Value**	0.80 °C
**Right Temperature Control**	
**Right temperature Control Mode**	Not Controlled
**Enable Analysis Right Temperature**	
**Enable Analysis Right Temperature On**	Yes
**Enable Analysis Right Temperature Value**	0.80 °C
**Stop Time**	
**Stoptime Mode**	As pump/injector
**Post Time**	
**Posttime Mode**	Off

Name: DAD **Model:** G4212A

Peakwidth	>0.10 min (2.0 s response time) (2.5 Hz)
Slit	4 nm
UV Lamp Required	No

Analog Output 1

Analog 1 Zero Offset	5 %
Analog 1 Attenuation	1000 mAU

Signals

Prepare Mode

Margin for negative Absorbance	100 mAU

Autobalance

Autobalance Prerun	No
Autobalance Postrun	No

Spectrum

Spectrum Store	None

Stoptime

Stoptime Mode	As pump/injector

Posttime

Posttime Mode	Off

Signals

Signal table

	Use Sig.	Signal
1	No	Signal A
2	No	Signal B
3	No	Signal C
4	No	Signal D
5	No	Signal E
6	No	Signal F
7	No	Signal G
8	No	Signal H
